# The global, regional, and national preventable burden of depression attributable to greenness and inequalities: a scenario-based health impact analysis

**DOI:** 10.7189/jogh.15.04280

**Published:** 2025-10-03

**Authors:** Jiayu Wu, Wenjie Di, Jidian Ruan, Senhao Li, Jiayao Ying, Jiali Zhou, Igor Rudan, Peige Song

**Affiliations:** 1 College of Agriculture and Biotechnology, Zhejiang University, Hangzhou, China; 2 School of Public Health and the Second Affiliated Hospital, Zhejiang University School of Medicine, Hangzhou, China; 3 Centre for Global Health, Usher Institute, University of Edinburgh, Edinburgh, Scotland, UK; 4 Nuffield Department of Primary Care Health Sciences, Oxford University, UK

## Abstract

**Background:**

Growing evidence suggests that exposure to greenness may reduce the burden of depression. Here, we quantified the global, regional, and national preventable burden of depression attributable to greenness and examined the associated socioeconomic inequalities.

**Methods:**

We conducted a scenario-based health impact assessment using data from the Global Burden of Disease 2021 study to estimate the preventable burden of depression (measured in disability-adjusted life years (DALYs)) under three greenness expansion scenarios. We performed a random-effects meta-analysis to derive a pooled odds ratio (OR) for the association between greenness and depression, adjusting it for city-specific greenness levels to calculate the population attributable fraction for each country. We assessed temporal trends (2001–20), analysed sex differences, and quantified cross-country inequalities using the slope index of inequality and concentration index.

**Results:**

Under the best potential scenario, the global age-standardised DALY rate (ASDR) of preventable depression per 100 000 population increased from 93.60 (95% uncertainty interval (UI) = 87.79, 99.42) in 2001 to 117.67 (95% UI = 110.61, 124.72) in 2020, with an average annual percentage change (AAPC) of 1.13% (95% confidence interval (CI) = 0.52, 1.75). Regionally, the African Region exhibited the highest growth (AAPC = 1.78%), while the Americas had the highest preventable burden in 2020 of 287.83 (95% UI = 252.67, 322.99). Moreover, the burden varied across sociodemographic index regions. Females consistently exhibited a higher preventable burden than males, with an absolute sex difference in ASDR of 54.40 (95% CI = 44.67, 64.48) in 2020. Cross-country inequalities narrowed globally, with the concentration index declining from 0.160 (95% CI = 0.088, 0.232) in 2001 to 0.051 (95% CI = −0.021, 0.123) in 2020, though regional disparities persisted.

**Conclusions:**

Greenness expansion has the potential to significantly reduce the global depression burden, but inequitable access exacerbates mental health disparities. Targeted urban greening policies are needed to enhance mental well-being and health equity worldwide.

Depression is a leading cause of disability worldwide, affecting more than 280 million people [1] and contributing significantly to the global burden of disease. As a complex mental disorder, depression not only diminishes individuals' quality of life, but also increases the risk of premature mortality and comorbidities such as cardiovascular disease [2–4]. The aetiology of depression is multifactorial [5] and involves genetic predisposition [6], neurobiological mechanisms [7], psychological stressors [8], and environmental influences [9,10]. Despite advances in understanding the clinical and individual-level risk factors for depression, its growing prevalence underscores the need to explore more modifiable environmental determinants embedded in modern living contexts to inform effective public health interventions [5].

An environmental factor gaining increasing attention is exposure to greenness, *i.e. *natural spaces such as parks, forests, and green areas in urban settings. Evidence suggests that exposure to greenness can have a positive impact on mental health [11,12] and could reduce the risk of depression and other psychiatric disorders [13,14]. Potential mechanisms behind this relationship include stress reduction, promotion of physical activity, and enhanced social cohesion [14]. Importantly, greenness is modifiable at both local and national levels, making it an achievable public health intervention target [12]. Urban planning, environmental policies, and sustainable development initiatives can directly influence the availability, accessibility, and quality of green spaces, offering a scalable, long-term strategy to mitigate mental health burdens.

However, global access to greenness is not equitably distributed across regions and populations [15]. High-income countries generally benefit from greater availability and higher quality of green spaces compared to low- and middle-income countries, where rapid urbanisation, environmental degradation, and inadequate infrastructure often limit access to green spaces, particularly in densely populated urban areas [16]. Even within countries, socioeconomic disparities further exacerbate inequities, with marginalised communities often disproportionately experiencing reduced exposure to green spaces [16,17]. The association between greenness and depression has been well-established [13]. For this reason, inequities in access pose not merely environmental issues, but also have important implications for health equity, as mental health benefits of greenness may be unevenly realised across populations and regions.

Despite these insights, the potential health impact of increasing greenness exposure at the global, regional, and national levels remains largely unknown. Specifically, it is unclear how much of the global burden of depression could be averted if countries at different levels of development and urbanisation implemented policies to improve greenness exposure. Additionally, the implications of such efforts for addressing health inequities have not been systematically explored. This leaves a need for a comparative, scenario-based analysis to quantify the potential mental health benefits of enhancing greenness across varying contexts.

Health impact assessment (HIA) is a widely used epidemiological approach that enables the estimation of anticipated effects of environmental changes (*e.g.* improving greenness) on population health outcomes [18–20]. By integrating data on greenness distribution, the association between greenness and depression, and population exposure levels, HIA offers a robust framework for quantifying how much of the burden of depression could be prevented under different greenness improvement scenarios. Here, we applied a scenario-based HIA to estimate the preventable global, regional, and national burden of depression attributable to increased greenness exposure.

## METHODS

**Figure 1:**
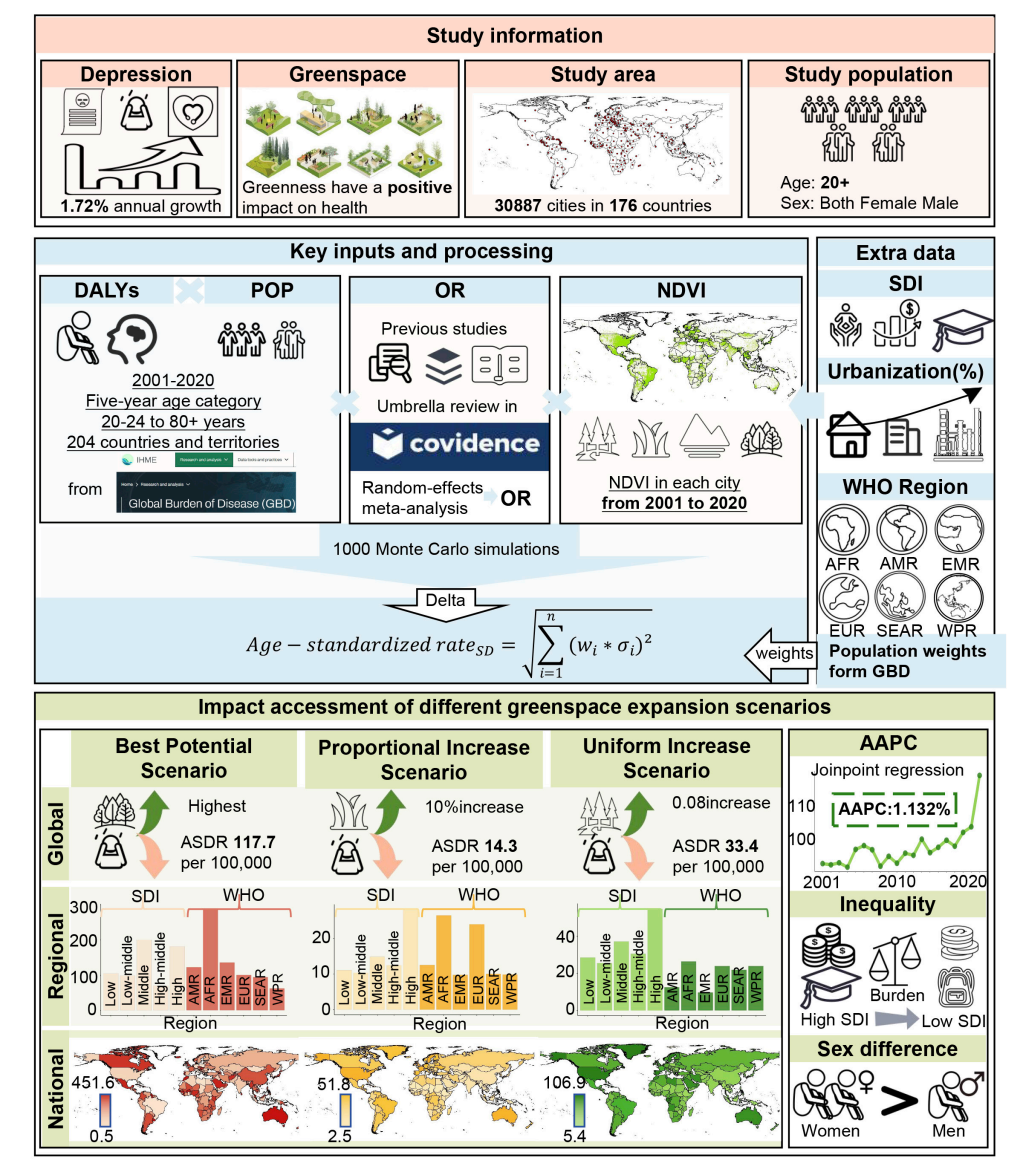
HIA analytical framework diagram. AAPC – average annual percentage changes, AFR – African region, AMR – region of the Americas, ASDR – age-standardised DALY rates, DALY – disability-adjusted life year, EMR – Eastern Mediterranean region, EUR – European region, HIA – health impact assessment, NDVI – normalised difference vegetation index, OR – odds ratio, SDI – sociodemographic index, SEAR – South-East Asia region, WHO – World Health Organization, WPR – Western Pacific region.

We estimated the potential preventable burden of depression attributable to increases in green space where data were available (**Figure 1**). We used a scenario-based HIA approach to quantify the preventable burden of depression attributable to green space expansion, following a methodology similar to that of Barboza and colleagues [21]. We employed a linear HIA function to estimate the change in depression burden associated with incremental increases in green space exposure. First, we derived a pooled odds ratio (OR) from a meta-analysis quantifying the association between green space (measured by the normalised difference vegetation index (NDVI)) and depression. We adjusted this pooled OR to city-specific ORs by incorporating city-level green space exposure and population characteristics. Using these city-specific ORs and the proportion of the population exposed to suboptimal green space, we calculated the population attributable fraction for each country under three counterfactual green space expansion scenarios: the best potential scenario (*i.e.* maximum achievable green space expansion), the proportional increase scenario (*i.e.* proportional increases based on current green space levels), and the uniform increase scenario (*i.e.* equal green space expansion across all cities).

We then applied the country-specific population attributable fractions to the depressive-related burden, measured in disability-adjusted life years (DALYs) in the Global Burden of Disease (GBD) 2021 study [22], to quantify the preventable burden of depression at the national, regional, and global levels if greenness levels were increased to predefined targets.

### Association between green space exposure and depression

We identified relevant studies that linked green space exposure with depression through a two-stage process. First, we performed an umbrella review to identify systematic reviews and meta-analyses on the association between green space (*e.g.* NDVI, enhanced vegetation index (EVI)) and depression. We searched four databases – PubMed, Embase, MEDLINE, and Web of Science – covering the 2020–23 period, with the MEDLINE searches extending back to 1990. We combined search terms related to greenness (*e.g.* green space, NDVI, EVI) and review types (*e.g.* review, meta-analysis), with no language restrictions (Appendix S1 in the **Online Supplementary Document**). In the second stage, we retrieved all original articles from the selected reviews, including them based on the population, exposure, comparator, outcome, and study design framework:

– Population: human populations, regardless of age, sex, race, geographical region, or health status;

– Exposure: any type of green space exposure (*e.g.* NDVI, EVI);

– Comparator: studies comparing health outcomes among individuals exposed to different levels of green space;

– Outcome: depression;

– Study design: systematic reviews, meta-analyses, and original studies.

We imported the records into Covidence for screening [23]. After duplicate removal, two reviewers (WD and JR) independently screened all records by title and abstract, followed by the full text of any remaining studies. In cases where multiple publications reported on the same study, we only included the most recent one, the one including more articles, or those that provided more detailed information. For each original article, two reviewers (WD and JR) independently extracted the following information:

– Article information: author, publication year, title, country or region, sociodemographic index (SDI), World Health Organization (WHO) region, World Bank region, study setting, and geographic coordinates (longitude and latitude);

– Study design: study period, study design, study name, and follow-up duration (years);

– Sample population characteristics: type of study setting (urban, rural, or both), inclusion criteria, exclusion criteria, age, sample size, proportion of females, and number of events;

– Exposure and outcome: exposure measures (NDVI), outcome measures (*e.g.* hazard ratios, ORs), effect sizes, 95% confidence intervals (CIs), *P*-value, and covariates adjusted in models.

We used the Newcastle-Ottawa Scale and its three domains (selection, comparability, and exposure) to assess the quality of included articles [24] (Appendix S2 in the **Online Supplementary Document**). Scores of 0–3 were considered low, 4–6 were considered moderate, and 7–9 were considered high quality, with 9 being the maximum possible score [25]. Disagreements in any process were resolved by consulting a third reviewer (PS).

Given the different metrics used for greenness, we only included articles using NDVI as its proxy. For multiple estimates from different models in an article, we selected the estimate of the fully adjusted model or the one most comparable across studies. We only analysed studies with continuous NDVI measures to maintain consistency in exposure levels. For estimates reported at various buffer sizes, we only included the 500 m buffer; if unavailable, we used the closest buffer size. We assessed depression risk based on an NDVI increase of 0.1 units. For studies reporting quartiles, we transformed the OR per interquartile range to OR per 0.1 unit after calculating the difference between the upper and lower quartiles. We used a random-effects meta-analysis to pool the ORs for the association between greenness and depression. Where studies used different increments, we standardised estimates as:


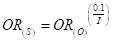


where *OR*_(_*_S_*_)_ represents the standardised effect estimates, *OR*_(_*_O_*_)_ the original effect estimates, and *I* the original NDVI increment.

We assessed the heterogeneity between studies using Cochran’s Q statistic (with *P* < 0.05 indicating significant heterogeneity) and the *I*^2^ statistic (with values ≥50% indicating substantial heterogeneity) [26]. We visually examined publication bias through funnel plots and tested it further by Egger’s test and Begg’s test (*P* < 0.05 indicating significant publication bias) when the number of included articles was ≥10 [27,28]. When publication bias was detected, we used the trim-and-fill method to adjust the effect estimate [29]. Additionally, we conducted a leave-one-out sensitivity analysis to assess the robustness of our results.

### Green space exposure

Among satellite-based methods, the NDVI is a widely used index in vegetation studies and urban green space extraction. It is defined as [30]:


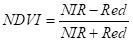


where *NIR* represents the amount of near-infrared light, and *Red* the amount of red light.

NDVI values range from −1.0 to 1.0, with higher NDVI values indicating greener and denser vegetation coverage on the surface [13]. The principle behind NDVI is that green vegetation reflects more near-infrared energy, while absorbing more red light in the electromagnetic spectrum. Negative values represent water features (*e.g.* rivers, coastal waters, or clouds), values close to zero indicate the absence of vegetation, and positive values signify the presence of green vegetation. To describe green space exposure at the city level, we chose the average NDVI in each city each year.

To assess green space exposure at the city level, we computed the average NDVI for each city annually. We used the Global Administrative Unit Layers from the Food and Agriculture Organization of the United Nations at the country and state administrative levels in 2015 as hierarchical units for spatial analysis of global green space exposure [31].

Additionally, we developed an adaptive threshold algorithm using WorldPop population data and land use/land cover data from the C3S Climate Data Store (2019) to delineate built-up areas for cities between 2001 and 2020. This method uses the WorldPop population data of the current year and the urbanisation rate data of each country to calculate the urban population of a given country. Then, populated areas are sequentially included as urban areas until the total population exceeds the urban population of the country. The resulting raster area defines the urban built-up area, including 30 887 cities across 226 countries.

We subsequently acquired annual average NDVI data for these 30 887 urban areas from 2001 to 2020 using Google Earth Engine. We derived the NDVI data from the MOD13A2 V6.1 product, providing gridded NDVI values at a resolution of 1 km × 1 km, selecting the maximum value within each 16 days [32,33].

### Green space expansion scenarios

There are many possible approaches to increasing the percentage of green space or population with access to nearby natural space. To compare the health benefits of alternative methods, we developed and applied three illustrative scenarios that achieve the same city-average increases in green space but distribute additions in different ways. In all scenarios, we restricted NDVI values from exceeding one, its maximum value.

1. Best potential scenario: we increased the NDVI of all cities within each country to the highest level observed in any city of that country.

2. Proportional increase scenario: we increased the NDVI of all cities within each country by 10%.

3. Uniform increase scenario: we increased the NDVI by 0.08 for all cities within each country.

Realistically, allocation decisions for adding green space are driven by a complex multitude of factors; these scenarios are meant to provide a conceptual understanding of how mental health benefits of alternative approaches may differ. All NDVI increases are assumed to come from expanding green space.

### Disease burden of depression

We quantified the disease burden of depression using DALYs, a standard metric from the GBD 2021 study. We extracted DALY rates for each five-year age category from 10–14 to ≥80 years. The time frame of analysis spanned from 2001 to 2020, using country-specific or subnational data where available.

### Statistical analysis

After estimating the preventable burden, we conducted further analyses to investigate its distribution, temporal trends, sex differences, and inequality patterns.

To ensure comparability across populations, we reported age-standardised DALY rates (ASDRs) per 100 000 population, using population weights from the GBD 2021 as the standard [34]. We applied the delta method to estimate the 95% uncertainty interval (UI) of ASDRs, treating the age-specific rates as log-normally distributed and calculating their standard deviations:



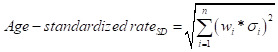



where *w_i_* is the standard population weight and *σ_i_* is the standard deviation of the age-specific rate for the age group *i*.

We described the distribution of ASDRs per 100 000 population for preventable depression due to green space expansion across sexes, different global regions (five SDI regions and six WHO regions), and 176 countries/territories from 2001 to 2020.

We estimated average annual percentage changes (AAPCs) by joinpoint regression to measure the average temporal trend in burden over 2001–20. We considered AAPC the annual change percentage transformed from the weighted average of the slope coefficients of the underlying joinpoint regression model from 2001 to 2020 [35]. When the AAPC and its lower limit of 95% CI were both positive, we considered ASDRs to be increasing. Conversely, we considered them to be decreasing when the AAPC and its upper limit of 95% CI were both negative. If the estimated CIs overlapped zero, the ASDRs were deemed to be stable.



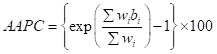



sWhere *b_i_* is the slope coefficient for the *i*th segment, with *i* indexing the segments in the desired range of years, and *w_i_* is the length of each segment in the range of years.

We evaluated the sex differences in the preventable burden by calculating the absolute differences in ASDRs (*i.e.* female ASDR − male ASDR) and relative ratios (*i.e.* female ASDR / male ASDR) for each country, region, and globally. We analysed the temporal trends in these sex-specific disparities using the AAPCs from 2001 to 2020 to examine how these differences evolved.

We adopted the slope index of inequality (SII) and concentration index, which are standard metrics of absolute and relative gradient inequality, to quantify the distributive inequality in the preventable burden of depression across countries [36]. We calculated the SII by regressing national ASDRs on an SDI-associated relative position scale, which we defined by the midpoint of the cumulative range of the population ranked by the SDI in this country. We addressed heteroskedasticity using a robust regression model. We calculated the concentration index by numerically integrating the area under the Lorenz concentration curve, which we fitted using the cumulative fraction of DALYs against the cumulative relative distribution of the population ranked by SDI. This index ranges from −1 (perfect inequality favouring the lowest SDI) to 1 (perfect inequality favouring the highest SDI). For DALYs of depression, a negative SII/concentration index indicated that lower SDI countries bear a higher preventable burden, and vice versa. A larger absolute value of the SII/concentration index indicated greater inequality. We used the bootstrap method to compute the 95% CIs of SII and the concentration index.

We conducted all descriptive, trend, sex-specific, and inequality analyses for the overall population, and for males and females separately. To better understand the burden and inequalities at the regional level, we stratified all analyses for five SDI regions (*i.e.* high, high-middle, middle, low-middle, and low) and six WHO regions (*i.e.* the African region (AFR), the Eastern Mediterranean region (EMR), the South-East Asia region (SEAR), the region of the Americas (AMR), the Western Pacific region (WPR), and the European region (EUR)) [37], from 2001 to 2020.

We reported ASDRs per 100 000 population along with 95% UIs, while presenting SII, concentration index, and AAPC with their respective 95% CIs. To account for uncertainty in the pooled ORs, green space exposure data, and depressive disorder DALYs, we conducted 1000 Monte Carlo simulations. We assumed pooled ORs and DALYs to follow a log-normal distribution, and we derived the 95% UIs for the preventable burden from the 2.5th and 97.5th percentiles of the simulations.

We used *R*, version 4.3.2 (R Core Team, Vienna, Austria) for all statistical analyses. All statistical tests were two-sided, and we considered *P*-values <0.05 statistically significant.

## RESULTS

### The global distribution of green space in 2020

Given the minimal variation in annual global NDVI distribution from 2001 to 2020, we used the year 2020 as a representative snapshot to reflect current green space exposure. Across 30 887 urban or built-up area sampling sites across 226 countries, each country has at least one sampling site (Figure S1 in the **Online Supplementary Document**). After excluding invalid values (≤0, indicating areas without vegetation cover), valid NDVI data were available for 30 837 sites. NDVI values ranged from 0.04 to 0.84, with most values concentrated in the range of 0.4–0.8. The global spatial distribution of green space showed significant regional variation. Higher NDVI values were observed in the AMR and the WPR, while lower values were prominent in the EMR. Notable intra-regional differences were also identified. For instance, London had an NDVI of 0.46, nearly double that of Paris (NDVI = 0.24), despite both cities being in Western Europe. Additionally, NDVI levels demonstrated a relatively uniform distribution across varying urbanisation rates and SDI levels, with no apparent correlation observed.

### The association between greenness and depression

We identified 2408 reviews through the umbrella review process. After removing 467 duplicates, we screened the titles and abstracts of 1941 records, followed by the full text of any remaining ones, with 104 finally meeting the inclusion criteria. From these reviews, we retrieved 1093 original articles; 26 investigated depression outcomes, with nine being included in the meta-analysis (Appendix S3, Table S3 in the **Online Supplementary Document**). The pooled OR from the meta-analysis indicated a significant protective effect of green space (measured as NDVI) against depression. Specifically, an increase in NDVI of 0.1 units was associated with a standardised OR of 0.92 (95% CI = 0.87, 0.97), corresponding to an 8% risk reduction in depression (Figure S4 in the **Online Supplementary Document**). Heterogeneity among the studies was substantial (*I*^2^ = 91%; *P* < 0.05), and no significant publication bias was detected based on Egger’s test (*P* = 0.12) and funnel plot (Figure S5 in the **Online Supplementary Document**). Sensitivity analyses confirmed the robustness of these findings (Figure S6 in the **Online Supplementary Document**).

### The preventable burden of depression under the best potential scenario

Under the best potential scenario, if a country only had one city with available green space exposure data, its estimated values for the preventable burden would be zero. Consequently, we primarily included results from 176 countries where the preventable burden was >0 and described the distribution, gender differences, and cross-country SDI-related inequality of preventable disease burden at global, regional, and national levels.

### The preventable burden and the temporal trends of depression from 2001 to 2020

The preventable ASDR of depression per 100 000 population due to green space expansion for both sexes increased from 93.601 (95% UI = 87.787, 99.415) in 2001 to 117.665 (95% UI = 110.608, 124.722) in 2020 globally, with an AAPC of 1.13% (95% CI = 0.52, 1.75) (**Table 1**). All six WHO regions experienced an increase in the preventable ASDR of depression for both sexes, except the EMR (AAPC = 0.43%; 95%CI = –0.37, 1.23) and the SEAR (AAPC = 0.83%; 95% CI = –0.06, 1.73), which remained relatively stable. The AFR exhibited the most substantial growth, whose ASDR per 100 000 population shifted from 84.363 (95% UI = 78.095, 90.630) in 2001 to 122.646 (95% UI = 113.419, 131.872) in 2020, with an AAPC of 1.78% (95% CI = 1.56, 1.99). Meanwhile, the AMR recorded the highest preventable ASDR per 100 000 population for both sexes in 2020 at 287.827 (95% UI = 252.668, 322.985). Accordingly, the WPR recorded the lowest preventable ASDR per 100 000 population in the same year, at 62.150 (95% UI = 54.088, 70.211). The preventable ASDR of depression was consistently higher for females than for males across all WHO regions. While we noted a similar trend pattern among females in six WHO regions, there were significant increases among males in all regions from 2001 to 2020.

**Table 1:** Estimates and AAPC of preventable ASDR of depression attributable to greenness exposure (2001–20)

			

	Female			Male			Both		
	2001*	2020*	**2001–20**†	2001*	2020*	**2001–20**†	2001*	2020*	**2001–20**†
**Global**	115.041 (107.883, 122.220)	144.985 (136.211, 153.759)	1.131% (0.521, 1.747)	72.170 (67.659, 76.681)	90.481 (85.030, 95.932)	1.153 (0.534, 1.776)	93.601 (87.787, 99.415)	117.665 (110.608, 124.722)	1.132% (0.521, 1.747)
**WHO region**									
AFR	98.568 (91.250, 105.585)	141.977 (131.113, 152.841)	1.722% (1.508, 1.937)	69.609 (64.318, 74.901)	101.800 (94.211, 109.389)	1.809 (1.590, 2.028)	84.363 (78.095, 90.630)	122.646 (113.419, 131.872)	1.776% (1.560, 1.992)
AMR	290.070 (255.260, 324.881)	371.962 (325.757, 418.166)	1.157% (0.414, 1.905)	166.650 (146.744, 186.556)	202.231 (178.247, 226.215)	0.968 (0.196, 1.747)	229.414 (201.632, 257.195)	287.827 (252.668, 322.985)	1.071% (0.327, 1.821)
EMR	141.528 (125.834, 157.222)	169.225 (151.591, 186.860)	0.376% (−0.429, 1.188)	87.026 (77.756, 96.296)	105.652 (94.978, 116.326)	0.514 (0.039, 0.991)	113.137 (100.843,125.432)	136.020 (122.068, 149.972)	0.427% (−0.367, 1.226)
EUR	101.539 (92.113, 110.965)	126.047 (111.501, 140.593)	0.750% (0.380, 1.122)	62.158 (56.625, 67.690)	74.771 (66.376, 83.167)	1.053 (0.036, 2.080)	82.382 (74.957, 89.807)	100.679 (89.396, 111.963)	0.657% (0.276, 1.040)
SEAR	89.660 (72.748, 106.571)	108.306 (89.373, 127.238)	0.705% (−0.187, 1.604)	62.905 (50.976, 74.834)	80.158 (65.543, 94.774)	0.967 (0.052, 1.890)	75.908 (61.577, 90.239)	94.286 (77.595, 110.977)	0.834% (−0.058, 1.734)
WPR	64.929 (56.039, 73.819)	74.759 (64.905, 84.612)	0.762% (0.366, 1.159)	42.621 (36.808, 48.434)	49.436 (43.185, 55.687)	0.733 (0.354, 1.113)	53.782 (46.443, 61.121)	62.150 (54.088, 70.211)	0.750% (0.363, 1.138)
**SDI region**									
High	182.192 (154.019, 210.366)	232.787 (192.011, 273.563)	1.246% (0.135, 2.370)	108.449 (92.299, 124.599)	133.561 (112.661, 154.462)	1.128 (0.034, 2.233)	144.988 (122.639, 167.338)	181.474 (151.515, 211.433)	1.159% (0.067, 2.264)
High-middle	72.195 (64.475, 79.916)	88.755 (78.726, 98.784)	1.126% (0.852, 1.401)	43.637 (38.853, 48.421)	51.777 (45.894, 57.659)	0.915 (0.633, 1.199)	58.043 (51.867, 64.220)	70.184 (62.294, 78.074)	1.024% (0.745, 1.303)
Middle	185.134 (165.372, 204.896)	251.744 (225.922, 277.566)	1.406% (0.703, 2.114)	114.425 (103.400, 125.450)	149.995 (136.750, 163.239)	1.317 (0.617, 2.021)	150.777 (135.323, 166.232)	201.142 (181.689, 220.595)	1.349% (0.652, 2.051)
Low-middle	101.265 (85.701, 116.829)	115.941 (100.137, 131.754)	0.362% (−0.313, 1.041)	69.049 (57.997, 80.100)	82.287 (70.081, 94.493)	0.630 (−0.072, 1.337)	84.392 (71.214, 97.570)	99.009 (84.883, 113.135)	0.494% (−0.207, 1.200)
Low	81.503 (75.321, 87.685)	120.973 (112.293, 129.653)	1.951% (1.238, 2.669)	58.556 (53.797, 63.314)	87.513 (80.960, 94.066)	2.015 (1.269, 2.767)	70.260 (64.826, 75.694)	104.804 (97.251, 112.357)	1.992% (1.265, 2.724)

AAPC – average annual percent change, AFR – African region, AMR – region of the Americas, ASDR – age-standardised DALY rate, DALY – disability-adjusted life year, EMR – Eastern Mediterranean region, EUR – European region, SDI – sociodemographic index, SEAR – South-East Asia region, WHO – World Health Organization, WPR – Western Pacific region

*Values presented as ASDR per 100 000 (95% uncertainty interval).

†Values presented as AAPC (95% confidence interval).

Among five SDI regions, all except the low-middle SDI region experienced significant increases for both sexes from 2001 to 2020, with AAPC values ranging from 1.02% (95% CI = 0.75, 1.30) to 1.99% (95% CI = 1.23, 2.72). The most significant increases in ASDR were observed in the low SDI region. In 2020, the middle SDI region (ASDR = 201.142; 95% UI = 181.689, 220.595) observed the highest preventable SDI for both sexes, and the high SDI region followed (ASDR = 181.474; 95% UI = 151.515, 211.433). Similar to the situation in the WHO region, the preventable ASDR of depression was consistently higher for females than males across all SDI regions (Figure S14 in the **Online Supplementary Document**). Additionally, the preventable ASDR for both sexes, females, and males demonstrated a similar trend pattern across six SDI regions from 2001 to 2020.

**Figure 2:**
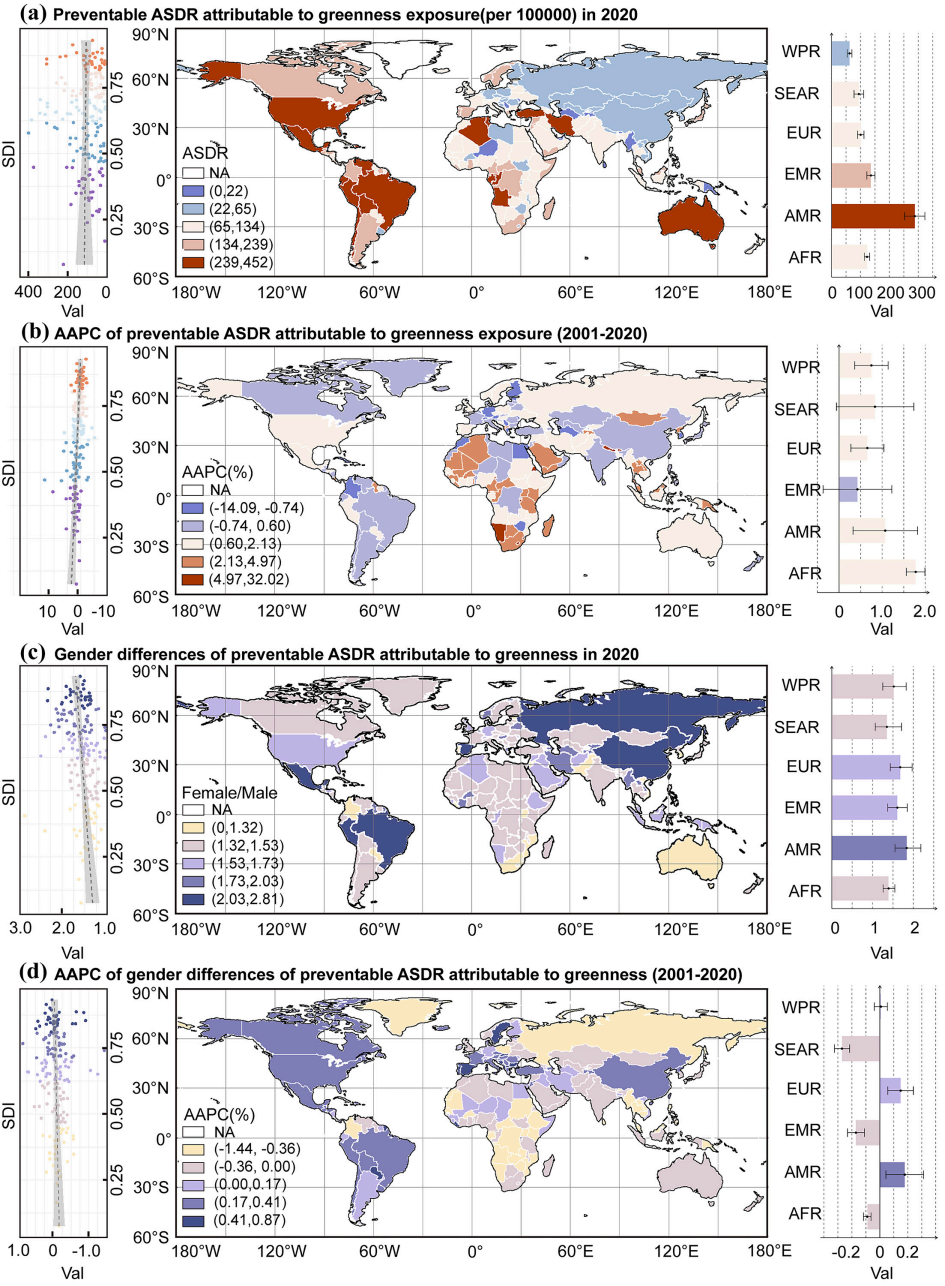
Global distribution of ASDR for preventable depression in 2020 and AAPC from 2001 to 2020. **Panel A.** Preventable ASDR attributable to greenness exposure (per 100 000) in 2020. **Panel B.** AAPC of preventable ASDR attributable to greenness exposure (2001–2020). **Panel C.** Gender difference of preventable ASDR attributable to greenness exposure in 2020. **Panel D.** AAPC of gender difference of preventable ASDR attributable to greenness (2001–2020). AAPC – average annual percent change, AFR – African region, AMR – region of the Americas, ASDR – age-standardised DALY rate, DALY – disability-adjusted life years, EMR – Eastern Mediterranean region, EUR – European region, SDI – sociodemographic index, SEAR – South-East Asia region, WPR – Western Pacific region.

From 2001 to 2020, the preventable ASDR of depression due to green space expansion varied across 176 countries and territories (**Figure 2**; Tables S5 and S6 in the **Online Supplementary Document**). In 2020, the top five countries that held the highest preventable ASDR per 100 000 population were Mexico (ASDR = 451.571; 95% UI = 132.155, 880.618), Congo (ASDR = 399.565; 95% UI = 112.241, 735.329), Gabon (ASDR = 383.911; 95% UI = 130.372, 726.074), Angola (ASDR = 373.639; 95% UI = 110.355, 738.803), and Algeria (ASDR = 341.368; 95% UI = 105.415, 674.943). Additionally, Anguilla (AAPC = 32.02%; 95% CI = 20.46, 44.68), Seychelles (AAPC = 29.37%; 95% CI = 4.80, 59.69), and Nepal (AAPC = 10.39%; 95% CI = 5.50, 15.48) exhibited the most substantial growth from 2001 to 2020.

### Gender differences in the preventable burden of depression from 2001 to 2020

We noted significant differences in the preventable burden of depression between sexes (**Figure 2**, **Table 2**; Table S8 in the **Online Supplementary Document**). Although the AAPCs of the two sexes showed a similar increasing trend, females exhibited a higher preventable ASDR compared to males in both 2001 and 2020. Specifically, the ASDR per 100 000 population for females was 115.041 (95% UI = 107.883, 122.220) in 2001 and 144.985 (95% UI = 136.211, 153.759) in 2020, compared to males at 72.179 (95% UI = 67.659, 76.681) in 2020 and 90.481 (95% UI = 85.030, 95.932) in 2020. We further separately explored the absolute differences (female − male), relative differences (female/male) and their temporal trends from 2001 to 2020. Overall, at the global and regional levels, both the absolute and relative differences indicated that females would achieve a greater preventable burden of depression due to green space expansion under the best potential scenario.

**Table 2:** Estimates and AAPC of preventable ASDR of depression attributable to greenness exposure (2001–20)

	Absolute difference (female − male)			Relative difference (female/male)		
	2001*	2020*	**2001–20**†	2001*	2020*	**2001-20**†
**Global**	42.901 (34.470, 51.143)	54.404 (44.671, 64.481)	1.101% (0.571, 1.633)	1.595 (1.459, 1.731)	1.599 (1.474, 1.734)	0.001% (−0.125, 0.127)
**WHO region**						
AFR	29.065 (20.090, 38.281)	39.913 (27.860, 53.439)	1.497% (1.292, 1.703)	1.417 (1.275, 1.573)	1.394 (1.260, 1.548)	−0.090% (−0.117, −0.063)
AMR	122.763 (83.732, 162.833)	169.129 (119.657, 224.511)	0.762% (0.264, 1.262)	1.735 (1.461, 2.054)	1.837 (1.550, 2.183)	0.178% (0.044, 0.313)
EMR	54.596 (37.508, 73.388)	64.139 (42.659, 84.550)	0.718% (−0.562, 2.015)	1.629 (1.407, 1.899)	1.608 (1.372, 1.852)	−0.171% (−0.231, −0.110)
EUR	39.350 (28.410, 51.157)	50.856 (34.680, 67.816)	1.041% (0.715, 1.368)	1.633 (1.435, 1.866)	1.677 (1.431, 1.970)	0.149% (0.057, 0.240)
SEAR	26.328 (7.496, 47.022)	27.956 (5.879, 51.828)	0.207% (−0.806, 1.230)	1.416 (1.106, 1.840)	1.351 (1.065, 1.709)	−0.271% (−0.325, −0.216)
WPR	21.943 (11.880, 33.916)	25.654 (13.525, 37.647)	1.041% (0.249, 1.838)	1.514 (1.255, 1.874)	1.518 (1.250, 1.826)	0.007% (−0.040, 0.054)
**SDI region**						
High	73.951 (42.121, 110.561)	99.534 (57.028, 149.022)	1.385% (0.156, 2.630)	1.691 (1.364, 2.124)	1.748 (1.388, 2.188)	0.086% (0.019, 0.153)
High-middle	28.444 (20.279, 38.068)	36.928 (25.212, 48.774)	1.459% (1.171, 1.748)	1.653 (1.441, 1.910)	1.716 (1.459, 2.019)	0.166% (0.037, 0.295)
Middle	70.939 (49.158, 93.961)	101.078 (74.718, 129.508)	1.075% (0.660, 1.493)	1.621 (1.401, 1.866)	1.677 (1.478, 1.910)	0.107% (0.041, 0.172)
Low-middle	32.247 (13.674, 51.043)	33.882 (15.368, 53.119)	−0.220% (−0.945, 0.510)	1.468 (1.181, 1.810)	1.410 (1.173, 1.714)	−0.227% (−0.363, −0.091)
Low	23.029 (15.546, 30.448)	33.583 (22.731, 44.665)	1.797% (1.137, 2.463)	1.395 (1.255, 1.550)	1.384 (1.246, 1.534)	−0.060% (−0.092, −0.028)

AAPC – average annual percent change, AFR – African region, AMR – region of the Americas, ASDR – age-standardised DALY rate, DALY – disability-adjusted life year, EMR – Eastern Mediterranean region, EUR – European region, SDI – sociodemographic index, SEAR – South-East Asia region, WHO – World Health Organization, WPR – Western Pacific region

*Values presented as ASDR per 100 000 (95% confidence interval).

†Values presented as AAPC (95% confidence interval).

At the global level, the absolute difference demonstrated a modest rise from 42.901 (95% CI = 34.470, 51.143) in 2001 to 54.404 (95% CI = 44.671, 64.481) in 2020, with an AAPC of 1.101% (95% CI = 0.571, 1.633). Meanwhile, the relative differences indicated an even trend (AAPC = 0.00%; 95% CI = –0.13, 0.13).

Among the six WHO regions, all exhibited a moderate upward trend from 2001 to 2020, except for EMR and SEAR. For instance, in the AMR, absolute differences increased from 122.763 (95% CI = 83.732, 162.833) in 2001 to 169.129 (95% CI = 119.657, 224.511) in 2020, which marked the highest value among all regions in 2020 (AAPC = 0.76; 95% CI = 0.26, 1.26). In contrast, EMR and SEAR showed relatively stable trends, with minimal changes over the same period. At the same time, the relative differences showed varying patterns across regions from 2001 to 2020. Notably, AMR and EUR exhibited a slight upward trend. The AMR experienced the most pronounced increase, with relative differences shifting from 1.735 (95% CI = 1.461, 2.054) in 2001 to 1.837 (95% CI = 1.550, 2.183) in 2020, accompanied by an AAPC value of 0.18 (95% CI = 0.04, 0.31). Meanwhile, AFR and SEAR displayed a decline in relative differences, whereas WPR remained relatively stable over the same period.

All five SDI regions showed a rising trend in absolute differences from 2001 to 2020, except for the low-middle SDI region, which remained relatively stable (AAPC = −0.22%; 95% CI = −0.95, 0.51). Among them, the middle SDI region recorded the highest absolute difference in 2020, while the low SDI region experienced the largest rising trend from 2001 to 2020, with an AAPC value reaching 1.80% (95% CI = 1.14, 2.46). In terms of relative differences, the high SDI, high-middle SDI, and middle SDI regions experienced an increase in disparities. The high SDI region displayed the highest relative difference (AAPC = 1.748%; 95% CI = 1.388, 2.188) in 2020, and the high-middle SDI region showed the comparatively largest increase, with relative difference rising from 1.653 (95% CI = 1.441, 1.910) in 2001 to 1.716 (95% CI = 1.459, 2.019) in 2020 (AAPC = 0.17%; 95% CI = 0.04, 0.30). Conversely, the low-middle and low SDI regions saw a slight reduction in relative disparities; the low-middle SDI region exhibited a marginally greater decline (AAPC = −0.23%; 95% CI = −0.36, −0.09).

The countries that led in terms of absolute difference were Mexico, Brazil, Turkey, USA, and Chile in 2020, with values varying from 160.100 (95% CI = −136.555, 690.092) to 279.746 (95% CI = −349.140, 1286.237). Regarding the relative difference, the highest countries in 2020 were Gambia, Paraguay, Liberia, Albania, and Slovenia, with estimated values ranging from 2.313 (95% CI = 0.772, 7.590) to 2.812 (95% CI = 0.853, 9.108). Additionally, among all countries and territories in 2020, only the Lao People’s Democratic Republic observed a negative absolute difference (absolute difference = −4.060; 95% CI = −93.041, 81.814) and a relative difference below one (relative difference = 0.945; 95% CI = 0.305, 3.014).

### Cross-country health inequalities of the preventable burden of depression in 2001 and 2020

Globally, compared to the concentration index in 2001 of 0.160 (95% CI = 0.088, 0.232), relative SDI-related inequalities in the preventable ASDR of depression due to green space expansion across 176 countries and territories narrowed in 2020, with a value of 0.051 (95% CI = −0.021, 0.123). As indicated by the SII, no significant absolute inequality was observed in both 2001 and 2020 (**Table 3**; Figure S11 in the **Online Supplementary Document**).

**Table 3:** Estimates and AAPC of preventable ASDR of depression attributable to greenness exposure (2001–20)

	Female				Male				Both			
	**2001**		**2020**		**2001**		**2020**		**2001**		**2020**	
	**Concentration index (95% CI)**	**SII (95% CI)**	**Concentration index (95% CI)**	**SII (95% CI)**	**Concentration index (95% CI)**	**SII (95% CI)**	**Concentration index (95% CI)**	**SII (95% CI)**	**Concentration index (95% CI)**	**SII (95% CI)**	**Concentration index (95% CI)**	**SII (95% CI)**
**Globa**l	0.173 (0.098, 0.248)	10.922 (−18.546, 40.389)	0.070 (−0.006, 0.146)	−18.006 (−64.933, 28.912)	0.135 (0.068, 0.203)	7.357 (−15.164, 29.878)	0.023 (−0.044, 0.089)	−22.644 (−52.124, 6.836)	0.160 (0.088, 0.232)	6.549 (−17.567, 30.644)	0.051 (−0.021, 0.123)	−21.205 (−59.244, 16.833)
**WHO region**												
AFR	0.13 (0.036, 0.233)	17.927 (−33.801, 69.655)	0.182 (0.076, 0.287)	30.347 (−43.032, 103.726)	0.109 (0.006, 0.212)	16.180 (−19.199, 51.588)	0.170 (0.066, 0.273)	31.192 (−19.104, 81.489)	0.124 (0.024, 0.224)	16.677 (−29.401, 62.755)	0.176 (0.072, 0.280)	29.845 (−32.047, 91.737)
AMR	0.007 (−0.051, 0.065)	−16.344 (−153.647, 120.960)	0.019 (−0.054, 0.093)	−77.167 (−250.559, 96.224)	0.002 (−0.051, 0.055)	−12.071 (−92.530, 68.388)	0.020 (−0.039, 0.080)	−48.519 (−144.884, 47.847)	−0.001 (−0.057, 0.056)	−16.868 (−126.224, 92.487)	0.019 (−0.049, 0.086)	−64.685 (−199.619, 70.249)
EMR	0.201 (0.041, 0.362)	10.802 (−104.412, 126.017)	0.124 (−0.038, 0.286)	−52.802 (−183.345, 79.180)	0.160 (0.003, 0.316)	1.025 (−72.880, 74.930)	0.065 (−0.088, 0.218)	−45.012 (−129.838, 39.813)	0.182 (0.022, 0.341)	3.909 (−89.829, 97.646)	0.095 (−0.066, 0.255)	−51.744 (−159.760, 57.531)
EUR	−0.165 (−0.322, 0.001)	27.773 (−13.820, 69.365)	−0.213 (−0.370, −0.056)	2.934 (−50.553, 56.412)	−0.136 (−0.280, 0.008)	7.999 (−27.035, 43.033)	−0.182 (−0.324, −0.039)	5.749 (−24.989, 36.487)	−0.154 (−0.310, 0.001)	5.702 (−43.478, 54.833)	−0.200 (−0.350, −0.051)	3.458 (−39.167, 46.083)
SEAR	0.005 (−0.150, 0.160)	36.812 (−21.098, 94.721)	0.073 (−0.048, 0.195)	43.278 (−27.042, 113.598)	0.028 (−0.121, 0.177)	27.268 (−11.787, 66.323)	0.103 −0.008, 0.214)	39.315 (−7.949, 86.578)	0.015 (−0.136, 0.166)	32.423 (−15.596, 80.442)	0.085 (−0.031, 0.201)	41.786 (−17.385, 100.957)
WPR	0.637 (0.219, 1.054)	35.520 (−12.731, 83.772)	0.404 (−0.076, 0.885)	39.919 (−24.859, 104.696)	0.711 (0.270, 1.151)	21.355 (−9.510, 52.181)	0.411 (−0.161, 0.982)	24.647 (−18.627, 67.921)	0.669 (0.239, 1.100)	28.409 (−11.537, 68.354)	0.404 (−0.112, 0.919)	31.779 (−18.188, 81.746)
**SDI region**												
High	0.080 (−0.056, 0.216)	49.033 (−20.115, 118.182)	−0.012 (−0.155, 0.131)	47.914 (−34.995, 130.824)	0.097 (−0.033, 0.226)	26.738 (−15.692, 69.169)	0.002 (−0.137, 0.140)	23.631 (−29.052, 76.314)	0.087 (−0.046, 0.219)	37.306 (−18.373, 92.984)	−0.005 (−0.147, 0.137)	35.692 (−32.084, 103.467)
High-middle	0.401 (0.077, 0.726)	−75.509 (−310.518, 159.501)	−0.013 (−0.329, 0.302)	−251.854 (−438.973, −64.375)	0.437 (0.152, 0.721)	−47.035 (−180.808, 86.738)	0.022 (−0.287, 0.332)	−145.625 (−248.357, −42.893)	0.425 (0.115, 0.735)	−59.547 (−244.216, 125.112)	0.005 (−0.307, 0.317)	−197.153 (−341.108, −53.198)
Middle	0.107 (−0.035, 0.249)	−37.457 (−133.638, 58.723)	0.078 (−0.063, 0.220)	−13.051 (−140.982, 114.879)	0.090 (−0.033, 0.213)	−35.507 (−96.836, 25.822)	0.082 (−0.033, 0.196)	−20.286 (−100.284, 59.711)	0.104 (−0.031, 0.239)	−36.215 (−114.611, 42.182)	0.080 (−0.051, 0.211)	−17.161 (−120.280, 85.959)
Low-middle	0.128 (0.035, 0.221)	42.552 (−11.724, 96.829)	0.038 (−0.054, 0.130)	20.476 (−56.663, 97.614)	0.116 (0.029, 0.203)	24.544 (−9.739, 58.828)	0.052 (−0.028, 0.133)	9.472 (−36.553, 55.498)	0.126 (0.035, 0.217)	33.482 (−11.047, 78.010)	0.040 (−0.047, 0.128)	14.582 (−49.363, 78.527)
Low	0.074 (−0.065, 0.212)	−0.456 (−61.574, 60.661)	0.146 (0.006, 0.287)	22.839 (−69.678, 115.355)	0.056 (−0.092, 0.203)	−1.383 (−46.202, 43.437)	0.137 (−0.003, 0.277)	12.007 (−43.340, 67.354)	0.066 (−0.076, 0.207)	−0.673 (−53.130, 51.783)	0.142 (0.001, 0.282)	17.570 (−57.929, 53.069)

AAPC – average annual percent change, AFR – African region, AMR – region of the Americas, ASDR – age-standardised DALY rate, CI – confidence interval, DALY – disability-adjusted life year, EMR – Eastern Mediterranean region, EUR – European region, SDI – sociodemographic index, SEAR – South-East Asia region, SII – slope index of inequality, WHO – World Health Organization, WPR – Western Pacific region

Within the six WHO regions, AFR demonstrated a disproportionately higher preventable burden among countries with higher SDI in both 2001 and 2020. The concentration index increased from 0.124 (95% CI = 0.024, 0.224) in 2001 to 0.176 (95% CI = 0.072, 0.280) in 2020, highlighting a growing relative inequality. Conversely, both EMR and WPR exhibited improvements in relative inequalities over the same period. In EMR, the concentration index decreased from 0.182 (95% CI = 0.022, 0.341) in 2001 to 0.095 (95% CI = −0.066, 0.255) in 2020, while in WPR, it declined from 0.669 (95% CI = 0.239, 1.100) to 0.404 (95% CI = −0.112, 0.919). In contrast, the EUR experienced a worsening relative inequality among nations with lower SDI. The concentration index shifted from –0.154 (95% CI = −0.310, 0.001) in 2001 to –0.200 (95% CI = −0.350, −0.051) in 2020, indicating an increasing concentration of the preventable burden in countries with lower SDI.

Among the five SDI regions, the high-middle SDI region exhibited an unexpected inequality pattern; absolute inequality was widening, while relative inequality was narrowing. Absolute inequality, as measured by the SII, was −59.547 (95% CI = −244.216, 125.112) in 2001 and −197.153 (95% CI = −341.108, −53.198) in 2020, indicating that the preventable burden of depression became more concentrated in lower SDI countries. However, the concentration index, reflecting relative inequality, decreased from 0.425 (95% CI = 0.115, 0.735) in 2001 and 0.005 (95% CI = −0.307, 0.317) in 2020, suggesting that the relative inequality narrowed over time. From 2001 to 2020, the low-middle SDI region exhibited a narrowing relative inequality, with the concentration index shifting from 0.126 (95% CI = 0.035, 0.217) to 0.040 (95% CI = −0.047, 0.128). Conversely, the low SDI region showed a widening relative inequality, with the concentration index increasing from 0.066 (95% CI = −0.076, 0.207) in 2001 to 0.142 (95% CI = 0.001, 0.282) in 2020. When stratified by sex, the results remained basically consistent across subgroups, indicating similar inequality patterns among both males and females.

The results of burden estimates were all calculated under the best potential scenario (Figures S7–10, S12, and S13, Tables S5–8 in the **Online Supplementary Document**).

## DISCUSSION

This is the first global quantification of the burden of depression that is potentially preventable through urban greening, using a scenario-based HIA across over 30 000 cities in 226 countries. By integrating high-resolution NDVI data, a robust meta-analysis, and GBD estimates, we demonstrate that urban green space has a measurable and potentially transformative role in reducing depression burden at the population scale.

We used scenario-based modelling of greening interventions, which allows for the exploration of both idealised and incremental strategies across diverse geopolitical, socioeconomic, and climatic contexts. This framework provides urban planners and public health policymakers with a flexible tool to evaluate the health co-benefits of greening, tailored to local constraints and opportunities.

### Unequal potential for health gains through greening

Despite the global increase in the preventable burden of depression from 2001 to 2020, our findings suggest that there remains a marked inequality in the distribution of this potential burden reduction. Middle-SDI countries experienced the highest age-standardised burden of depression preventable by green space expansion in 2020, while low-SDI countries saw the fastest growth. These trends suggest that countries in earlier stages of urban development have substantial unrealised potential for mental health improvement through greening.

The AMR recorded the highest preventable burden, likely reflecting high underlying depression prevalence combined with urban structural inequalities in green space access [38,39]. In contrast, the AFR showed the most rapid increase in preventable burden, possibly due to escalating urbanisation without proportional investment in green infrastructure [40,41]. Notably, Mexico, Congo, and Gabon ranked highest in preventable burden, while Anguilla, Seychelles, and Nepal showed the fastest growth. These patterns point to a convergence of mental health need and urban transformation, particularly in regions with emerging or transitional urban systems [42].

Environmental constraints also shape the feasibility and impact of greening. For instance, arid climates in the EMR may limit greening potential [43–45], while governance and land use challenges in the SEAR may constrain implementation [46]. These findings highlight the importance of integrating environmental realities with urban health planning.

At a global level, relative inequality in preventable burden narrowed, suggesting some diffusion of greening potential. However, absolute disparities remain wide, and some regions, such as the AFR, experienced increasing inequality. The high-middle SDI group exhibited a complex pattern, with relative inequality decreasing but absolute differences growing. This may reflect heterogeneity within the group, where lower-income countries face greater barriers to greening despite shared urbanisation trajectories [47].

### Sex disparities in green space-related mental health benefits

Our findings also show consistent and widening sex differences in the preventable burden of depression, with females experiencing greater potential benefits from green space expansion. Globally, the absolute difference in preventable ASDR between women and men increased from 42.9 in 2001 to 54.4 in 2020 per 100 000. This aligns with prior evidence that women are more vulnerable to depression [48,49], but also possibly more responsive to the restorative effects of nature exposure [50].

Mexico and Brazil exhibited the largest absolute gender gaps, while Gambia and Paraguay showed the highest relative disparities, suggesting both epidemiological and sociocultural drivers. These findings underscore the need for gender-sensitive greening strategies, especially in regions where high depression burden and limited green infrastructure co-occur [51].

Importantly, the observed disparities may not solely reflect biological susceptibility, but may also indicate gender-related differences in green space access, use, and perception [52,53]. For example, concerns about safety, caregiving responsibilities, and cultural norms may influence how women engage with urban nature [54,55]. Future research should explore these behavioural and contextual factors to guide the design of inclusive and equitable green environments.

### Limitations and future directions

This study has several limitations. First, the NDVI provides a standardised measure of vegetation cover, but does not capture other critical attributes of green space such as accessibility, safety, biodiversity, or social and cultural relevance, all of which may mediate its mental health benefits [56,57]. Future work should incorporate richer metrics, including perceived greenness and green space quality, especially in urban health research at national or sub-national scales [58]. Second, while our meta-analysis supports a protective association between green space and depression, causal inference remains limited. Longitudinal studies and natural experiments are needed to disentangle causality from confounding and to identify dose-response relationships across populations and environments. Third, the ‘best potential’ scenario we used represents an idealised upper bound. While useful for benchmarking, it does not account for political, cultural, or logistical constraints. Real-world implementation will require adaptation to local land use patterns, policy priorities, and resource availability. Finally, we focussed on depression alone, which represents only part of the mental health benefits potentially attributable to green space. Evidence suggests green space may also reduce anxiety, stress, and cognitive decline. Future HIAs should adopt a broader lens to capture these multifaceted effects.

### Broader implications for global health and urban sustainability

Our findings carry important implications for urban planning and global health. As mental disorders rise and cities expand, urban green space represents a unique, non-clinical intervention with both preventive and therapeutic potential. It can be embedded into housing, transportation, and climate adaptation strategies, integrating mental health into the broader sustainability agenda.

However, realising these benefits will require intentional investment in equitable greening, particularly in settings where infrastructure is limited and mental health needs are high. This includes not only expanding green space quantity, but also improving its quality, accessibility, and cultural relevance. Interdisciplinary collaboration, spanning public health, urban design, social science, and environmental policy, is essential. Greening is not a panacea, but when viewed through a systems lens, it offers powerful co-benefits for climate resilience, biodiversity, and human well-being.

## CONCLUSIONS

We provide the first comprehensive global assessment of the preventable burden of depression attributable to green space expansion, highlighting its promise as a public health intervention to promote mental well-being and address disparities. Using high-resolution NDVI data and a scenario-based framework, the findings show significant gender and regional inequalities in the potential benefits of green space, highlighting the need for targeted green space interventions to improve equity and mental health outcomes. Policymakers and urban planners should prioritise the integration of equitable green space into sustainable urban development, particularly in underserved regions, to address the global burden of depression and promote mental health equity.

## Additional material


Online Supplementary Document
