# Supplementary material for: The global, regional, and national preventable burden of depression attributable to greenness and inequalities: a scenario-based health impact analysis
Source: J Glob Health. 2025 Oct 3;15:04280. doi: 10.7189/jogh.15.04280 (PMC12491903; doi:10.7189/jogh.15.04280)
Supplement: Online Supplementary Document [file jogh-15-04280-s001.pdf]

Supplement to: Wu J, Di W, Ruan J, Li S, Ying J, Zhou J, Rudan I, Song P. The global, regional and national preventable burden of depression attributable to greenness and inequalities: a scenario-based health impact analysis. J Glob Health. 2025;15:04280.

Supplementary material

Wu et al. The global, regional and national preventable burden of depression attributable to greenness and inequalities: a scenario-based health impact analysis.

This supplementary material has been provided by the authors to give readers additional information about their work.

Contents

Appendix 1. Search strategy of umbrella review used in each database. 4
Appendix 2. The Newcastle Ottawa Scale (NOS) versions for different study designs. 5
Appendix 3. Studies retained for analysis (N = 9). 7
Appendix 4. Explanation of country included across three greenspace expansion scenarios. 8
Appendix 5. Supplementary tables and figures 9
Table S1. Top 20 cities with the highest and lowest average NDVI values in 2020. 9
Table S2. Basic data for 176 countries in 2020. 10
Table S3. A summary of the studies that met the criteria of the systematic review on the associations between greenness exposure (measured by NDVI) and depression. 19
Table S4. The results of Newcastle Ottawa Scale (NOS) for quality assessment of all include articles in meta-analysis. 20
Table S5. The PAF and ASDR for both sexes under three greenspace expansion scenarios in 176 countries in 2020. 21
Table S6. ASDR for males and females under three greenspace expansion scenarios in 176 countries in 2020. .36
Table S7. Gender differences of preventable age-standardised DALY rates of depression attributable to greenness exposure in 2020 under the proportional increase scenario and uniform increase scenario at the global and regional levels. 51
Table S8. Gender differences of preventable age-standardised DALY rates of depression attributable to greenness exposure from 2001 to 2020 under the three scenarios at the national level. 53
Figure S1. NDVI sampling and analysis of 30,887 cities around the world in 2020. 76
Figure S2. Changes in NDVI Values for the Five Most Populous Cities from 2001 to 2020. 77
Figure S3. PRISMA flow chart of the study selection and exclusion. 78
Figure S4. Odd ratio (OR) included in the study and the relationship between city-level NDVI lift and PAF.....79
Figure S5. Funnel plot in the pooled effect of greenness exposure on depression. 80
Figure S6. Leave-one-out sensitivity analysis of the influence of a single study on the pooled effect of greenness exposure on depression. 81
Figure S7. Global Distribution of ASDR for preventable ASDR of depressive disorders attributable to greenness exposure in 2020 and AAPC from 2001 to 2020 in proportional increase scenario. 82
Figure S8. Global Distribution of sex difference of ASDR for preventable ASDR of depressive disorders attributable to greenness exposure in 2020 and AAPC from 2001 to 2020 in proportional increase scenario. 83
Figure S9. Global Distribution of ASDR for preventable ASDR of depressive disorders attributable to greenness exposure in 2020 and AAPC from 2001 to 2020 in uniform increase scenario. 84
Figure S10. Global Distribution of sex difference of ASDR for preventable ASDR of depressive disorders attributable to greenness exposure in 2020 and AAPC from 2001 to 2020 in uniform increase scenario. 85
Figure S11. Absolute and relative cross-country inequality in preventable ASDR of depressive disorders attributable to greenness expansion in 2001 and 2020, with the temporal trend of inequality in preventable ASDR in Guinea from 2001 to 2020, under the best potential scenario. 86
Figure S12. Absolute and relative cross-country inequality in preventable ASDR of depressive disorders attributable to greenness expansion in 2001 and 2020 under the proportional increase scenario. 87

Figure S13. Absolute and relative cross-country inequality in preventable ASDR of depressive disorders attributable to greenness expansion in 2001 and 2020 under the uniform increase scenario. 88
Figure S14. World average ASDR and ASDR for countries in different SDI phases from 2001 to 2020 under the bset potential scenario. 89

Appendix 1. Search strategy of umbrella review used in each database.

Database	Search terms	
**Pubmed**	#1	Search ("Green space" [All Fields] OR Greenspace [All Fields] OR Greenness [All Fields] OR Greenery [All Fields] OR "Normalized Difference Vegetation Index" [All Fields] OR "Soil Adjusted Vegetation Index" [All Fields] OR "Enhanced Vegetation Index" [All Fields] OR Vegetation [All Fields] OR "Leaf area index"[All Fields])
	#2	Search ("systematic review" [All Fields] OR meta-analysis [All Fields])
	#3	Search (#1 AND #2)
**Embase**	#1	green space'/exp OR 'green space' OR 'greenspace'/exp OR greenspace OR 'greenness'/exp OR greenness OR greenery OR 'normalized difference vegetation index'/exp OR 'normalized difference vegetation index' OR 'soil adjusted vegetation index' OR 'enhanced vegetation index'/exp OR 'enhanced vegetation index' OR 'vegetation'/exp OR vegetation OR 'leaf area index'/exp OR 'leaf area index'
	#2	systematic review'/exp OR 'systematic review' OR 'meta-analysis'/exp OR 'meta-analysis'
	#3	#1 AND #2
**Web of science**	#1	TS=("Green space" OR Greenspace OR Greenness OR Greenery OR "Normalized Difference Vegetation Index" OR "Soil Adjusted Vegetation Index" OR "Enhanced Vegetation Index" OR Vegetation OR "Leaf area index"
	#2	TS=("systematic review" OR meta-analysis)
	#3	#1 AND #2
**MEDLINE**	#1	exp green space/ or exp greenspace/ or exp greenness/ or exp normalized difference vegetation index/ or exp enhanced vegetation index/ or exp vegetation/ or exp leaf area index/ or (green space or greenspace or greenness or greenery or normalized difference vegetation index or soil adjusted vegetation index or enhanced vegetation index or vegetation or leaf area index).mp
	#2	exp systematic review/ or exp meta-analysis/ or (systematic review or meta-analysis).mp
	#3	1 and 2

Number of records returned: 2408

Access Date: 16 July 2023

Appendix 2. The Newcastle Ottawa Scale (NOS) versions for different study designs.

Study	Newcastle Ottawa Scale (NOS)
**Cohort**	**SELECTION**<br>**1) Representativeness of the Exposed Cohort**<br>a) truly representative of the average characteristic in the community*<br>b) somewhat representative of the average characteristic in the community*<br>c) selected group of users eg nurses, volunteers<br>d) no description of the derivation of the cohort<br><br>**2) Selection of the Non-Exposed Cohort**<br>a) drawn from the same community as the exposed cohort*<br>b) drawn from a different source<br>c) no description of the derivation of the non exposed cohort<br>**3) Ascertainment of Exposure**<br>a) secure record (eg surgical records)*<br>b) structured interview*<br>c) written self report<br>d) no description<br>**4) Demonstration That Outcome of Interest Was Not Present at Start of Study**<br>a) yes*<br>b) no<br>**COMPARABILITY**<br>**1) Comparability of Cohorts on the Basis of the Design or Analysis**<br>a) study controls for age*<br>b) study controls for any additional factor*<br>**OUTCOME**<br>**1) Assessment of Outcome**<br>a) independent blind assessment*<br>b) record linkage*<br>c) self report<br>d) no description<br>**2) Was Follow-Up Long Enough for Outcomes to Occur**<br>a) yes*<br>b) no<br>**3) Adequacy of Follow Up of Cohorts**<br>a) complete follow up - all subjects accounted for*<br>b) subjects lost to follow up unlikely to introduce bias - small number lost > 75% follow up, or description provided of those lost)*<br>c) follow up rate < 75% (select an adequate %) and no description of those lost<br>d) no statement

Study	Newcastle Ottawa Scale (NOS)
**cross-sectional**	**SELECTION (Maximum 5 stars)**<br>**1) Representativeness of the Exposed Cohort**<br>a) truly representative of the average characteristic in the community*<br>b) somewhat representative of the average characteristic in the community*<br>c) selected group of users eg nurses, volunteers<br>d) no description of the derivation of the cohort<br>**2) Sample size**<br>a) >1000*<br>b) not justified<br>**3) Non-respondents**<br>a) comparability between respondents and non-respondents'characteristics is established, and the response rate is satisfactory (> 75%). *<br>b) the response rate is unsatisfactory, or the comparability between respondents and non-respondents is unsatisfactory.<br>c) no description of the response rate or the characteristics of the responders and the non-responders.<br>**4) Ascertainment of the adversity exposure (risk factor)**<br>a) validated measurement tool **<br>b) non-validated measurement tool, but the tool is available or described *<br>c) no description of the measurement tool<br>**Comparability (Maximum 2 stars)**<br>**5) Control for important Confounding factor: The subjects in different health outcome groups are comparable, based on the study design or analysis.**<br>a) the study controls for age. *<br>b) the study control for any additional factor.<br>**Outcome (Maximum 2 stars)**<br>**6) Assessment of the health outcome**<br>a) independent blind assessment *<br>b) record linkage *<br>c) self-report<br>d) no description<br>**7) Statistical test**<br>a) the statistical test used to analyze the data is clearly described and appropriate, and the measurement of the association is presented, including confidence intervals and the probability level (p value). *<br>b) the statistical test is not appropriate, not described, or incomplete.

***Notes**: * 1 point.*

Appendix 3. Studies retained for analysis (N = 9).

ID	REF
1	Banay RF, James P, Hart JE, et al. Greenness and Depression Incidence among Older Women. *Environ Health Perspect*. 2019;127(2):27001. doi:10.1289/EHP1229
2	Bezold CP, Banay RF, Coull BA, et al. The Association Between Natural Environments and Depressive Symptoms in Adolescents Living in the United States. *J Adolesc Health*. 2018;62(4):488-495. doi:10.1016/j.jadohealth.2017.10.008
3	Brown SC, Perrino T, Lombard J, et al. Health Disparities in the Relationship of Neighborhood Greenness to Mental Health Outcomes in 249,405 U.S. Medicare Beneficiaries. *Int J Environ Res Public Health.* 2018;15(3):430. Published 2018 Mar 1. doi:10.3390/ijerph15030430
4	Dzhambov AM, Hartig T, Tilov B, Atanasova V, Makakova DR, Dimitrova DD. Residential greenspace is associated with mental health via intertwined capacity-building and capacity-restoring pathways. *Environ Res*. 2019;178:108708. doi:10.1016/j.envres.2019.108708
5	Gascon M, Sánchez-Benavides G, Dadvand P, et al. Long-term exposure to residential green and blue spaces and anxiety and depression in adults: A cross-sectional study. *Environ Res*. 2018;162:231-239. doi:10.1016/j.envres.2018.01.012
6	Gonzales-Inca C, Pentti J, Stenholm S, Suominen S, Vahtera J, Käyhkö N. Residential greenness and risks of depression: Longitudinal associations with different greenness indicators and spatial scales in a Finnish population cohort. *Health Place.* 2022;74:102760. doi:10.1016/j.healthplace.2022.102760
7	Helbich M, Yao Y, Liu Y, Zhang J, Liu P, Wang R. Using deep learning to examine street view green and blue spaces and their associations with geriatric depression in Beijing, China. *Environ Int*. 2019;126:107-117. doi:10.1016/j.envint.2019.02.013
8	Lõhmus M, Stenfors CUD, Lind T, Lauber A, Georgelis A. Mental Health, Greenness, and Nature Related Behaviors in the Adult Population of Stockholm County during COVID-19-Related Restrictions. *Int J Environ Res Public Health*. 2021;18(6):3303. Published 2021 Mar 23. doi:10.3390/ijerph18063303
9	Song H, Lane KJ, Kim H, et al. Association between Urban Greenness and Depressive Symptoms: Evaluation of Greenness Using Various Indicators. *Int J Environ Res Public Health.* 2019;16(2):173. Published 2019 Jan 9. doi:10.3390/ijerph16020173

Appendix 4. Explanation of country included across three greenspace expansion scenarios.

In this study, we assessed the preventable burden of depression attributable to greenspace expansion under three different scenarios. However, the number of countries with available data varied across scenarios due to differences in the greenspace expansion strategies and the estimation of the population attributable fraction (PAF). Specifically, under the **best potential scenario**, if a country had only one city with available greenspace exposure data, measured by the Normalized Difference Vegetation Index (NDVI), its estimated preventable burden would be zero. Consequently, the number of countries included in this scenario was the smallest, with only **176** countries having the preventable burden estimates greater than zero.

For the **proportional increase scenario** and **uniform increase scenario**, differences in greenspace expansion strategies led to variations in city-level odds ratio (OR) estimates, which in turn affected national PAF estimation. As a result, the number of countries with non-zero estimates differed from that in the **best potential scenario**. To ensure comparability of nation-level estimates across scenarios, we primarily focused on the countries with non-zero preventable burden values in the **best potential scenario** and presented their corresponding estimates under the **proportional increase scenario** and **uniform increase scenario**. This approach allows for a more consistent comparison of preventable burden distributions and gender disparities at the national level.

Appendix 5. Supplementary tables and figures

Table S1. Top 20 cities with the highest and lowest average NDVI values in 2020.

	Top 20 cities				Lowest 20 cities		
	City	Location	NDVI		City	Location	NDVI
1	Tlapacoya	Mexico	0.834	1	Luderitz	Namibia	0.040
2	Tehuipango	Mexico	0.815	2	Keewatin	Canada	0.042
3	Halmahera Selatan	Indonesia	0.814	3	Tocopilla	Chile	0.046
4	San Rafael Pie de la Cuesta	Guatemala	0.813	4	Antofagasta	Chile	0.052
5	Amixtlan	Mexico	0.813	5	F'Derik	Mauritania	0.062
6	Ignacio Allende	Mexico	0.811	6	Ilo	Peru	0.067
7	Misakityoo	Japan	0.806	7	Tan-Tan	Morocco	0.070
8	Apaneca	El Salvador	0.806	8	Zahedan	Iran (Islamic Republic of)	0.072
9	Ixtepec	Mexico	0.804	9	Nouadhibou	Mauritania	0.074
10	Hermenegildo Galeana	Mexico	0.803	10	Al Wusta	Oman	0.074
11	Nova Brescia	Brazil	0.800	11	Zoueratt	Mauritania	0.077
12	Caxhuacan	Mexico	0.800	12	Al Mansura	Yemen	0.080
13	Victoria	Colombia	0.799	13	Thumrait	Oman	0.082
14	Hueytlalpan	Mexico	0.796	14	Ash Shaikh Outhman	Yemen	0.085
15	Barro Preto	Brazil	0.795	15	Fao	Iraq	0.086
16	Wujal	Australia	0.794	16	Iquique	Chile	0.086
17	Ikatatyoo	Japan	0.789	17	Alluheyah	Yemen	0.086
18	Guanaja	Honduras	0.788	18	Taghzout	Algeria	0.086
19	Coxcatlan	Mexico	0.788	19	Keetmanshoop Urban	Namibia	0.087
20	San Felipe Tepatlan	Mexico	0.787	20	El Oued	Algeria	0.088

Table S2. Basic data for 176 countries in 2020.

	Location	Population	Urbanization (%)	SDI	SDI Region	WHO Region	Sample
1	Afghanistan	30227744	26	0.33	Low	Eastern Mediterranean Region	155
2	Albania	2688665	62.1	0.704	Middle	European Region	1185
3	Algeria	43586438	73.7	0.654	Middle	African region	1
4	Andorra	84396	87.9	0.867	High	European Region	90
5	Angola	31677923	66.8	0.447	Low	African region	1
6	Argentina	45283980	92.1	0.721	High-middle	American region	37
7	Armenia	3011472	63.3	0.698	Middle	European Region	478
8	Australia	25369997	86.2	0.842	High	Western Pacific Region	100
9	Austria	8943874	58.7	0.852	High	European Region	70
10	Azerbaijan	10436676	56.4	0.693	Middle	European Region	1
11	Bahrain	1513957	89.5	0.748	High-middle	Eastern Mediterranean Region	63
12	Bangladesh	163458437	38.2	0.483	Low-middle	Southeast Asian Region	4
13	Barbados	298133	31.2	0.744	High-middle	American region	119
14	Belarus	9398586	79.5	0.782	High-middle	European Region	11
15	Belgium	11443597	98.1	0.851	High	European Region	6
16	Belize	420981	46	0.607	Low-middle	American region	69
17	Benin	13086665	48.4	0.367	Low	African region	1
18	Bhutan	758288	42.3	0.469	Low-middle	Southeast Asian Region	82
19	Bolivia (Plurinational State of)	11680236	70.1	0.595	Low-middle	American region	58

	Location	Population	Urbanization (%)	SDI	SDI Region	WHO Region	Sample
20	Bosnia and Herzegovina	3349757	49	0.72	High-middle	European Region	10
21	Botswana	2363151	70.9	0.639	Middle	African region	4957
22	Brazil	218698563	87.1	0.649	Middle	American region	18
23	Brunei Darussalam	446560	78.3	0.807	High-middle	Western Pacific Region	258
24	Bulgaria	6872910	75.7	0.765	High-middle	European Region	35
25	Burkina Faso	22156215	30.6	0.28	Low	African region	46
26	Burundi	12803358	13.7	0.286	Low	African region	102
27	Cabo Verde	555867	66.7	0.528	Low-middle	African region	55
28	Cambodia	16831707	24.2	0.469	Low-middle	Western Pacific Region	55
29	Cameroon	30885759	57.6	0.472	Low-middle	African region	286
30	Canada	37144438	81.6	0.872	High	American region	9
31	Central African Republic	5393934	42.2	0.305	Low	African region	61
32	Chad	17107965	23.5	0.236	Low	African region	50
33	Chile	18660878	87.7	0.769	High-middle	American region	343
34	China	1419484780	61.4	0.713	High-middle	Western Pacific Region	757
35	Colombia	48782761	81.4	0.651	Middle	American region	2
36	Congo	5301123	67.8	0.578	Low-middle	African region	50
37	Costa Rica	4730756	80.8	0.696	Middle	American region	33
38	Croatia	4233336	57.6	0.795	High-middle	European Region	164
39	Cuba	11330600	77.2	0.665	Middle	American region	6

	Location	Population	Urbanization (%)	SDI	SDI Region	WHO Region	Sample
40	Cyprus	1338917	66.8	0.833	High	European Region	77
41	Czechia	10647069	74.1	0.827	High	European Region	271
42	CÔte d'Ivoire	27238160	51.7	0.419	Low	African region	21
43	Democratic People's Republic of Korea	26341771	62.4	0.568	Low-middle	Southeast Asian Region	5
44	Democratic Republic of the Congo	87957494	45.6	0.377	Low	African region	50
45	Denmark	5826973	88.1	0.894	High	European Region	3
46	Dominica	67327	71.1	0.744	High-middle	American region	147
47	Dominican Republic	10898829	82.5	0.616	Middle	American region	7
48	Ecuador	17838713	64.2	0.657	Middle	American region	271
49	Egypt	104036565	42.8	0.597	Low-middle	Eastern Mediterranean Region	160
50	El Salvador	6427781	73.4	0.559	Low-middle	American region	6
51	Equatorial Guinea	1472660	73.1	0.652	Middle	African region	46
52	Eritrea	6455682	41.3	0.4	Low	African region	104
53	Estonia	1313779	69.2	0.842	High	European Region	63
54	Eswatini	1149697	24.2	0.582	Low-middle	African region	289
55	Ethiopia	106721836	21.7	0.353	Low	African region	6
56	Fiji	921025	57.2	0.671	Middle	Western Pacific Region	19
57	Finland	5533961	85.5	0.858	High	European Region	96
58	France	66281784	81	0.836	High	European Region	31
59	Gabon	1791334	90.1	0.628	Middle	African region	23

	Location	Population	Urbanization (%)	SDI	SDI Region	WHO Region	Sample
60	Gambia	2338466	62.6	0.405	Low	African region	67
61	Georgia	3644844	59.5	0.729	High-middle	European Region	33
62	Germany	85202428	77.5	0.901	High	European Region	202
63	Ghana	33445268	57.3	0.558	Low-middle	African region	52
64	Greece	10250328	79.7	0.79	High-middle	European Region	2
65	Grenada	103146	36.5	0.665	Middle	American region	286
66	Guatemala	15620894	51.8	0.535	Low-middle	American region	36
67	Guinea	13084720	36.9	0.33	Low	African region	18
68	Guinea-Bissau	2018629	44.2	0.348	Low	African region	11
69	Guyana	768551	26.8	0.642	Middle	American region	39
70	Haiti	12631493	57.1	0.446	Low	American region	183
71	Honduras	9932531	58.4	0.509	Low-middle	American region	20
72	Hungary	9649798	71.9	0.788	High-middle	European Region	19
73	Iceland	347446	93.9	0.874	High	European Region	545
74	India	1402361683	34.9	0.568	Low-middle	Southeast Asian Region	386
75	Indonesia	275904986	56.6	0.652	Middle	Southeast Asian Region	280
76	Iran (Islamic Republic of)	84961080	75.9	0.692	Middle	Eastern Mediterranean Region	98
77	Iraq	40592068	70.9	0.653	Middle	Eastern Mediterranean Region	26
78	Ireland	4923036	63.7	0.872	High	European Region	6
79	Israel	9439442	92.6	0.806	High-middle	European Region	103

	Location	Population	Urbanization (%)	SDI	SDI Region	WHO Region	Sample
80	Italy	60073251	71	0.804	High-middle	European Region	13
81	Jamaica	2800558	56.3	0.681	Middle	American region	2895
82	Japan	128077172	91.8	0.869	High	Western Pacific Region	49
83	Jordan	11925923	91.4	0.72	High-middle	Eastern Mediterranean Region	133
84	Kazakhstan	18716891	57.7	0.723	High-middle	European Region	63
85	Kenya	49362322	28	0.516	Low-middle	African region	1
86	Kuwait	4520831	100	0.843	High	Eastern Mediterranean Region	24
87	Kyrgyzstan	6761669	36.9	0.6	Low-middle	European Region	52
88	Lao People's Democratic Republic	7280312	36.3	0.484	Low-middle	Western Pacific Region	26
89	Latvia	1892857	68.3	0.828	High	European Region	26
90	Lebanon	5548160	88.9	0.742	High-middle	Eastern Mediterranean Region	31
91	Lesotho	1873654	29	0.507	Low-middle	African region	52
92	Liberia	5327425	52.1	0.348	Low	African region	24
93	Libya	6839464	80.7	0.72	High-middle	Eastern Mediterranean Region	55
94	Lithuania	2763281	68	0.853	High	European Region	12
95	Luxembourg	631369	91.5	0.882	High	European Region	83
96	Madagascar	27947615	38.5	0.393	Low	African region	27
97	Malawi	19017568	17.4	0.378	Low	African region	126
98	Malaysia	31540082	77.2	0.739	High-middle	Western Pacific Region	125
99	Mali	23251322	43.9	0.263	Low	African region	66

	Location	Population	Urbanization (%)	SDI	SDI Region	WHO Region	Sample
100	Malta	441199	94.7	0.798	High-middle	European Region	1
101	Mauritania	4304273	55.3	0.491	Low-middle	African region	9
102	Mauritius	1273381	40.8	0.715	High-middle	African region	9
103	Mexico	128564796	80.7	0.66	Middle	American region	1580
104	Mongolia	3280627	68.7	0.615	Low-middle	Western Pacific Region	20
105	Montenegro	620805	67.5	0.793	High-middle	European Region	53
106	Morocco	36962173	63.5	0.555	Low-middle	Eastern Mediterranean Region	99
107	Mozambique	30289774	37.1	0.32	Low	African region	56
108	Myanmar	55877586	31.1	0.529	Low-middle	Southeast Asian Region	75
109	Namibia	2405808	52	0.614	Low-middle	African region	85
110	Nepal	30837933	20.6	0.427	Low	Southeast Asian Region	155
111	Netherlands	17176117	92.2	0.887	High	European Region	1185
112	New Zealand	5092805	86.7	0.847	High	Western Pacific Region	6
113	Nicaragua	6598464	59	0.52	Low-middle	American region	90
114	Niger	24101278	16.6	0.165	Low	African region	500
115	Nigeria	225038009	52	0.496	Low-middle	African region	37
116	North Macedonia	2183272	58.5	0.748	High-middle	European Region	478
117	Norway	5383237	83	0.915	High	European Region	100
118	Oman	4514370	86.3	0.769	High-middle	Eastern Mediterranean Region	70
119	Pakistan	230554972	37.2	0.497	Low-middle	Eastern Mediterranean Region	11

	Location	Population	Urbanization (%)	SDI	SDI Region	WHO Region	Sample
120	Palestine	5054318	76.7	0.624	Middle	#N/A	63
121	Panama	4234132	68.4	0.704	Middle	American region	4
122	Papua New Guinea	10174742	13.3	0.415	Low	Western Pacific Region	119
123	Paraguay	7089232	62.2	0.631	Middle	American region	11
124	Peru	35685165	78.3	0.659	Middle	American region	6
125	Philippines	111908917	47.4	0.645	Middle	Western Pacific Region	69
126	Poland	38372694	60	0.809	High	European Region	4
127	Portugal	10643097	66.3	0.741	High-middle	European Region	82
128	Puerto Rico	3338048	93.6	0.823	High	#N/A	58
129	Qatar	2903038	99.2	0.841	High	Eastern Mediterranean Region	10
130	Republic of Korea	51722592	81.4	0.884	High	Western Pacific Region	4957
131	Republic of Moldova	3624436	42.8	0.728	High-middle	European Region	18
132	Romania	19117662	54.2	0.764	High-middle	European Region	258
133	Russian Federation	145574315	74.8	0.806	High-middle	European Region	35
134	Rwanda	13001852	17.4	0.43	Low	African region	46
135	Saint Kitts and Nevis	58341	30.8	0.752	High-middle	American region	9
136	Saint Lucia	177321	18.8	0.67	Middle	American region	102
137	Saint Vincent and the Grenadines	114154	53	0.633	Middle	American region	55
138	Saudi Arabia	36979369	84.3	0.811	High	Eastern Mediterranean Region	286
139	Senegal	15569358	48.1	0.401	Low	African region	55

	Location	Population	Urbanization (%)	SDI	SDI Region	WHO Region	Sample
140	Serbia	8975814	56.4	0.789	High-middle	European Region	61
141	Sierra Leone	8625353	42.9	0.352	Low	African region	50
142	Singapore	5697917	100	0.854	High	Western Pacific Region	343
143	Slovakia	5439138	53.8	0.808	High	European Region	757
144	Slovenia	2072558	55.1	0.84	High	European Region	41
145	Somalia	20803510	46.1	0.076	Low	Eastern Mediterranean Region	76
146	South Africa	56505459	67.4	0.677	Middle	African region	21
147	South Sudan	9412860	20.2	0.277	Low	African region	164
148	Spain	45780860	80.8	0.767	High-middle	European Region	6
149	Sri Lanka	22147111	18.7	0.698	Middle	Southeast Asian Region	77
150	Sudan	42694325	35.3	0.533	Low-middle	Eastern Mediterranean Region	33
151	Suriname	577123	66.1	0.631	Middle	American region	173
152	Sweden	10300025	88	0.885	High	European Region	50
153	Switzerland	8851291	73.9	0.932	High	European Region	271
154	Syrian Arab Republic	13988260	55.5	0.618	Middle	Eastern Mediterranean Region	6
155	Tajikistan	9946657	27.5	0.538	Low-middle	European Region	147
156	Thailand	66728031	51.4	0.679	Middle	Southeast Asian Region	188
157	Timor-Leste	1369331	31.3	0.442	Low	Southeast Asian Region	271
158	Togo	8198069	42.8	0.402	Low	African region	160
159	Trinidad and Tobago	1394454	53.2	0.766	High-middle	American region	6

	Location	Population	Urbanization (%)	SDI	SDI Region	WHO Region	Sample
160	Tunisia	11783954	69.6	0.678	Middle	Eastern Mediterranean Region	46
161	Turkey	83201046	76.1	0.706	High-middle	European Region	104
162	Turkmenistan	5114271	52.5	0.678	Middle	European Region	16
163	Uganda	42303604	25	0.417	Low	African region	63
164	Ukraine	43504149	69.6	0.76	High-middle	European Region	6
165	United Arab Emirates	9571232	87	0.846	High	Eastern Mediterranean Region	19
166	United Kingdom	67578977	83.9	0.857	High	European Region	96
167	United Republic of Tanzania	57176562	35.2	0.439	Low	African region	31
168	United States of America	331500751	82.7	0.861	High	American region	23
169	United States Virgin Islands	87998	95.9	0.82	High	#N/A	67
170	Uruguay	3410594	95.5	0.716	High-middle	American region	33
171	Uzbekistan	33667290	50.4	0.659	Middle	European Region	202
172	Venezuela (Bolivarian Republic of)	27016111	88.3	0.6	Low-middle	American region	52
173	Viet Nam	99393194	37.3	0.623	Middle	Western Pacific Region	6
174	Yemen	32968531	37.9	0.447	Low	Eastern Mediterranean Region	286
175	Zambia	19027694	44.6	0.497	Low-middle	African region	36
176	Zimbabwe	15379294	32.2	0.472	Low-middle	African region	18

Notes: SDI, socio-demographic index; WHO, world health organization.

Table S3. A summary of the studies that met the criteria of the systematic review on the associations between greenness exposure (measured by NDVI) and depression.

Author	Year Published	Country or region	Study period	Study design	Age	Sample size	Female, %	Exposure	Measure/Definition of exposure	Outcome
**Banay, R.F., et.al.**	2019	U.S.	2000- 2010	Cohort	54–91 (mean age throughout follow-up 70)	38947	100%	NDVI	250m	Depression
**Bezold C P, et.al**	2018	the United States	1999	cross sectional	12-18 (mean 14.9)	9385	59%	NDVI	250m	Major Depression
**Brown, S. C, et.al**	2018	America	2010-2011	Cohort	65+ (median age 76.33)	249405	0.5833	NDVI	All Neighborhood Income Levels	Depression
**Dzhambov, A, et.al**	2018	Bulgaria	2018	cross sectional	18-35 (21.74±3.00)	529	0.664	NDVI	500m	Depression
**Gascon, M, et.al**	2018	Spain	2013-2014	cross sectional	45-74	874	0.639	NDVI	500m	Depression
**Gonzales-Inca C, et.al**	2022	Finland	1998-2012	Cohort	20-54	14424	61.90%	NDVI	500m	Depression
**Helbich, M.; et.al**	2019	China	March to August 2011	cross sectional	>60 (mean 70.7)	1190	59.70%	NDVI	average NDVI indistrict	Depression
**Lõhmus M, Stenfors CU, et.al**	2021	Sweden	5 June to 1 August 2020	cross sectional	≥18	2060	55.30%	NDVI	50m	Depression
**Song, H., et.al**	2019	South Korea	2009	cross sectional	Mean 46.2	65128	53.50%	NDVI	average NDV Iindistrict	Depression

Notes: NDVI, Normalized Difference Vegetation Index.

Table S4. The results of Newcastle Ottawa Scale (NOS) for quality assessment of all include articles in meta-analysis.

cross sectional

	**Selection**				**Comparability**	**Exposure**		**Total score**
**ID**	Representativeness of the sample	Sample size	Non-respondents	Ascertainment of the adversity exposure (risk factor)	Control for important factor	Assessment of the outcome	Statistical test	
2	**1**	**1**	**0**	**2**	**2**	**1**	**0**	**7**
4	**1**	**0**	**1**	**2**	**2**	**1**	**1**	**8**
5	**1**	**0**	**1**	**2**	**2**	**0**	**1**	**7**
7	**1**	**1**	**1**	**2**	**2**	**1**	**1**	**9**
8	**1**	**1**	**0**	**2**	**2**	**1**	**1**	**8**
9	**1**	**1**	**0**	**2**	**2**	**1**	**0**	**7**

Cohort

	**Selection**				**Comparability**	**Exposure**			**Total score**
**ID**	Representativeness of the exposed cohort	Selection of the non exposed cohort	Ascertainment of exposure	Demonstration that outcome of interest was not present at start of study	Comparability of cohorts on the basis of the design or analysis	Assessment of outcome	Was follow-up long enough for outcomes to occur	Adequacy of follow up of cohorts	
1	1	1	1	1	2	0	1	0	7
3	1	1	1	0	0	1	0	0	4
6	1	1	1	1	2	1	1	0	8

Table S5. The PAF and ASDR for both sexes under three greenspace expansion scenarios in 176 countries in 2020.

location	Best Potential Scenario		Proportional Increase Scenario		Uniform Increase Scenario	
	PAF (95%CI)	ASDR (95%CI)	PAF (95%CI)	ASDR (95%CI)	PAF (95%CI)	ASDR (95%CI)
Afghanistan	‘-0.287 (-0.511,-0.097)	94.925 (32.795,175.063)	-0.014 (-0.023,-0.005)	4.640 (1.723,7.960)	-0.070 (-0.118,-0.025)	23.149 (8.451,40.375)
Albania	-0.258 (-0.459,-0.084)	91.532 (27.994,164.501)	-0.032 (-0.054,-0.011)	11.454 (3.718,19.363)	-0.069 (-0.116,-0.023)	24.347 (7.818,41.611)
Algeria	-0.435 (-0.834,-0.135)	341.368 (105.415,674.943)	-0.020 (-0.034,-0.007)	15.920 (5.467,27.776)	-0.067 (-0.115,-0.023)	52.461 (17.735,92.961)
Andorra	-0.067 (-0.115,-0.024)	60.069 (20.762,105.571)	-0.044 (-0.075,-0.015)	39.411 (13.661,68.985)	-0.069 (-0.118,-0.024)	61.593 (21.213,108.758)
Angola	-0.366 (-0.700,-0.116)	373.639 (110.354,738.803)	-0.016 (-0.027,-0.005)	15.945 (5.257,28.498)	-0.068 (-0.119,-0.023)	69.205 (22.385,125.695)
Argentina	-0.274 (-0.481,-0.084)	177.804 (53.478,321.356)	-0.029 (-0.047,-0.009)	18.547 (5.961,31.491)	-0.069 (-0.115,-0.022)	44.490 (14.115,76.534)
Armenia	-0.231 (-0.395,-0.068)	108.678 (32.737,195.276)	-0.023 (-0.038,-0.007)	11.021 (3.527,18.757)	-0.068 (-0.112,-0.021)	32.283 (10.184,55.744)
Australia	-0.323 (-0.573,-0.113)	307.171 (100.393,561.543)	-0.039 (-0.063,-0.015)	36.686 (12.895,62.436)	-0.069 (-0.114,-0.026)	65.390 (22.779,112.352)
Austria	-0.197 (-0.337,-0.059)	84.717 (25.939,150.591)	-0.039 (-0.064,-0.012)	16.627 (5.305,28.406)	-0.069 (-0.115,-0.022)	29.861 (9.442,51.552)
Azerbaijan	-0.233 (-0.413,-0.083)	88.045 (31.295,157.436)	-0.022 (-0.036,-0.008)	8.192 (3.097,13.892)	-0.068 (-0.116,-0.025)	25.607 (9.526,44.077)
Bahrain	-0.053 (-0.093,-0.021)	55.775 (22.819,97.398)	-0.010 (-0.017,-0.004)	10.303 (4.267,17.802)	-0.069 (-0.122,-0.027)	72.752 (29.511,127.989)
Bangladesh	-0.195 (-0.347,-0.064)	91.090 (30.180,166.414)	-0.032 (-0.054,-0.011)	14.889 (5.165,26.063)	-0.068 (-0.118,-0.023)	31.903 (10.935,56.500)

	Best Potential Scenario		Proportional Increase Scenario		Uniform Increase Scenario	
**location**	**PAF (95%CI)**	**ASDR (95%CI)**	**PAF (95%CI)**	**ASDR (95%CI)**	**PAF (95%CI)**	**ASDR (95%CI)**
Barbados	-0.067 (-0.109,-0.026)	15.015 (5.670,26.001)	-0.033 (-0.053,-0.013)	7.344 (2.788,12.675)	-0.069 (-0.113,-0.026)	15.477 (5.810,26.942)
Belarus	-0.105 (-0.175,-0.034)	83.995 (27.537,143.623)	-0.033 (-0.055,-0.011)	26.709 (8.920,44.823)	-0.070 (-0.116,-0.023)	55.830 (18.434,94.843)
Belgium	-0.078 (-0.129,-0.025)	77.605 (25.764,128.948)	-0.044 (-0.072,-0.014)	43.562 (14.520,72.161)	-0.069 (-0.114,-0.022)	68.580 (22.679,114.401)
Belize	-0.080 (-0.139,-0.030)	30.057 (11.175,51.379)	-0.041 (-0.072,-0.016)	15.614 (5.816,26.660)	-0.068 (-0.120,-0.025)	25.645 (9.467,44.129)
Benin	-0.235 (-0.414,-0.077)	117.816 (38.357,214.955)	-0.027 (-0.045,-0.009)	13.570 (4.673,23.215)	-0.069 (-0.117,-0.024)	34.762 (11.825,60.481)
Bhutan	-0.039 (-0.068,-0.014)	16.082 (5.579,27.769)	-0.031 (-0.054,-0.011)	12.970 (4.510,22.354)	-0.067 (-0.117,-0.024)	27.688 (9.513,48.160)
Bolivia (Plurinational State of)	-0.359 (-0.629,-0.105)	253.895 (75.330,467.851)	-0.023 (-0.037,-0.007)	16.086 (5.239,27.333)	-0.069 (-0.113,-0.022)	48.858 (15.645,84.119)
Bosnia and Herzegovina	-0.142 (-0.238,-0.044)	44.799 (14.187,79.519)	-0.043 (-0.070,-0.014)	13.460 (4.387,23.321)	-0.072 (-0.118,-0.023)	22.613 (7.302,39.507)
Botswana	-0.117 (-0.200,-0.036)	81.137 (25.795,139.576)	-0.025 (-0.042,-0.008)	17.468 (5.720,29.357)	-0.069 (-0.117,-0.022)	48.093 (15.506,81.601)
Brazil	-0.393 (-0.729,-0.124)	314.828 (99.575,595.153)	-0.034 (-0.057,-0.012)	27.215 (9.515,46.743)	-0.069 (-0.117,-0.024)	55.191 (19.081,95.784)
Brunei Darussalam	-0.180 (-0.321,-0.058)	50.813 (17.077,93.219)	-0.045 (-0.076,-0.015)	12.592 (4.403,22.140)	-0.069 (-0.118,-0.023)	19.323 (6.709,34.275)
Bulgaria	-0.204 (-0.354,-0.067)	99.323 (31.355,174.850)	-0.034 (-0.056,-0.012)	16.632 (5.498,27.914)	-0.069 (-0.115,-0.023)	33.593 (10.988,56.966)

location	Best Potential Scenario		Proportional Increase Scenario		Uniform Increase Scenario	
	PAF (95%CI)	ASDR (95%CI)	PAF (95%CI)	ASDR (95%CI)	PAF (95%CI)	ASDR (95%CI)
Burkina Faso	-0.167 (-0.286,-0.057)	51.748 (17.791,90.271)	-0.017 (-0.027,-0.006)	5.178 (1.862,8.665)	-0.070 (-0.116,-0.025)	21.627 (7.654,36.774)
Burundi	-0.241 (-0.442,-0.067)	37.440 (10.237,69.758)	-0.031 (-0.053,-0.009)	4.800 (1.387,8.381)	-0.067 (-0.116,-0.019)	10.378 (2.961,18.350)
Cabo Verde	-0.038 (-0.062,-0.013)	32.455 (11.603,53.375)	-0.028 (-0.047,-0.009)	23.745 (8.180,40.617)	-0.067 (-0.116,-0.022)	58.098 (19.755,100.826)
Cambodia	-0.272 (-0.488,-0.096)	40.272 (14.380,72.384)	-0.024 (-0.039,-0.009)	3.479 (1.328,5.869)	-0.069 (-0.118,-0.026)	10.252 (3.855,17.545)
Cameroon	-0.272 (-0.472,-0.101)	170.563 (63.647,299.762)	-0.023 (-0.038,-0.009)	14.528 (5.801,23.917)	-0.070 (-0.115,-0.027)	43.662 (17.182,72.798)
Canada	-0.297 (-0.534,-0.094)	209.800 (67.892,381.902)	-0.029 (-0.049,-0.010)	20.618 (7.186,34.810)	-0.069 (-0.117,-0.023)	48.803 (16.783,83.339)
Central African Republic	-0.274 (-0.492,-0.085)	183.528 (57.525,333.921)	-0.031 (-0.052,-0.010)	20.570 (6.943,35.094)	-0.067 (-0.113,-0.022)	44.513 (14.831,76.671)
Chad	-0.331 (-0.611,-0.090)	99.383 (27.216,184.002)	-0.021 (-0.035,-0.006)	6.180 (1.866,10.527)	-0.070 (-0.120,-0.021)	21.034 (6.244,36.418)
Chile	-0.267 (-0.471,-0.083)	256.067 (80.794,468.768)	-0.023 (-0.038,-0.008)	22.141 (7.457,37.974)	-0.069 (-0.116,-0.023)	66.433 (22.026,115.662)
China	-0.117 (-0.201,-0.038)	39.370 (12.779,68.153)	-0.027 (-0.045,-0.009)	9.067 (3.013,15.302)	-0.068 (-0.116,-0.022)	22.844 (7.496,39.132)
Colombia	-0.432 (-0.817,-0.124)	185.622 (50.570,359.817)	-0.030 (-0.051,-0.010)	12.848 (3.926,22.044)	-0.068 (-0.116,-0.022)	29.138 (8.792,50.684)
Congo	-0.399 (-0.731,-0.114)	399.565 (112.241,735.328)	-0.022 (-0.036,-0.007)	21.914 (6.892,36.379)	-0.070 (-0.116,-0.022)	69.497 (21.497,116.855)

	Best Potential Scenario		Proportional Increase Scenario		Uniform Increase Scenario	
location	PAF (95%CI)	ASDR (95%CI)	PAF (95%CI)	ASDR (95%CI)	PAF (95%CI)	ASDR (95%CI)
Costa Rica	-0.269<br>(-0.481,-0.071)	178.838<br>(48.679,326.347)	-0.036<br>(-0.060,-0.010)	23.953<br>(6.957,41.084)	-0.069<br>(-0.117,-0.019)	45.827<br>(13.138,79.369)
Croatia	-0.072<br>(-0.125,-0.026)	29.289<br>(10.624,51.506)	-0.041<br>(-0.070,-0.015)	16.509<br>(6.022,28.897)	-0.069<br>(-0.120,-0.025)	28.012<br>(10.136,49.365)
Cuba	-0.208<br>(-0.372,-0.063)	142.823<br>(42.857,261.718)	-0.041<br>(-0.070,-0.013)	28.073<br>(8.795,49.514)	-0.069<br>(-0.119,-0.021)	47.358<br>(14.706,84.337)
Cyprus	-0.073<br>(-0.124,-0.025)	36.910<br>(12.664,65.164)	-0.024<br>(-0.041,-0.008)	12.242<br>(4.263,21.244)	-0.068<br>(-0.115,-0.023)	34.117<br>(11.711,60.211)
Czechia	-0.062<br>(-0.105,-0.022)	31.362<br>(11.186,53.792)	-0.041<br>(-0.070,-0.015)	20.900<br>(7.465,35.797)	-0.067<br>(-0.115,-0.024)	34.254<br>(12.128,59.225)
CÔte d'Ivoire	-0.194<br>(-0.331,-0.061)	87.814<br>(27.721,155.360)	-0.025<br>(-0.041,-0.008)	11.365<br>(3.736,19.440)	-0.070<br>(-0.117,-0.023)	31.748<br>(10.263,54.989)
Democratic People's Republic of Korea	-0.176<br>(-0.299,-0.067)	56.761<br>(22.288,99.056)	-0.024<br>(-0.039,-0.009)	7.696<br>(3.144,12.875)	-0.069<br>(-0.114,-0.027)	22.302<br>(8.990,37.763)
Democratic Republic of the Congo	-0.217<br>(-0.374,-0.061)	141.513<br>(38.324,251.239)	-0.034<br>(-0.056,-0.010)	22.250<br>(6.329,37.757)	-0.070<br>(-0.116,-0.020)	45.463<br>(12.767,78.081)
Denmark	-0.135<br>(-0.237,-0.049)	102.786<br>(38.518,185.975)	-0.040<br>(-0.069,-0.015)	30.461<br>(11.675,53.964)	-0.069<br>(-0.119,-0.025)	52.307<br>(19.857,93.474)
Dominica	-0.152<br>(-0.273,-0.061)	77.299<br>(30.425,139.909)	-0.045<br>(-0.078,-0.018)	22.769<br>(9.139,40.085)	-0.068<br>(-0.119,-0.027)	34.382<br>(13.722,61.049)
Dominican Republic	-0.376<br>(-0.692,-0.132)	306.559<br>(103.817,588.459)	-0.035<br>(-0.058,-0.013)	28.115<br>(10.443,49.184)	-0.068<br>(-0.116,-0.026)	55.622<br>(20.433,98.392)
Ecuador	-0.438<br>(-0.813,-0.130)	261.416<br>(76.454,506.338)	-0.026<br>(-0.043,-0.009)	15.375<br>(5.075,26.563)	-0.070<br>(-0.118,-0.023)	41.908<br>(13.619,73.569)

location	Best Potential Scenario		Proportional Increase Scenario		Uniform Increase Scenario	
	PAF (95%CI)	ASDR (95%CI)	PAF (95%CI)	ASDR (95%CI)	PAF (95%CI)	ASDR (95%CI)
Egypt	-0.282 (-0.502,-0.097)	120.971 (42.593,220.843)	-0.019 (-0.032,-0.007)	8.244 (3.086,14.077)	-0.069 (-0.116,-0.025)	29.525 (10.908,51.319)
El Salvador	-0.276 (-0.481,-0.082)	186.018 (53.760,336.321)	-0.044 (-0.072,-0.014)	29.595 (9.120,50.681)	-0.069 (-0.115,-0.022)	46.764 (14.288,80.649)
Equatorial Guinea	-0.183 (-0.335,-0.063)	200.373 (68.833,381.387)	-0.028 (-0.048,-0.010)	30.302 (10.890,54.544)	-0.068 (-0.120,-0.024)	74.631 (26.490,136.405)
Eritrea	-0.158 (-0.270,-0.054)	71.974 (23.812,126.624)	-0.017 (-0.028,-0.006)	7.800 (2.697,13.250)	-0.068 (-0.113,-0.024)	30.905 (10.491,53.093)
Estonia	-0.161 (-0.275,-0.050)	98.428 (30.669,168.080)	-0.034 (-0.056,-0.011)	20.787 (6.711,34.241)	-0.068 (-0.114,-0.022)	41.788 (13.345,69.622)
Eswatini	-0.174 (-0.317,-0.054)	48.910 (15.212,88.205)	-0.040 (-0.070,-0.013)	11.347 (3.671,19.730)	-0.068 (-0.119,-0.022)	18.981 (6.087,33.258)
Ethiopia	-0.292 (-0.506,-0.097)	72.009 (23.742,127.949)	-0.030 (-0.048,-0.011)	7.355 (2.597,12.103)	-0.069 (-0.114,-0.024)	17.131 (5.975,28.562)
Fiji	-0.173 (-0.301,-0.054)	57.359 (17.646,100.459)	-0.045 (-0.075,-0.015)	14.806 (4.742,25.115)	-0.069 (-0.116,-0.022)	22.808 (7.248,38.932)
Finland	-0.029 (-0.048,-0.009)	27.286 (8.206,46.907)	-0.031 (-0.052,-0.009)	29.425 (8.822,50.696)	-0.068 (-0.115,-0.020)	64.123 (18.985,111.514)
France	-0.131 (-0.226,-0.045)	112.400 (38.253,195.620)	-0.040 (-0.068,-0.014)	34.561 (11.958,59.011)	-0.069 (-0.118,-0.024)	59.076 (20.255,102.064)
Gabon	-0.296 (-0.543,-0.102)	383.911 (130.372,726.074)	-0.027 (-0.045,-0.010)	34.501 (12.623,59.708)	-0.068 (-0.117,-0.025)	88.689 (32.026,155.328)
Gambia	-0.139 (-0.243,-0.042)	122.124 (37.193,218.622)	-0.025 (-0.042,-0.008)	21.632 (6.818,37.493)	-0.069 (-0.118,-0.021)	60.287 (18.716,105.923)

	Best Potential Scenario		Proportional Increase Scenario		Uniform Increase Scenario	
**location**	**PAF (95%CI)**	**ASDR (95%CI)**	**PAF (95%CI)**	**ASDR (95%CI)**	**PAF (95%CI)**	**ASDR (95%CI)**
Georgia	-0.273 (-0.485,-0.090)	133.585 (46.802,241.676)	-0.030 (-0.050,-0.011)	14.658 (5.478,24.960)	-0.068 (-0.115,-0.024)	33.213 (12.250,57.212)
Germany	-0.038 (-0.062,-0.015)	25.172 (10.039,42.142)	-0.044 (-0.072,-0.017)	29.002 (11.494,48.829)	-0.069 (-0.115,-0.027)	45.642 (17.959,77.358)
Ghana	-0.265 (-0.463,-0.107)	157.907 (63.172,281.122)	-0.025 (-0.042,-0.011)	15.200 (6.431,25.361)	-0.070 (-0.116,-0.029)	41.688 (17.419,70.407)
Greece	-0.216 (-0.368,-0.075)	231.048 (78.907,410.686)	-0.030 (-0.049,-0.011)	32.509 (11.662,55.300)	-0.070 (-0.114,-0.025)	74.419 (26.357,127.809)
Grenada	-0.143 (-0.255,-0.041)	39.287 (10.849,68.134)	-0.050 (-0.086,-0.015)	13.592 (3.861,23.001)	-0.067 (-0.117,-0.020)	18.439 (5.206,31.352)
Guatemala	-0.370 (-0.693,-0.135)	188.562 (66.594,355.574)	-0.036 (-0.062,-0.014)	18.484 (7.152,32.125)	-0.069 (-0.118,-0.027)	34.854 (13.347,61.339)
Guinea	-0.204 (-0.376,-0.066)	78.056 (24.760,148.544)	-0.024 (-0.042,-0.008)	9.311 (3.084,16.671)	-0.067 (-0.119,-0.023)	25.873 (8.460,47.201)
Guinea-Bissau	-0.274 (-0.501,-0.091)	130.789 (42.137,247.571)	-0.026 (-0.044,-0.009)	12.283 (4.254,21.648)	-0.069 (-0.117,-0.024)	32.572 (11.136,58.114)
Guyana	-0.217 (-0.365,-0.075)	72.073 (24.813,127.402)	-0.035 (-0.057,-0.013)	11.729 (4.234,19.769)	-0.070 (-0.114,-0.025)	23.421 (8.367,39.850)
Haiti	-0.361 (-0.662,-0.122)	176.035 (57.779,321.369)	-0.033 (-0.055,-0.012)	15.987 (5.732,26.536)	-0.069 (-0.117,-0.025)	33.626 (11.916,56.483)
Honduras	-0.316 (-0.572,-0.096)	152.409 (47.378,282.250)	-0.038 (-0.063,-0.012)	18.172 (6.089,30.983)	-0.068 (-0.115,-0.022)	32.758 (10.863,56.358)
Hungary	-0.061 (-0.102,-0.019)	28.521 (9.374,48.466)	-0.039 (-0.066,-0.012)	18.426 (6.050,31.344)	-0.069 (-0.117,-0.021)	32.380 (10.529,55.541)

location	Best Potential Scenario		Proportional Increase Scenario		Uniform Increase Scenario	
	PAF (95%CI)	ASDR (95%CI)	PAF (95%CI)	ASDR (95%CI)	PAF (95%CI)	ASDR (95%CI)
Iceland	-0.177<br>(-0.314,-0.056)	118.007<br>(37.117,209.876)	-0.025<br>(-0.042,-0.008)	16.629<br>(5.484,28.061)	-0.070<br>(-0.119,-0.023)	46.401<br>(15.078,79.622)
India	-0.299<br>(-0.534,-0.099)	96.658<br>(31.205,178.055)	-0.031<br>(-0.051,-0.011)	10.040<br>(3.494,17.047)	-0.068<br>(-0.115,-0.024)	22.138<br>(7.605,38.031)
Indonesia	-0.402<br>(-0.747,-0.117)	111.578<br>(33.773,209.324)	-0.034<br>(-0.058,-0.011)	9.504<br>(3.184,16.151)	-0.069<br>(-0.117,-0.022)	19.109<br>(6.331,32.879)
Iran (Islamic Republic of)	-0.305<br>(-0.559,-0.098)	303.498<br>(99.814,548.036)	-0.015<br>(-0.026,-0.005)	15.179<br>(5.391,25.282)	-0.069<br>(-0.117,-0.023)	68.014<br>(23.774,115.270)
Iraq	-0.168<br>(-0.298,-0.052)	124.314<br>(38.862,224.688)	-0.012<br>(-0.021,-0.004)	9.027<br>(2.950,15.452)	-0.069<br>(-0.119,-0.022)	51.154<br>(16.439,89.595)
Ireland	-0.116<br>(-0.199,-0.037)	86.704<br>(29.127,152.655)	-0.048<br>(-0.081,-0.016)	35.668<br>(12.154,62.001)	-0.069<br>(-0.117,-0.022)	51.158<br>(17.322,89.420)
Israel	-0.034<br>(-0.056,-0.012)	35.036<br>(12.405,58.449)	-0.026<br>(-0.042,-0.009)	26.121<br>(9.227,43.688)	-0.069<br>(-0.115,-0.023)	70.977<br>(24.707,120.568)
Italy	-0.108<br>(-0.183,-0.034)	79.247<br>(25.387,135.654)	-0.037<br>(-0.062,-0.012)	27.224<br>(8.868,45.835)	-0.069<br>(-0.115,-0.022)	50.477<br>(16.282,85.794)
Jamaica	-0.168<br>(-0.289,-0.053)	65.111<br>(20.577,114.464)	-0.042<br>(-0.070,-0.014)	16.253<br>(5.329,27.814)	-0.070<br>(-0.117,-0.023)	26.925<br>(8.746,46.469)
Japan	-0.478<br>(-0.909,-0.161)	238.701<br>(79.303,453.591)	-0.028<br>(-0.046,-0.010)	13.800<br>(5.134,23.029)	-0.068<br>(-0.116,-0.025)	34.118<br>(12.538,57.844)
Jordan	-0.176<br>(-0.307,-0.060)	154.728<br>(52.238,282.344)	-0.015<br>(-0.025,-0.005)	13.196<br>(4.678,22.910)	-0.069<br>(-0.117,-0.024)	60.631<br>(21.099,107.063)
Kazakhstan	-0.090<br>(-0.154,-0.034)	43.320<br>(16.251,76.260)	-0.020<br>(-0.033,-0.008)	9.483<br>(3.618,16.414)	-0.068<br>(-0.118,-0.026)	33.014<br>(12.399,58.061)

	Best Potential Scenario		Proportional Increase Scenario		Uniform Increase Scenario	
**location**	**PAF (95%CI)**	**ASDR (95%CI)**	**PAF (95%CI)**	**ASDR (95%CI)**	**PAF (95%CI)**	**ASDR (95%CI)**
Kenya	-0.344 (-0.623,-0.103)	112.892 (32.907,206.074)	-0.033 (-0.055,-0.011)	10.956 (3.482,18.579)	-0.068 (-0.114,-0.022)	22.245 (6.988,38.072)
Kuwait	-0.008 (-0.013,-0.002)	7.987 (2.132,13.608)	-0.008 (-0.014,-0.002)	8.571 (2.281,14.643)	-0.068 (-0.119,-0.020)	69.380 (18.045,120.743)
Kyrgyzstan	-0.139 (-0.239,-0.039)	45.670 (13.506,82.250)	-0.021 (-0.035,-0.006)	7.008 (2.151,12.233)	-0.069 (-0.117,-0.020)	22.690 (6.858,40.234)
Lao People's Democratic Republic	-0.314 (-0.568,-0.115)	62.343 (22.928,114.560)	-0.031 (-0.052,-0.012)	6.231 (2.457,10.624)	-0.069 (-0.116,-0.027)	13.713 (5.355,23.564)
Latvia	-0.097 (-0.161,-0.029)	59.473 (16.904,102.915)	-0.038 (-0.062,-0.011)	23.156 (6.691,39.372)	-0.070 (-0.115,-0.021)	42.776 (12.239,73.500)
Lebanon	-0.121 (-0.209,-0.039)	129.087 (40.716,228.869)	-0.025 (-0.042,-0.008)	26.643 (8.601,46.314)	-0.069 (-0.118,-0.022)	73.270 (23.311,128.905)
Lesotho	-0.078 (-0.132,-0.030)	32.159 (12.165,56.370)	-0.030 (-0.050,-0.012)	12.423 (4.759,21.459)	-0.067 (-0.114,-0.026)	27.857 (10.563,48.635)
Liberia	-0.286 (-0.506,-0.088)	157.666 (47.144,279.973)	-0.030 (-0.049,-0.010)	16.520 (5.335,27.477)	-0.070 (-0.116,-0.023)	38.520 (12.293,64.885)
Libya	-0.043 (-0.072,-0.015)	39.218 (13.670,67.501)	-0.017 (-0.028,-0.006)	15.365 (5.362,26.415)	-0.069 (-0.119,-0.024)	63.559 (21.804,111.339)
Lithuania	-0.122 (-0.217,-0.039)	87.747 (28.585,160.006)	-0.036 (-0.062,-0.012)	25.695 (8.574,45.780)	-0.068 (-0.118,-0.022)	48.476 (15.998,87.175)
Luxembourg	-0.092 (-0.152,-0.032)	70.855 (22.483,119.204)	-0.045 (-0.073,-0.016)	34.356 (11.036,57.176)	-0.069 (-0.114,-0.024)	53.137 (16.938,89.030)
Madagascar	-0.319 (-0.575,-0.088)	142.057 (38.780,274.512)	-0.029 (-0.048,-0.009)	12.995 (3.849,22.935)	-0.068 (-0.115,-0.020)	30.458 (8.912,54.650)

	Best Potential Scenario		Proportional Increase Scenario		Uniform Increase Scenario	
location	PAF (95%CI)	ASDR (95%CI)	PAF (95%CI)	ASDR (95%CI)	PAF (95%CI)	ASDR (95%CI)
Malawi	-0.196 (-0.354,-0.061)	32.521 (9.860,59.793)	-0.031 (-0.053,-0.010)	5.128 (1.632,8.941)	-0.068 (-0.119,-0.022)	11.300 (3.552,19.972)
Malaysia	-0.315 (-0.564,-0.097)	172.858 (52.448,319.927)	-0.037 (-0.061,-0.012)	20.076 (6.604,34.604)	-0.070 (-0.118,-0.023)	38.433 (12.504,67.013)
Mali	-0.205 (-0.353,-0.074)	73.536 (26.242,127.345)	-0.021 (-0.033,-0.008)	7.345 (2.761,12.109)	-0.069 (-0.114,-0.026)	24.583 (9.107,40.978)
Malta	-0.143 (-0.234,-0.050)	104.863 (34.629,179.964)	-0.023 (-0.037,-0.008)	16.848 (5.729,28.267)	-0.070 (-0.112,-0.025)	51.158 (17.137,86.714)
Mauritania	-0.157 (-0.269,-0.057)	73.442 (27.090,132.943)	-0.008 (-0.014,-0.003)	3.894 (1.497,6.720)	-0.069 (-0.115,-0.026)	32.365 (12.224,57.040)
Mauritius	-0.079 (-0.133,-0.029)	29.469 (10.396,50.180)	-0.042 (-0.071,-0.016)	15.728 (5.574,26.694)	-0.068 (-0.114,-0.025)	25.211 (8.864,43.070)
Mexico	-0.551 (-1.059,-0.158)	451.571 (132.155,880.618)	-0.025 (-0.042,-0.008)	20.768 (6.967,35.131)	-0.069 (-0.116,-0.022)	56.383 (18.643,96.775)
Mongolia	-0.088 (-0.158,-0.028)	55.754 (17.583,101.666)	-0.013 (-0.023,-0.004)	8.413 (2.720,14.979)	-0.068 (-0.120,-0.022)	42.574 (13.501,77.115)
Montenegro	-0.119 (-0.202,-0.040)	55.511 (18.277,94.002)	-0.036 (-0.060,-0.012)	16.920 (5.672,28.189)	-0.067 (-0.113,-0.023)	31.295 (10.396,52.624)
Morocco	-0.113 (-0.200,-0.037)	103.489 (34.266,185.810)	-0.019 (-0.033,-0.006)	17.599 (5.978,30.746)	-0.069 (-0.120,-0.023)	62.978 (21.054,111.894)
Mozambique	-0.212 (-0.373,-0.073)	91.033 (30.695,167.508)	-0.030 (-0.051,-0.011)	13.053 (4.639,22.624)	-0.069 (-0.117,-0.025)	29.783 (10.457,52.221)
Myanmar	-0.125 (-0.210,-0.041)	16.759 (5.429,28.109)	-0.035 (-0.058,-0.012)	4.751 (1.580,7.792)	-0.069 (-0.114,-0.023)	9.253 (3.045,15.294)

location	Best Potential Scenario		Proportional Increase Scenario		Uniform Increase Scenario	
	PAF (95%CI)	ASDR (95%CI)	PAF (95%CI)	ASDR (95%CI)	PAF (95%CI)	ASDR (95%CI)
Namibia	-0.217 (-0.367,-0.075)	98.375 (34.766,168.242)	-0.018 (-0.029,-0.007)	8.180 (3.044,13.274)	-0.072 (-0.117,-0.026)	32.545 (11.913,53.557)
Nepal	-0.125 (-0.204,-0.047)	34.426 (12.889,58.217)	-0.028 (-0.044,-0.011)	7.666 (2.943,12.567)	-0.070 (-0.112,-0.027)	19.238 (7.303,31.994)
Netherlands	-0.055 (-0.089,-0.021)	48.641 (20.006,82.338)	-0.043 (-0.070,-0.017)	38.192 (15.692,64.715)	-0.070 (-0.114,-0.027)	61.843 (25.221,105.576)
New Zealand	-0.109 (-0.181,-0.036)	78.323 (25.968,135.761)	-0.047 (-0.077,-0.016)	33.641 (11.312,57.368)	-0.068 (-0.113,-0.023)	49.295 (16.468,84.664)
Nicaragua	-0.262 (-0.468,-0.094)	132.584 (44.693,252.704)	-0.036 (-0.061,-0.014)	18.456 (6.630,32.552)	-0.068 (-0.115,-0.026)	34.232 (12.174,61.077)
Niger	-0.067 (-0.110,-0.025)	10.884 (4.146,18.045)	-0.017 (-0.027,-0.007)	2.754 (1.063,4.512)	-0.070 (-0.116,-0.027)	11.421 (4.335,18.990)
Nigeria	-0.271 (-0.477,-0.079)	120.626 (34.863,214.483)	-0.023 (-0.037,-0.007)	10.023 (3.121,16.660)	-0.069 (-0.115,-0.021)	30.535 (9.361,51.621)
North Macedonia	-0.244 (-0.437,-0.080)	91.048 (28.392,165.680)	-0.033 (-0.055,-0.011)	12.127 (4.013,20.792)	-0.069 (-0.117,-0.024)	25.724 (8.408,44.636)
Norway	-0.221 (-0.384,-0.074)	173.828 (57.549,312.404)	-0.038 (-0.064,-0.013)	30.448 (10.573,51.744)	-0.069 (-0.116,-0.024)	54.700 (18.841,94.051)
Oman	-0.099 (-0.165,-0.033)	87.030 (29.837,151.460)	-0.013 (-0.022,-0.005)	11.882 (4.170,20.221)	-0.070 (-0.117,-0.024)	61.821 (21.280,107.172)
Pakistan	-0.245 (-0.434,-0.081)	93.423 (31.295,168.336)	-0.021 (-0.035,-0.007)	8.031 (2.863,13.618)	-0.069 (-0.116,-0.024)	26.154 (9.177,45.101)
Palestine	-0.055 (-0.093,-0.021)	71.423 (27.086,121.468)	-0.022 (-0.037,-0.009)	28.645 (10.942,48.430)	-0.069 (-0.116,-0.026)	88.712 (33.372,151.868)

	Best Potential Scenario		Proportional Increase Scenario		Uniform Increase Scenario	
**location**	**PAF (95%CI)**	**ASDR (95%CI)**	**PAF (95%CI)**	**ASDR (95%CI)**	**PAF (95%CI)**	**ASDR (95%CI)**
Panama	-0.278 (-0.509,-0.095)	155.047 (51.828,280.831)	-0.033 (-0.056,-0.012)	18.435 (6.578,31.352)	-0.069 (-0.119,-0.025)	38.572 (13.602,66.207)
Papua New Guinea	-0.265 (-0.479,-0.085)	21.118 (7.050,39.112)	-0.040 (-0.068,-0.014)	3.200 (1.126,5.508)	-0.068 (-0.117,-0.023)	5.436 (1.896,9.462)
Paraguay	-0.217 (-0.362,-0.075)	103.586 (35.767,178.730)	-0.041 (-0.065,-0.015)	19.495 (7.048,32.611)	-0.071 (-0.114,-0.025)	33.775 (12.094,56.849)
Peru	-0.563 (-1.103,-0.180)	256.686 (80.261,496.310)	-0.016 (-0.027,-0.006)	7.330 (2.669,12.198)	-0.067 (-0.115,-0.024)	30.565 (10.927,51.679)
Philippines	-0.342 (-0.607,-0.095)	107.878 (29.807,196.415)	-0.033 (-0.055,-0.010)	10.526 (3.171,17.849)	-0.070 (-0.115,-0.021)	21.918 (6.523,37.574)
Poland	-0.113 (-0.191,-0.035)	34.947 (10.603,57.771)	-0.039 (-0.065,-0.012)	12.006 (3.718,19.469)	-0.068 (-0.114,-0.021)	21.027 (6.448,34.419)
Portugal	-0.257 (-0.444,-0.077)	217.436 (64.307,390.198)	-0.037 (-0.060,-0.012)	30.977 (9.698,52.347)	-0.070 (-0.115,-0.022)	59.091 (18.289,100.688)
Puerto Rico	-0.173 (-0.297,-0.056)	107.771 (34.746,186.294)	-0.049 (-0.082,-0.016)	30.438 (10.106,51.567)	-0.069 (-0.116,-0.023)	42.939 (14.149,73.082)
Qatar	-0.012 (-0.021,-0.004)	12.269 (4.144,21.398)	-0.007 (-0.013,-0.003)	7.463 (2.518,13.029)	-0.068 (-0.120,-0.023)	67.639 (22.368,120.469)
Republic of Korea	-0.058 (-0.097,-0.018)	23.812 (7.170,40.083)	-0.028 (-0.047,-0.009)	11.598 (3.508,19.468)	-0.070 (-0.118,-0.022)	28.659 (8.544,48.704)
Republic of Moldova	-0.052 (-0.083,-0.019)	17.704 (6.657,30.002)	-0.033 (-0.053,-0.012)	11.381 (4.293,19.236)	-0.069 (-0.111,-0.025)	23.485 (8.758,40.090)
Romania	-0.090 (-0.147,-0.031)	31.319 (10.737,52.681)	-0.034 (-0.054,-0.012)	11.724 (4.061,19.547)	-0.070 (-0.115,-0.024)	24.414 (8.366,41.093)

location	Best Potential Scenario		Proportional Increase Scenario		Uniform Increase Scenario	
	PAF (95%CI)	ASDR (95%CI)	PAF (95%CI)	ASDR (95%CI)	PAF (95%CI)	ASDR (95%CI)
Russian Federation	-0.106 (-0.172,-0.033)	60.042 (18.237,100.306)	-0.029 (-0.046,-0.009)	16.171 (5.008,26.582)	-0.071 (-0.115,-0.022)	40.446 (12.351,67.309)
Rwanda	-0.183 (-0.310,-0.066)	37.813 (12.850,65.438)	-0.037 (-0.060,-0.014)	7.613 (2.686,12.721)	-0.071 (-0.117,-0.026)	14.696 (5.130,24.777)
Saint Kitts and Nevis	-0.128 (-0.222,-0.047)	37.387 (13.564,65.366)	-0.041 (-0.070,-0.016)	12.039 (4.466,20.509)	-0.068 (-0.116,-0.025)	19.857 (7.302,34.139)
Saint Lucia	-0.064 (-0.106,-0.022)	9.140 (3.230,15.613)	-0.043 (-0.071,-0.015)	6.128 (2.178,10.403)	-0.071 (-0.118,-0.024)	10.154 (3.578,17.388)
Saint Vincent and the Grenadines	-0.085 (-0.137,-0.028)	34.900 (11.366,58.344)	-0.048 (-0.078,-0.016)	19.947 (6.548,33.063)	-0.070 (-0.114,-0.023)	28.891 (9.421,48.244)
Saudi Arabia	-0.109 (-0.186,-0.038)	101.497 (35.685,171.897)	-0.008 (-0.013,-0.003)	7.510 (2.721,12.316)	-0.070 (-0.118,-0.025)	65.308 (23.223,109.296)
Senegal	-0.235 (-0.414,-0.083)	109.744 (39.495,192.808)	-0.017 (-0.028,-0.006)	7.801 (2.989,12.946)	-0.070 (-0.117,-0.026)	32.413 (12.226,54.631)
Serbia	-0.056 (-0.098,-0.017)	20.691 (6.309,37.435)	-0.036 (-0.064,-0.011)	13.415 (4.107,24.162)	-0.068 (-0.121,-0.020)	25.158 (7.626,45.826)
Sierra Leone	-0.187 (-0.315,-0.057)	83.528 (25.381,145.235)	-0.026 (-0.043,-0.008)	11.765 (3.717,19.886)	-0.069 (-0.114,-0.022)	30.792 (9.586,52.632)
Singapore	-0.022 (-0.037,-0.008)	10.814 (4.073,18.541)	-0.031 (-0.052,-0.011)	15.049 (5.624,26.007)	-0.069 (-0.117,-0.026)	33.751 (12.481,59.124)
Slovakia	-0.104 (-0.180,-0.036)	34.642 (11.761,61.154)	-0.040 (-0.068,-0.014)	13.375 (4.619,23.164)	-0.069 (-0.119,-0.024)	23.050 (7.882,40.367)
Slovenia	-0.179 (-0.311,-0.061)	75.404 (25.140,134.173)	-0.044 (-0.073,-0.016)	18.426 (6.371,31.559)	-0.070 (-0.118,-0.025)	29.340 (10.066,50.688)

	Best Potential Scenario		Proportional Increase Scenario		Uniform Increase Scenario	
location	PAF (95%CI)	ASDR (95%CI)	PAF (95%CI)	ASDR (95%CI)	PAF (95%CI)	ASDR (95%CI)
Somalia	-0.407<br>(-0.760,-0.116)	221.718<br>(61.049,423.718)	-0.018<br>(-0.030,-0.006)	9.665<br>(2.976,16.451)	-0.069<br>(-0.117,-0.022)	37.652<br>(11.385,65.030)
South Africa	-0.206<br>(-0.354,-0.073)	153.410<br>(53.564,273.361)	-0.032<br>(-0.052,-0.012)	23.566<br>(8.598,40.036)	-0.069<br>(-0.115,-0.025)	51.861<br>(18.692,89.104)
South Sudan	-0.181<br>(-0.318,-0.063)	41.881<br>(13.832,73.458)	-0.038<br>(-0.064,-0.014)	8.811<br>(3.024,14.852)	-0.069<br>(-0.116,-0.025)	15.860<br>(5.392,26.995)
Spain	-0.199<br>(-0.349,-0.072)	211.177<br>(74.890,373.828)	-0.030<br>(-0.051,-0.011)	32.034<br>(11.846,54.590)	-0.069<br>(-0.117,-0.026)	72.840<br>(26.611,125.930)
Sri Lanka	-0.117<br>(-0.200,-0.040)	13.210<br>(4.418,23.572)	-0.043<br>(-0.072,-0.015)	4.870<br>(1.658,8.553)	-0.068<br>(-0.116,-0.024)	7.726<br>(2.610,13.664)
Sudan	-0.196<br>(-0.338,-0.063)	78.925<br>(24.733,137.251)	-0.013<br>(-0.021,-0.004)	5.249<br>(1.743,8.740)	-0.068<br>(-0.114,-0.023)	27.457<br>(8.946,46.338)
Suriname	-0.288<br>(-0.520,-0.101)	225.577<br>(75.247,416.859)	-0.037<br>(-0.061,-0.014)	28.589<br>(10.192,49.502)	-0.069<br>(-0.117,-0.026)	53.979<br>(19.058,94.499)
Sweden	-0.151<br>(-0.256,-0.046)	153.963<br>(44.580,272.217)	-0.036<br>(-0.059,-0.011)	36.346<br>(10.888,62.294)	-0.069<br>(-0.114,-0.022)	70.180<br>(20.779,121.522)
Switzerland	-0.107<br>(-0.192,-0.038)	73.780<br>(26.017,134.510)	-0.045<br>(-0.080,-0.016)	31.009<br>(11.105,55.634)	-0.068<br>(-0.121,-0.024)	46.764<br>(16.622,84.443)
Syrian Arab Republic	-0.297<br>(-0.527,-0.084)	171.134<br>(48.370,318.699)	-0.019<br>(-0.031,-0.006)	10.905<br>(3.337,18.554)	-0.069<br>(-0.115,-0.021)	40.068<br>(12.068,69.078)
Tajikistan	-0.086<br>(-0.142,-0.026)	15.566<br>(4.782,26.078)	-0.022<br>(-0.037,-0.007)	4.072<br>(1.276,6.717)	-0.068<br>(-0.113,-0.021)	12.435<br>(3.833,20.781)
Thailand	-0.356<br>(-0.682,-0.113)	107.168<br>(32.878,207.641)	-0.031<br>(-0.053,-0.011)	9.181<br>(3.086,16.121)	-0.067<br>(-0.118,-0.023)	20.242<br>(6.720,36.075)

location	Best Potential Scenario		Proportional Increase Scenario		Uniform Increase Scenario	
	PAF (95%CI)	ASDR (95%CI)	PAF (95%CI)	ASDR (95%CI)	PAF (95%CI)	ASDR (95%CI)
Timor-Leste	-0.155<br>(-0.263,-0.049)	27.295<br>(9.037,46.974)	-0.030<br>(-0.050,-0.010)	5.330<br>(1.832,8.846)	-0.069<br>(-0.115,-0.022)	12.188<br>(4.131,20.462)
Togo	-0.277<br>(-0.494,-0.101)	125.841<br>(46.432,225.123)	-0.025<br>(-0.041,-0.010)	11.393<br>(4.498,19.050)	-0.069<br>(-0.115,-0.027)	31.270<br>(12.184,52.939)
Trinidad and Tobago	-0.163<br>(-0.281,-0.058)	77.534<br>(26.418,140.001)	-0.039<br>(-0.065,-0.014)	18.641<br>(6.537,32.698)	-0.069<br>(-0.117,-0.025)	32.971<br>(11.460,58.327)
Tunisia	-0.312<br>(-0.577,-0.093)	303.220<br>(89.673,570.693)	-0.019<br>(-0.032,-0.006)	18.635<br>(6.011,31.899)	-0.068<br>(-0.118,-0.022)	66.624<br>(21.135,116.646)
Turkey	-0.443<br>(-0.824,-0.133)	339.709<br>(107.112,640.331)	-0.024<br>(-0.039,-0.008)	18.029<br>(6.393,30.678)	-0.069<br>(-0.116,-0.023)	52.901<br>(18.469,91.160)
Turkmenistan	-0.016<br>(-0.026,-0.006)	6.194<br>(2.098,10.309)	-0.018<br>(-0.029,-0.006)	7.044<br>(2.379,11.758)	-0.070<br>(-0.117,-0.024)	27.555<br>(9.159,46.548)
Uganda	-0.216<br>(-0.375,-0.052)	91.756<br>(21.342,158.096)	-0.040<br>(-0.066,-0.010)	16.888<br>(4.159,27.590)	-0.069<br>(-0.116,-0.018)	29.417<br>(7.164,48.614)
Ukraine	-0.117<br>(-0.205,-0.041)	78.652<br>(28.184,139.018)	-0.036<br>(-0.061,-0.013)	24.035<br>(8.780,41.627)	-0.069<br>(-0.119,-0.024)	46.201<br>(16.708,80.881)
United Arab Emirates	-0.027<br>(-0.045,-0.009)	22.331<br>(7.420,37.263)	-0.010<br>(-0.017,-0.004)	8.498<br>(2.837,14.125)	-0.069<br>(-0.116,-0.024)	56.428<br>(18.494,95.226)
United Kingdom	-0.119<br>(-0.203,-0.033)	118.170<br>(32.026,205.057)	-0.045<br>(-0.075,-0.013)	44.495<br>(12.340,75.926)	-0.070<br>(-0.118,-0.019)	69.169<br>(19.006,118.922)
United Republic of Tanzania	-0.224<br>(-0.393,-0.068)	86.737<br>(28.046,152.342)	-0.034<br>(-0.056,-0.011)	13.116<br>(4.493,22.109)	-0.070<br>(-0.118,-0.022)	27.088<br>(9.168,46.068)
United States of America	-0.053<br>(-0.085,-0.015)	38.984<br>(11.341,65.357)	-0.052<br>(-0.084,-0.015)	38.361<br>(11.138,64.411)	-0.069<br>(-0.112,-0.020)	50.845<br>(14.673,85.743)

	Best Potential Scenario		Proportional Increase Scenario		Uniform Increase Scenario	
location	PAF (95%CI)	ASDR (95%CI)	PAF (95%CI)	ASDR (95%CI)	PAF (95%CI)	ASDR (95%CI)
United States Virgin Islands	-0.295 (-0.530,-0.092)	306.418 (98.230,561.693)	-0.035 (-0.059,-0.012)	36.597 (12.571,63.805)	-0.068 (-0.115,-0.022)	70.204 (23.891,123.770)
Uruguay	-0.079 (-0.132,-0.023)	56.400 (17.092,96.033)	-0.036 (-0.060,-0.011)	25.878 (7.893,43.808)	-0.070 (-0.117,-0.020)	49.766 (15.016,85.074)
Uzbekistan	-0.077 (-0.131,-0.029)	30.368 (11.376,51.278)	-0.022 (-0.036,-0.008)	8.518 (3.229,14.230)	-0.069 (-0.118,-0.025)	27.165 (10.145,46.008)
Venezuela (Bolivarian Republic of)	-0.386 (-0.718,-0.117)	266.412 (80.761,498.257)	-0.030 (-0.050,-0.010)	20.653 (6.870,35.362)	-0.069 (-0.117,-0.023)	47.663 (15.658,82.486)
Viet Nam	-0.306 (-0.555,-0.091)	52.388 (15.383,95.598)	-0.026 (-0.043,-0.008)	4.394 (1.399,7.489)	-0.070 (-0.119,-0.022)	11.908 (3.734,20.631)
Yemen	-0.461 (-0.868,-0.132)	204.418 (57.223,402.367)	-0.013 (-0.022,-0.004)	5.931 (1.875,10.252)	-0.070 (-0.118,-0.022)	31.113 (9.642,54.764)
Zambia	-0.239 (-0.429,-0.079)	102.246 (34.057,189.091)	-0.027 (-0.046,-0.010)	11.617 (4.111,20.040)	-0.068 (-0.116,-0.024)	29.069 (10.158,50.895)
Zimbabwe	-0.137 (-0.233,-0.049)	33.127 (11.576,58.255)	-0.031 (-0.051,-0.011)	7.483 (2.688,12.764)	-0.068 (-0.113,-0.025)	16.443 (5.843,28.371)

Notes: PAF, population attributable fraction; ASDR, age-standardised DALY rate. DALY, disability-adjusted life year.

Table S6. ASDR for males and females under three greenspace expansion scenarios in 176 countries in 2020.

	Best Potential Scenario		Proportional Increase Scenario		Uniform Increase Scenario	
**location**	**Female**	**Male**	**Female**	**Male**	**Female**	**Male**
Afghanistan	109.630<br>(37.884,198.077)	81.634<br>(27.917,150.333)	5.380<br>(1.999,8.975)	3.996<br>(1.465,6.772)	26.799<br>(9.803,45.650)	19.925<br>(7.194,34.453)
Albania	129.748<br>(39.911,238.735)	54.907<br>(17.766,100.827)	16.162<br>(5.321,28.262)	6.864<br>(2.360,11.815)	34.367<br>(11.181,60.693)	14.585<br>(4.963,25.448)
Algeria	418.307<br>(130.124,822.636)	271.275<br>(79.223,530.870)	19.583<br>(6.771,33.626)	12.659<br>(4.148,21.977)	64.479<br>(21.952,112.672)	41.664<br>(13.428,73.524)
Andorra	76.631<br>(27.307,131.220)	44.387<br>(16.490,77.247)	50.281<br>(17.972,85.828)	29.097<br>(10.847,50.485)	78.548<br>(27.892,135.179)	45.540<br>(16.853,79.561)
Angola	420.792<br>(130.097,816.352)	319.184<br>(96.318,629.860)	18.041<br>(6.155,31.977)	13.730<br>(4.537,24.000)	78.095<br>(26.223,140.353)	59.526<br>(19.346,105.945)
Argentina	216.959<br>(66.563,394.340)	135.140<br>(42.507,236.764)	22.640<br>(7.425,39.072)	14.111<br>(4.744,23.433)	54.330<br>(17.588,94.725)	33.852<br>(11.230,56.806)
Armenia	130.149<br>(40.446,236.805)	84.444<br>(26.572,149.947)	13.154<br>(4.341,22.556)	8.569<br>(2.856,14.327)	38.545<br>(12.544,67.245)	25.109<br>(8.250,42.524)
Australia	363.081<br>(126.013,661.600)	250.332<br>(86.761,442.227)	43.492<br>(16.204,73.645)	29.738<br>(11.142,49.696)	77.585<br>(28.619,132.767)	52.995<br>(19.684,89.315)
Austria	112.250<br>(34.121,196.837)	57.580<br>(18.402,101.484)	21.991<br>(6.997,37.127)	11.296<br>(3.754,19.152)	39.556<br>(12.446,67.426)	20.287<br>(6.686,34.753)
Azerbaijan	112.957<br>(38.143,196.377)	63.094<br>(21.769,114.383)	10.539<br>(3.769,17.467)	5.905<br>(2.150,10.014)	32.918<br>(11.596,55.248)	18.436<br>(6.617,31.722)
Bahrain	68.174<br>(27.627,119.591)	49.546<br>(19.580,85.444)	12.629<br>(5.166,21.848)	9.157<br>(3.661,15.613)	88.791<br>(35.730,157.020)	64.547<br>(25.326,112.420)

	Best Potential Scenario		Proportional Increase Scenario		Uniform Increase Scenario	
location	Female	Male	Female	Male	Female	Male
Bangladesh	111.909 (36.125,206.234)	67.238 (21.451,123.507)	18.323 (6.184,32.190)	10.991 (3.670,19.267)	39.258 (13.092,69.772)	23.540 (7.770,41.811)
Barbados	18.830 (7.061,31.631)	11.180 (4.132,18.616)	9.210 (3.472,15.421)	5.473 (2.032,9.076)	19.415 (7.235,32.771)	11.519 (4.234,19.288)
Belarus	98.034 (31.484,168.782)	66.267 (21.327,113.544)	31.136 (10.195,52.736)	21.071 (6.906,35.495)	65.137 (21.074,111.448)	44.053 (14.275,74.992)
Belgium	96.819 (32.495,162.694)	59.175 (18.503,99.095)	54.348 (18.318,91.108)	33.220 (10.427,55.426)	85.561 (28.601,144.545)	52.309 (16.291,87.969)
Belize	35.913 (13.473,64.293)	23.371 (8.598,42.071)	18.658 (7.012,33.363)	12.145 (4.475,21.831)	30.636 (11.413,55.288)	19.923 (7.282,36.210)
Benin	138.530 (44.663,250.141)	94.875 (32.020,169.107)	15.954 (5.464,27.136)	10.942 (3.905,18.399)	40.899 (13.812,70.693)	28.043 (9.878,47.755)
Bhutan	18.339 (6.538,31.625)	13.579 (4.938,23.890)	14.790 (5.283,25.444)	10.950 (3.991,19.222)	31.586 (11.154,54.970)	23.377 (8.424,41.517)
Bolivia (Plurinational State of)	329.857 (99.739,579.892)	178.770 (48.089,319.110)	20.871 (6.938,34.112)	11.313 (3.338,18.607)	63.359 (20.717,104.817)	34.299 (9.970,57.317)
Bosnia and Herzegovina	62.649 (19.624,107.195)	27.535 (8.884,49.225)	18.793 (6.068,31.404)	8.267 (2.744,14.431)	31.609 (10.099,53.237)	13.896 (4.569,24.459)
Botswana	94.024 (28.428,165.182)	67.634 (21.096,120.561)	20.289 (6.301,34.512)	14.589 (4.677,25.216)	55.807 (17.086,96.515)	40.088 (12.680,70.390)
Brazil	414.840 (127.531,789.919)	208.417 (63.938,395.030)	35.998 (12.240,62.266)	18.039 (6.115,31.008)	73.039 (24.534,127.678)	36.602 (12.263,63.671)
Brunei Darussalam	65.232 (20.937,115.388)	39.106 (12.458,70.678)	16.130 (5.390,27.453)	9.686 (3.212,16.903)	24.777 (8.216,42.445)	14.880 (4.894,26.118)

	Best Potential Scenario		Proportional Increase Scenario		Uniform Increase Scenario	
**location**	**Female**	**Male**	**Female**	**Male**	**Female**	**Male**
Bulgaria	133.237<br>(43.084,236.012)	62.480<br>(20.519,111.915)	22.376<br>(7.578,37.919)	10.474<br>(3.606,17.913)	45.189<br>(15.139,77.294)	21.142<br>(7.203,36.614)
Burkina Faso	57.729<br>(19.874,100.362)	45.508<br>(15.667,78.238)	5.781<br>(2.077,9.571)	4.556<br>(1.641,7.497)	24.151<br>(8.543,40.741)	19.032<br>(6.743,31.886)
Burundi	44.774<br>(11.960,84.186)	31.004<br>(8.563,58.704)	5.723<br>(1.629,10.124)	3.972<br>(1.162,7.021)	12.372<br>(3.474,22.141)	8.581<br>(2.480,15.402)
Cabo Verde	34.922<br>(11.757,57.899)	28.313<br>(9.697,46.213)	25.544<br>(8.275,43.677)	20.682<br>(6.836,34.966)	62.519<br>(19.973,108.362)	50.588<br>(16.511,86.644)
Cambodia	43.983<br>(15.376,80.505)	36.591<br>(12.637,67.598)	3.806<br>(1.421,6.487)	3.168<br>(1.167,5.448)	11.204<br>(4.127,19.427)	9.333<br>(3.388,16.309)
Cameroon	199.501<br>(72.087,349.932)	134.653<br>(49.914,238.021)	17.032<br>(6.556,28.221)	11.545<br>(4.544,19.085)	51.146<br>(19.427,85.671)	34.630<br>(13.462,58.120)
Canada	265.066<br>(79.017,485.824)	150.686<br>(48.244,272.319)	26.060<br>(8.332,44.424)	14.796<br>(5.088,24.974)	61.692<br>(19.473,106.503)	35.047<br>(11.891,59.825)
Central African Republic	202.438<br>(62.147,372.339)	164.422<br>(48.981,295.629)	22.741<br>(7.514,39.331)	18.525<br>(5.919,31.133)	49.178<br>(16.046,85.923)	40.019<br>(12.641,67.979)
Chad	119.641<br>(30.598,219.120)	78.626<br>(20.926,144.279)	7.446<br>(2.099,12.579)	4.900<br>(1.433,8.228)	25.359<br>(7.023,43.274)	16.678<br>(4.797,28.478)
Chile	339.970<br>(101.053,616.994)	167.728<br>(49.911,305.147)	29.294<br>(9.342,50.424)	14.503<br>(4.619,24.913)	87.813<br>(27.584,152.700)	43.475<br>(13.635,75.707)
China	49.684<br>(16.451,85.710)	29.245<br>(9.373,51.336)	11.439<br>(3.879,19.307)	6.722<br>(2.210,11.555)	28.819<br>(9.649,49.234)	16.972<br>(5.497,29.535)
Colombia	188.366<br>(51.822,368.899)	183.222<br>(51.924,347.304)	13.121<br>(4.018,22.777)	12.626<br>(4.047,21.589)	29.759<br>(9.001,52.438)	28.667<br>(9.059,49.815)

	Best Potential Scenario		Proportional Increase Scenario		Uniform Increase Scenario	
**location**	**Female**	**Male**	**Female**	**Male**	**Female**	**Male**
Congo	451.161 (133.740,865.536)	335.504 (95.955,628.550)	24.882 (8.235,43.004)	18.433 (5.885,31.261)	78.808 (25.668,138.140)	58.354 (18.358,100.419)
Costa Rica	215.174 (59.772,378.765)	145.008 (37.707,257.989)	28.680 (8.538,47.699)	19.334 (5.383,32.632)	54.860 (16.126,92.136)	36.953 (10.167,62.985)
Croatia	37.716 (13.730,65.304)	20.542 (7.689,36.795)	21.258 (7.785,36.548)	11.580 (4.360,20.600)	36.074 (13.098,62.663)	19.646 (7.334,35.301)
Cuba	173.417 (54.196,308.174)	111.797 (33.660,200.828)	34.065 (11.124,57.914)	22.019 (6.909,37.814)	57.515 (18.600,98.754)	37.165 (11.552,64.443)
Cyprus	48.953 (16.603,86.046)	24.820 (8.532,43.439)	16.230 (5.588,28.108)	8.232 (2.870,14.204)	45.250 (15.353,79.513)	22.941 (7.889,40.139)
Czechia	41.487 (14.698,70.016)	20.791 (7.177,35.308)	27.647 (9.808,46.616)	13.855 (4.789,23.505)	45.293 (15.940,76.940)	22.697 (7.781,38.768)
CÔte d'Ivoire	105.592 (32.829,183.420)	72.082 (22.233,126.520)	13.645 (4.417,23.009)	9.317 (2.997,15.738)	38.122 (12.136,65.089)	26.033 (8.229,44.685)
Democratic People's Republic of Korea	72.181 (27.335,126.630)	39.426 (14.658,69.018)	9.786 (3.865,16.426)	5.345 (2.073,8.993)	28.351 (11.042,48.222)	15.481 (5.923,26.350)
Democratic Republic of the Congo	157.156 (44.537,274.844)	124.063 (37.199,220.673)	24.716 (7.356,41.423)	19.547 (6.136,33.273)	50.485 (14.838,85.431)	39.922 (12.381,68.768)
Denmark	136.631 (50.017,240.804)	72.072 (28.016,127.022)	40.499 (15.136,69.844)	21.351 (8.474,36.770)	69.510 (25.759,120.868)	36.658 (14.424,63.782)
Dominica	98.788 (37.844,178.666)	57.424 (21.638,103.315)	29.130 (11.412,51.308)	16.897 (6.508,29.512)	43.981 (17.111,77.927)	25.538 (9.768,44.977)
Dominican Republic	389.013 (138.501,738.773)	221.202 (75.582,417.617)	35.791 (13.964,62.294)	20.444 (7.603,34.912)	70.772 (27.315,124.533)	40.405 (14.876,69.778)

	Best Potential Scenario		Proportional Increase Scenario		Uniform Increase Scenario	
location	Female	Male	Female	Male	Female	Male
Ecuador	319.800<br>(95.398,614.852)	204.000<br>(57.467,394.337)	18.832<br>(6.276,32.347)	11.992<br>(3.778,20.787)	51.358<br>(16.864,89.124)	32.685<br>(10.151,57.444)
Egypt	154.014<br>(53.850,276.994)	91.425<br>(31.532,160.828)	10.495<br>(3.916,17.623)	6.245<br>(2.305,10.314)	37.667<br>(13.830,64.224)	22.365<br>(8.131,37.530)
El Salvador	227.121<br>(69.441,408.033)	135.449<br>(40.295,243.288)	36.004<br>(11.772,61.186)	21.495<br>(6.826,36.417)	56.898<br>(18.444,97.442)	33.995<br>(10.695,57.984)
Equatorial Guinea	231.085<br>(78.800,417.583)	176.381<br>(61.806,333.933)	34.900<br>(12.437,60.503)	26.707<br>(9.786,47.787)	85.936<br>(30.274,151.183)	65.777<br>(23.799,119.126)
Eritrea	82.903<br>(27.911,143.140)	60.816<br>(20.896,107.382)	9.031<br>(3.153,15.012)	6.610<br>(2.367,11.224)	35.622<br>(12.277,60.119)	26.173<br>(9.206,45.092)
Estonia	117.608<br>(34.697,202.958)	76.610<br>(23.185,132.347)	24.807<br>(7.598,41.419)	16.186<br>(5.071,27.081)	49.915<br>(15.106,84.168)	32.544<br>(10.086,54.987)
Eswatini	54.150<br>(17.142,97.832)	42.919<br>(12.614,77.945)	12.588<br>(4.134,21.877)	9.953<br>(3.052,17.273)	21.046<br>(6.856,36.886)	16.648<br>(5.058,29.185)
Ethiopia	77.516<br>(24.979,137.198)	67.258<br>(21.912,118.118)	7.900<br>(2.737,13.149)	6.862<br>(2.401,11.233)	18.398<br>(6.295,30.960)	15.984<br>(5.524,26.558)
Fiji	64.464<br>(19.928,116.956)	49.963<br>(16.138,89.401)	16.666<br>(5.352,29.093)	12.926<br>(4.332,22.251)	25.660<br>(8.181,45.119)	19.904<br>(6.622,34.533)
Finland	33.763<br>(9.634,58.235)	21.045<br>(6.413,36.172)	36.407<br>(10.358,62.955)	22.690<br>(6.894,39.090)	79.350<br>(22.299,138.840)	49.443<br>(14.838,86.075)
France	143.971<br>(50.173,249.755)	80.547<br>(26.662,140.949)	44.341<br>(15.692,75.684)	24.787<br>(8.339,42.484)	75.747<br>(26.572,130.477)	42.364<br>(14.118,73.561)
Gabon	410.150<br>(138.754,764.196)	356.004<br>(121.639,686.978)	36.762<br>(13.460,63.216)	32.059<br>(11.734,56.636)	94.539<br>(34.138,164.449)	82.311<br>(29.788,147.618)

location	Best Potential Scenario		Proportional Increase Scenario		Uniform Increase Scenario	
	Female	Male	Female	Male	Female	Male
Gambia	176.467 (56.667,312.554)	65.564 (20.172,116.239)	31.318 (10.380,53.758)	11.600 (3.701,19.919)	87.187 (28.503,151.702)	32.332 (10.155,56.290)
Georgia	154.417 (51.141,280.044)	111.943 (38.659,195.535)	16.999 (6.008,28.764)	12.285 (4.513,20.395)	38.476 (13.425,65.988)	27.838 (10.097,46.588)
Germany	30.965 (12.856,51.156)	19.774 (7.880,33.041)	35.690 (14.724,59.254)	22.774 (9.027,38.252)	56.216 (23.016,93.886)	35.825 (14.112,60.540)
Ghana	181.658 (72.538,330.121)	129.920 (52.380,236.193)	17.465 (7.375,29.664)	12.505 (5.344,21.118)	47.851 (19.980,82.195)	34.265 (14.456,58.841)
Greece	291.739 (102.350,510.421)	169.118 (60.917,293.521)	41.057 (15.121,68.126)	23.825 (8.990,38.965)	93.885 (34.176,157.764)	54.454 (20.325,90.356)
Grenada	47.627 (13.560,85.366)	30.829 (8.809,54.106)	16.507 (4.831,28.715)	10.701 (3.140,18.242)	22.382 (6.512,39.178)	14.510 (4.233,24.844)
Guatemala	237.882 (88.823,451.421)	130.707 (46.134,247.838)	23.329 (9.489,40.798)	12.868 (4.935,22.097)	43.970 (17.720,77.634)	24.266 (9.214,42.098)
Guinea	95.608 (29.237,178.750)	58.255 (18.138,109.905)	11.360 (3.647,19.986)	6.918 (2.260,12.459)	31.582 (10.000,56.562)	19.235 (6.199,35.077)
Guinea-Bissau	161.591 (53.379,297.561)	96.811 (32.788,180.577)	15.173 (5.393,25.887)	9.110 (3.303,15.774)	40.244 (14.116,69.613)	24.151 (8.650,42.401)
Guyana	87.783 (28.582,154.030)	56.003 (18.881,97.564)	14.285 (4.903,23.980)	9.118 (3.232,15.202)	28.535 (9.673,48.314)	18.211 (6.382,30.623)
Haiti	219.940 (70.607,405.005)	125.151 (42.747,234.272)	19.977 (7.005,33.683)	11.378 (4.254,19.399)	41.996 (14.563,71.694)	23.891 (8.838,41.207)
Honduras	189.814 (61.387,357.324)	107.831 (32.890,200.271)	22.712 (7.935,39.741)	12.869 (4.226,22.251)	40.927 (14.147,72.172)	23.174 (7.540,40.440)

	Best Potential Scenario		Proportional Increase Scenario		Uniform Increase Scenario	
**location**	**Female**	**Male**	**Female**	**Male**	**Female**	**Male**
Hungary	36.223 (11.941,63.193)	19.456 (6.166,32.817)	23.404 (7.707,40.865)	12.571 (3.980,21.230)	41.118 (13.404,72.370)	22.102 (6.923,37.706)
Iceland	153.060 (47.562,273.456)	83.314 (26.440,152.910)	21.586 (7.021,36.720)	11.769 (3.903,20.565)	60.200 (19.309,103.986)	32.800 (10.734,58.179)
India	110.599 (36.567,200.956)	83.273 (27.131,149.343)	11.456 (4.093,19.436)	8.622 (3.029,14.503)	25.249 (8.914,43.331)	19.005 (6.595,32.280)
Indonesia	128.410 (38.511,241.822)	94.921 (28.640,173.846)	10.969 (3.628,18.747)	8.096 (2.705,13.553)	22.063 (7.213,38.122)	16.288 (5.377,27.532)
Iran (Islamic Republic of)	379.990 (120.598,711.160)	225.067 (70.758,416.974)	19.038 (6.549,32.683)	11.293 (3.846,19.223)	85.232 (28.837,148.992)	50.654 (16.936,87.638)
Iraq	154.287 (51.368,282.705)	97.221 (30.868,177.790)	11.161 (3.900,19.417)	7.047 (2.344,12.259)	63.361 (21.731,112.700)	39.985 (13.059,70.920)
Ireland	106.220 (34.235,189.500)	67.211 (22.874,118.605)	43.722 (14.290,76.969)	27.713 (9.554,48.063)	62.711 (20.363,110.997)	39.714 (13.611,69.452)
Israel	43.850 (14.925,74.760)	26.061 (9.075,43.753)	32.700 (11.098,55.836)	19.439 (6.750,32.682)	88.769 (29.688,152.977)	52.841 (18.074,90.166)
Italy	102.657 (33.702,181.863)	53.519 (18.011,91.184)	35.273 (11.773,61.311)	18.366 (6.292,30.894)	65.390 (21.616,114.956)	34.074 (11.552,57.693)
Jamaica	83.837 (26.235,150.281)	45.401 (14.350,80.900)	20.977 (6.767,36.293)	11.365 (3.703,19.642)	34.724 (11.119,60.930)	18.829 (6.084,32.806)
Japan	278.119 (93.565,523.917)	200.154 (67.703,381.442)	16.083 (6.048,27.078)	11.532 (4.393,19.718)	39.767 (14.773,67.802)	28.530 (10.725,49.371)
Jordan	211.652 (69.546,375.889)	109.983 (35.177,197.726)	18.022 (6.198,30.650)	9.371 (3.149,16.016)	82.808 (28.006,142.915)	43.067 (14.205,75.104)

	Best Potential Scenario		Proportional Increase Scenario		Uniform Increase Scenario	
**location**	**Female**	**Male**	**Female**	**Male**	**Female**	**Male**
Kazakhstan	51.566<br>(18.865,88.494)	33.681<br>(12.368,58.961)	11.301<br>(4.198,19.013)	7.379<br>(2.755,12.684)	39.303<br>(14.393,67.367)	25.669<br>(9.437,44.893)
Kenya	128.152<br>(36.579,230.606)	98.868<br>(28.807,177.544)	12.415<br>(3.870,20.611)	9.574<br>(3.053,16.024)	25.205<br>(7.768,42.200)	19.442<br>(6.127,32.824)
Kuwait	9.650<br>(2.932,16.804)	6.452<br>(1.978,11.022)	10.351<br>(3.138,18.076)	6.922<br>(2.117,11.851)	83.639<br>(24.906,148.955)	55.934<br>(16.778,97.801)
Kyrgyzstan	54.494<br>(15.050,96.596)	35.223<br>(10.384,62.786)	8.350<br>(2.400,14.388)	5.402<br>(1.656,9.314)	27.038<br>(7.647,47.193)	17.469<br>(5.277,30.618)
Lao People's Democratic Republic	60.376<br>(21.558,111.379)	64.471<br>(22.819,120.798)	6.012<br>(2.317,10.243)	6.426<br>(2.446,11.002)	13.231<br>(5.047,22.801)	14.139<br>(5.331,24.445)
Latvia	68.024<br>(20.225,116.719)	49.311<br>(15.485,85.272)	26.465<br>(8.006,44.627)	19.169<br>(6.129,32.747)	48.922<br>(14.644,83.365)	35.454<br>(11.211,60.994)
Lebanon	158.690<br>(53.080,275.233)	100.941<br>(34.451,182.347)	32.725<br>(11.200,55.460)	20.776<br>(7.267,36.826)	90.015<br>(30.376,154.643)	57.181<br>(19.713,102.442)
Lesotho	37.631<br>(14.704,65.535)	25.709<br>(9.868,45.307)	14.527<br>(5.746,24.898)	9.920<br>(3.858,17.234)	32.588<br>(12.765,56.572)	22.262<br>(8.568,39.120)
Liberia	226.059<br>(70.868,416.184)	91.785<br>(27.305,163.472)	23.622<br>(7.970,40.146)	9.594<br>(3.083,15.832)	55.142<br>(18.372,94.888)	22.407<br>(7.102,37.369)
Libya	46.016<br>(15.882,79.764)	32.446<br>(11.333,56.002)	18.026<br>(6.229,31.216)	12.711<br>(4.445,21.913)	74.567<br>(25.330,131.054)	52.530<br>(18.075,92.233)
Lithuania	102.197<br>(31.409,183.781)	70.651<br>(21.429,128.048)	29.917<br>(9.425,52.605)	20.678<br>(6.430,36.742)	56.412<br>(17.582,100.212)	39.004<br>(11.995,69.999)
Luxembourg	90.921<br>(30.487,154.973)	51.544<br>(17.335,89.906)	44.089<br>(14.965,74.257)	24.993<br>(8.515,43.090)	68.173<br>(22.968,115.741)	38.643<br>(13.063,67.130)

	Best Potential Scenario		Proportional Increase Scenario		Uniform Increase Scenario	
location	Female	Male	Female	Male	Female	Male
Madagascar	165.255<br>(43.836,309.466)	118.961<br>(32.347,220.696)	15.150<br>(4.349,25.830)	10.832<br>(3.210,18.486)	35.528<br>(10.071,61.276)	25.418<br>(7.433,43.834)
Malawi	36.809<br>(11.997,66.780)	26.357<br>(8.302,48.455)	5.810<br>(1.990,10.028)	4.171<br>(1.375,7.220)	12.794<br>(4.330,22.435)	9.186<br>(2.993,16.146)
Malaysia	185.388<br>(54.832,345.774)	163.250<br>(49.876,299.528)	21.640<br>(6.904,37.349)	18.957<br>(6.292,32.387)	41.443<br>(13.072,72.298)	36.319<br>(11.911,62.600)
Mali	89.443<br>(30.914,156.788)	56.727<br>(20.018,100.947)	8.905<br>(3.249,14.751)	5.659<br>(2.102,9.536)	29.829<br>(10.719,50.201)	18.964<br>(6.936,32.352)
Malta	139.189<br>(50.223,235.354)	71.900<br>(25.478,120.772)	22.369<br>(8.318,36.792)	11.566<br>(4.225,18.946)	67.829<br>(24.867,112.912)	35.059<br>(12.624,58.154)
Mauritania	80.545<br>(30.079,142.375)	64.951<br>(24.971,113.914)	4.254<br>(1.659,7.180)	3.425<br>(1.376,5.773)	35.407<br>(13.559,61.021)	28.540<br>(11.247,48.875)
Mauritius	36.081<br>(13.138,63.404)	22.329<br>(8.350,38.400)	19.263<br>(7.046,33.694)	11.914<br>(4.477,20.429)	30.859<br>(11.200,54.434)	19.107<br>(7.119,32.923)
Mexico	591.026<br>(166.955,1163.936)	306.411<br>(83.920,582.768)	27.181<br>(8.852,46.128)	14.060<br>(4.440,23.500)	73.805<br>(23.672,127.243)	38.211<br>(11.877,64.733)
Mongolia	64.942<br>(21.293,114.533)	45.442<br>(13.892,80.132)	9.799<br>(3.296,16.833)	6.858<br>(2.150,11.813)	49.564<br>(16.353,86.912)	34.707<br>(10.669,60.851)
Montenegro	73.796<br>(25.356,128.315)	34.349<br>(11.578,58.961)	22.453<br>(7.872,38.281)	10.467<br>(3.592,17.644)	41.579<br>(14.426,71.607)	19.367<br>(6.585,32.977)
Morocco	122.549<br>(40.431,217.618)	83.738<br>(25.349,151.784)	20.855<br>(7.061,36.104)	14.279<br>(4.438,25.282)	74.624<br>(24.851,131.224)	51.015<br>(15.595,91.661)
Mozambique	101.446<br>(35.993,185.780)	79.049<br>(28.454,144.661)	14.539<br>(5.440,25.197)	11.366<br>(4.289,19.736)	33.176<br>(12.262,58.133)	25.939<br>(9.674,45.639)

	Best Potential Scenario		Proportional Increase Scenario		Uniform Increase Scenario	
location	Female	Male	Female	Male	Female	Male
Myanmar	17.471<br>(5.633,29.326)	15.940<br>(5.107,27.367)	4.960<br>(1.639,8.127)	4.510<br>(1.485,7.597)	9.655<br>(3.159,15.973)	8.794<br>(2.863,14.929)
Namibia	107.915<br>(36.885,185.572)	84.414<br>(28.833,149.376)	8.971<br>(3.243,14.641)	7.003<br>(2.533,11.888)	35.706<br>(12.675,59.342)	27.867<br>(9.904,47.957)
Nepal	40.504<br>(14.750,67.322)	28.523<br>(10.551,47.949)	9.018<br>(3.365,14.536)	6.343<br>(2.409,10.358)	22.637<br>(8.354,37.003)	15.932<br>(5.978,26.353)
Netherlands	61.325<br>(25.232,101.192)	36.425<br>(14.581,60.309)	48.150<br>(19.793,79.543)	28.601<br>(11.439,47.407)	77.987<br>(31.830,129.785)	46.347<br>(18.393,77.554)
New Zealand	95.221<br>(29.466,164.098)	62.378<br>(20.506,104.098)	40.899<br>(12.843,69.530)	26.778<br>(8.944,44.198)	59.929<br>(18.691,102.541)	39.232<br>(13.013,65.090)
Nicaragua	164.690<br>(58.085,301.202)	101.256<br>(35.666,190.884)	22.853<br>(8.601,39.424)	14.091<br>(5.276,24.775)	42.410<br>(15.797,73.784)	26.143<br>(9.692,46.466)
Niger	13.255<br>(5.075,21.949)	8.061<br>(3.092,13.352)	3.362<br>(1.303,5.487)	2.043<br>(0.794,3.340)	13.900<br>(5.305,23.093)	8.452<br>(3.232,14.045)
Nigeria	141.675<br>(41.698,253.783)	99.051<br>(28.539,176.670)	11.798<br>(3.736,19.864)	8.225<br>(2.545,13.698)	35.940<br>(11.202,61.299)	25.069<br>(7.639,42.428)
North Macedonia	124.701<br>(39.120,228.730)	57.155<br>(17.994,104.861)	16.599<br>(5.555,28.421)	7.621<br>(2.546,13.132)	35.198<br>(11.629,60.980)	16.154<br>(5.334,28.145)
Norway	209.962<br>(71.937,375.158)	137.650<br>(44.349,241.446)	36.624<br>(13.198,62.657)	23.986<br>(8.148,40.079)	65.924<br>(23.529,113.932)	43.175<br>(14.519,72.773)
Oman	108.814<br>(36.045,184.119)	73.936<br>(24.375,126.489)	14.886<br>(5.032,24.755)	10.114<br>(3.405,16.942)	77.345<br>(25.702,130.433)	52.545<br>(17.384,89.534)
Pakistan	110.418<br>(36.282,200.511)	75.694<br>(25.218,135.229)	9.462<br>(3.321,16.181)	6.520<br>(2.308,11.017)	30.839<br>(10.644,53.482)	21.239<br>(7.398,36.344)

location	Best Potential Scenario		Proportional Increase Scenario		Uniform Increase Scenario	
	Female	Male	Female	Male	Female	Male
Palestine	74.698 (27.739,128.340)	68.877 (25.481,118.892)	29.969 (11.204,51.110)	27.628 (10.295,47.369)	92.613 (34.180,160.683)	85.504 (31.390,148.774)
Panama	191.502 (62.222,353.308)	119.195 (39.512,220.144)	22.647 (7.924,39.233)	14.184 (4.999,24.253)	47.430 (16.372,83.060)	29.660 (10.342,51.329)
Papua New Guinea	23.594 (7.551,42.775)	19.412 (6.237,35.428)	3.569 (1.214,6.088)	2.930 (1.002,5.055)	6.062 (2.043,10.449)	4.976 (1.686,8.671)
Paraguay	146.970 (48.921,253.676)	62.001 (21.267,109.182)	27.696 (9.639,46.086)	11.661 (4.200,19.628)	47.954 (16.538,80.474)	20.194 (7.203,34.313)
Peru	323.691 (98.280,640.400)	185.752 (59.619,378.926)	9.244 (3.229,15.938)	5.300 (1.969,9.309)	38.543 (13.237,67.597)	22.117 (8.068,39.431)
Philippines	115.936 (32.799,215.089)	99.373 (27.378,178.212)	11.334 (3.500,19.479)	9.748 (2.918,16.145)	23.625 (7.196,41.066)	20.303 (6.000,33.978)
Poland	37.833 (12.031,64.057)	30.709 (9.290,51.962)	12.992 (4.217,21.704)	10.544 (3.261,17.575)	22.753 (7.314,38.308)	18.475 (5.652,31.012)
Portugal	291.418 (88.211,499.740)	136.655 (40.096,236.861)	41.494 (13.345,67.371)	19.421 (6.081,31.910)	79.100 (25.151,129.694)	36.989 (11.456,61.441)
Puerto Rico	125.265 (40.212,217.891)	88.835 (29.114,155.685)	35.400 (11.688,60.225)	25.107 (8.458,42.732)	49.890 (16.365,85.316)	35.411 (11.844,60.560)
Qatar	15.650 (5.552,27.427)	11.086 (3.843,19.094)	9.521 (3.374,16.705)	6.743 (2.335,11.625)	86.132 (29.951,155.134)	60.992 (20.740,107.271)
Republic of Korea	29.046 (9.410,49.516)	18.865 (6.232,31.771)	14.149 (4.603,24.045)	9.194 (3.048,15.413)	34.970 (11.214,60.098)	22.692 (7.429,38.661)
Republic of Moldova	21.349 (7.298,35.176)	14.456 (5.101,23.525)	13.724 (4.705,22.554)	9.290 (3.288,15.075)	28.299 (9.607,46.999)	19.188 (6.716,31.451)

	Best Potential Scenario		Proportional Increase Scenario		Uniform Increase Scenario	
location	Female	Male	Female	Male	Female	Male
Romania	40.665<br>(13.992,66.758)	21.447<br>(7.044,35.877)	15.225<br>(5.296,24.769)	8.026<br>(2.666,13.321)	31.700<br>(10.902,52.075)	16.718<br>(5.489,27.988)
Russian Federation	66.410<br>(20.200,109.736)	53.833<br>(16.494,86.122)	17.908<br>(5.552,29.027)	14.504<br>(4.534,22.878)	44.754<br>(13.684,73.567)	36.271<br>(11.174,57.798)
Rwanda	47.191<br>(16.444,81.717)	26.343<br>(9.405,44.989)	9.495<br>(3.428,15.857)	5.294<br>(1.964,8.734)	18.324<br>(6.551,30.902)	10.214<br>(3.753,17.018)
Saint Kitts and Nevis	47.449<br>(16.999,83.995)	27.777<br>(9.571,49.320)	15.231<br>(5.602,26.332)	8.936<br>(3.146,15.537)	25.125<br>(9.156,43.864)	14.741<br>(5.147,25.824)
Saint Lucia	10.824<br>(3.632,18.847)	7.321<br>(2.595,12.653)	7.253<br>(2.449,12.560)	4.910<br>(1.749,8.434)	12.029<br>(4.023,20.994)	8.133<br>(2.876,14.102)
Saint Vincent and the Grenadines	40.904<br>(13.517,67.791)	29.286<br>(10.050,50.406)	23.359<br>(7.792,38.420)	16.744<br>(5.790,28.596)	33.863<br>(11.205,56.056)	24.246<br>(8.330,41.687)
Saudi Arabia	126.835<br>(43.288,217.617)	84.428<br>(28.659,145.783)	9.364<br>(3.297,15.634)	6.248<br>(2.185,10.487)	81.520<br>(28.159,138.498)	54.358<br>(18.642,92.832)
Senegal	122.876<br>(43.631,220.686)	94.366<br>(32.727,167.764)	8.773<br>(3.293,14.667)	6.693<br>(2.472,11.286)	36.410<br>(13.477,61.952)	27.851<br>(10.115,47.599)
Serbia	27.907<br>(8.705,49.327)	13.658<br>(4.259,23.836)	18.084<br>(5.666,31.829)	8.850<br>(2.773,15.385)	33.967<br>(10.521,60.407)	16.619<br>(5.147,29.176)
Sierra Leone	106.279<br>(31.755,187.557)	62.700<br>(19.037,108.340)	14.955<br>(4.646,25.624)	8.836<br>(2.776,14.794)	39.174<br>(11.986,67.935)	23.123<br>(7.171,39.242)
Singapore	12.840<br>(4.741,21.744)	8.959<br>(3.418,15.215)	17.901<br>(6.546,30.548)	12.487<br>(4.720,21.382)	40.231<br>(14.528,69.311)	28.059<br>(10.476,48.569)
Slovakia	46.733<br>(16.567,82.192)	21.234<br>(7.003,37.620)	18.078<br>(6.504,31.228)	8.195<br>(2.748,14.297)	31.120<br>(11.102,54.219)	14.128<br>(4.692,24.864)

	Best Potential Scenario		Proportional Increase Scenario		Uniform Increase Scenario	
location	Female	Male	Female	Male	Female	Male
Slovenia	104.507<br>(36.012,189.226)	45.413<br>(15.836,81.813)	25.596<br>(9.125,44.717)	11.130<br>(4.017,19.263)	40.763<br>(14.420,71.749)	17.723<br>(6.345,30.948)
Somalia	262.451<br>(73.281,497.576)	174.428<br>(47.921,341.515)	11.413<br>(3.568,19.531)	7.570<br>(2.337,13.093)	44.451<br>(13.653,77.372)	29.486<br>(8.929,52.272)
South Africa	181.357<br>(63.316,318.640)	125.183<br>(45.524,216.315)	27.850<br>(10.175,46.649)	19.154<br>(7.315,31.856)	61.177<br>(22.114,103.817)	42.137<br>(15.899,70.822)
South Sudan	49.576<br>(16.951,89.273)	34.041<br>(11.698,60.189)	10.419<br>(3.698,18.129)	7.150<br>(2.555,12.132)	18.750<br>(6.598,32.935)	12.872<br>(4.557,22.077)
Spain	287.757<br>(100.905,514.617)	132.408<br>(47.723,232.362)	43.547<br>(15.906,74.766)	20.101<br>(7.517,33.972)	99.178<br>(35.769,172.284)	45.711<br>(16.908,78.133)
Sri Lanka	14.078<br>(4.717,24.422)	12.586<br>(4.092,22.203)	5.181<br>(1.770,8.847)	4.639<br>(1.534,8.033)	8.231<br>(2.786,14.143)	7.361<br>(2.415,12.867)
Sudan	86.782<br>(27.158,150.858)	71.837<br>(23.432,127.972)	5.788<br>(1.913,9.660)	4.793<br>(1.649,8.147)	30.229<br>(9.820,51.198)	25.055<br>(8.470,43.313)
Suriname	273.413<br>(92.640,504.651)	173.700<br>(58.799,325.255)	34.712<br>(12.550,59.673)	22.019<br>(7.975,38.805)	65.553<br>(23.469,113.920)	41.582<br>(14.910,73.879)
Sweden	213.727<br>(62.470,368.358)	95.318<br>(28.304,165.680)	50.441<br>(15.258,84.167)	22.515<br>(6.908,38.184)	97.383<br>(29.119,164.280)	43.478<br>(13.186,74.229)
Switzerland	92.097<br>(32.423,165.579)	55.393<br>(19.616,102.346)	38.738<br>(13.842,68.578)	23.293<br>(8.366,42.342)	58.398<br>(20.718,104.233)	35.119<br>(12.527,64.306)
Syrian Arab Republic	198.929<br>(58.277,364.676)	134.135<br>(38.342,244.539)	12.651<br>(4.023,21.402)	8.546<br>(2.647,14.342)	46.512<br>(14.548,80.035)	31.400<br>(9.572,53.440)
Tajikistan	20.064<br>(5.866,34.154)	11.092<br>(3.471,18.944)	5.250<br>(1.564,8.775)	2.903<br>(0.925,4.879)	16.029<br>(4.701,27.204)	8.861<br>(2.781,15.096)

	Best Potential Scenario		Proportional Increase Scenario		Uniform Increase Scenario	
location	Female	Male	Female	Male	Female	Male
Thailand	116.413<br>(36.037,226.846)	92.344<br>(29.863,181.297)	10.005<br>(3.381,17.763)	7.934<br>(2.812,14.195)	22.052<br>(7.363,39.573)	17.481<br>(6.121,31.675)
Timor-Leste	29.990<br>(9.345,52.328)	24.676<br>(7.968,43.872)	5.861<br>(1.894,9.840)	4.813<br>(1.612,8.279)	13.378<br>(4.272,22.768)	10.982<br>(3.637,19.127)
Togo	146.938<br>(53.703,270.360)	101.830<br>(37.503,183.327)	13.321<br>(5.190,22.673)	9.165<br>(3.610,15.465)	36.610<br>(14.068,63.054)	25.195<br>(9.792,43.053)
Trinidad and Tobago	92.062<br>(31.874,163.939)	64.786<br>(21.129,116.478)	22.085<br>(7.895,37.913)	15.538<br>(5.234,27.182)	39.078<br>(13.838,68.005)	27.480<br>(9.173,48.409)
Tunisia	351.207<br>(109.591,669.652)	252.610<br>(77.334,485.759)	21.543<br>(7.338,38.066)	15.506<br>(5.190,27.271)	77.027<br>(25.806,138.188)	55.459<br>(18.243,99.197)
Turkey	437.058<br>(125.199,839.393)	246.667<br>(72.176,454.769)	23.205<br>(7.442,39.782)	13.088<br>(4.297,22.099)	68.188<br>(21.511,118.354)	38.361<br>(12.417,65.673)
Turkmenistan	7.609<br>(2.735,12.520)	4.602<br>(1.683,7.598)	8.656<br>(3.102,14.277)	5.235<br>(1.909,8.665)	33.863<br>(11.933,56.619)	20.472<br>(7.348,34.392)
Uganda	101.893<br>(25.347,182.108)	79.530<br>(20.057,143.501)	18.717<br>(4.919,32.183)	14.613<br>(3.902,25.056)	32.640<br>(8.480,56.476)	25.469<br>(6.722,44.049)
Ukraine	91.663<br>(32.416,165.290)	63.677<br>(21.591,114.629)	27.943<br>(10.097,49.471)	19.472<br>(6.730,34.405)	53.740<br>(19.221,96.079)	37.405<br>(12.804,66.724)
United Arab Emirates	27.451<br>(8.966,46.688)	20.085<br>(6.780,34.463)	10.445<br>(3.428,17.692)	7.639<br>(2.592,13.044)	69.362<br>(22.351,119.214)	50.770<br>(16.908,88.281)
United Kingdom	134.179<br>(35.656,236.889)	99.174<br>(27.862,169.344)	50.546<br>(13.735,87.744)	37.367<br>(10.719,62.856)	78.584<br>(21.157,137.376)	58.051<br>(16.519,98.277)
United Republic of Tanzania	102.532<br>(31.697,183.622)	69.004<br>(20.970,123.278)	15.535<br>(5.074,26.302)	10.432<br>(3.356,17.746)	32.074<br>(10.356,54.956)	21.558<br>(6.849,36.987)

location	Best Potential Scenario		Proportional Increase Scenario		Uniform Increase Scenario	
	Female	Male	Female	Male	Female	Male
United States of America	47.432 (13.619,79.599)	29.997 (8.384,50.290)	46.677 (13.375,78.430)	29.518 (8.234,49.561)	61.880 (17.618,104.388)	39.114 (10.849,66.025)
United States Virgin Islands	393.645 (123.066,719.386)	216.196 (68.600,395.098)	47.099 (15.767,80.336)	25.870 (8.791,44.103)	90.426 (29.947,156.105)	49.714 (16.698,85.740)
Uruguay	72.639 (21.747,126.604)	39.145 (11.974,66.136)	33.346 (10.043,57.665)	17.940 (5.530,30.150)	64.059 (19.105,112.104)	34.561 (10.520,58.616)
Uzbekistan	36.479 (13.209,61.856)	23.477 (8.419,39.672)	10.228 (3.749,17.138)	6.589 (2.390,10.968)	32.635 (11.779,55.519)	20.998 (7.508,35.635)
Venezuela (Bolivarian Republic of)	312.904 (95.512,584.961)	216.452 (65.907,407.252)	24.204 (8.115,40.708)	16.731 (5.630,28.870)	55.880 (18.497,95.277)	38.622 (12.825,67.311)
Viet Nam	64.122 (18.300,117.952)	38.979 (11.505,69.981)	5.395 (1.668,9.263)	3.278 (1.045,5.457)	14.610 (4.451,25.439)	8.871 (2.790,14.976)
Yemen	249.645 (72.967,490.534)	165.909 (47.847,322.226)	7.181 (2.395,12.415)	4.809 (1.563,8.282)	37.658 (12.310,66.442)	25.198 (8.043,44.017)
Zambia	114.086 (37.480,202.219)	90.491 (30.325,166.679)	12.998 (4.524,21.632)	10.299 (3.670,17.771)	32.520 (11.179,54.879)	25.820 (9.064,45.121)
Zimbabwe	37.226 (12.740,64.917)	28.652 (10.089,51.435)	8.406 (2.962,14.223)	6.458 (2.340,11.139)	18.473 (6.437,31.640)	14.199 (5.089,24.868)

Table S7. Gender differences of preventable age-standardised DALY rates of depression attributable to greenness exposure in 2020 under the proportional increase scenario and uniform increase scenario at the global and regional levels.

	Proportional Increase Scenario		Uniform Increase Scenario	
	**Absolute Difference**	**relative difference**	**Absolute Difference**	**relative difference**
Global	6.533<br>(3.468,9.856)	1.588<br>(1.279,1.998)	14.817<br>(7.837,22.415)	1.568<br>(1.270,1.963)
WHO region				
African region	3.908<br>(0.873,7.406)	1.377<br>(1.068,1.782)	10.326<br>(2.367,19.635))	1.388<br>(1.073,1.807)
American region	14.991<br>(1.714,31.774)	1.814<br>(1.072,3.081)	31.610<br>(4.785,64.936)	1.81<br>(1.099,2.995)
Eastern Mediterranean Region	4.292<br>(0.550,8.027)	1.558<br>(1.056,2.199)	17.019<br>(1.991,31.977)	1.511<br>(1.044,2.087)
European Region	10.987<br>(4.782,17.570)	1.601<br>(1.234,2.077)	21.906<br>(9.725,34.836)	1.607<br>(1.240,2.084)
Southeast Asian Region	2.996<br>(-3.860,11.214)	1.362<br>(0.692,2.663)	6.531<br>(-8.936,25.141)	1.363<br>(0.678,2.721)
Western Pacific Region	4.648<br>(-2.830,13.255)	1.612<br>(0.744,3.356)	11.442<br>(1.255, -7.976)	1.624<br>(0.710,3.544)
SDI region				
High SDI	14.421<br>(1.623,30.513)	1.698<br>(1.066,2.723)	26.558<br>(1.774,57.791)	1.646<br>(1.039,2.633)
High-middle SDI	6.541<br>(0.088,14.236)	1.721<br>(1.008,2.811)	15.748<br>(-0.717,35.625)	1.708<br>(0.974,2.858)
Middle SDI	7.137<br>(2.089,12.995)	1.642<br>(1.169,2.322)	17.695<br>(5.968,30.952)	1.635<br>(1.194,2.250)
Low-middle SDI	3.523	1.415	8.797	1.422

	(-2.262,10.219)	(0.797,2.506)	(-4.258,23.885)	(0.838,2.403)
Low SDI	3.437<br>(0.459,6.636)	1.368<br>(1.044,1.826)	9.064<br>(2.026,16.463)	1.377<br>(1.077,1.788)

Table S8. Gender differences of preventable age-standardised DALY rates of depression attributable to greenness exposure from 2001 to 2020 under the three scenarios at the national level.

	Best Potential Scenario		Proportional Increase Scenario		Uniform Increase Scenario	
**location**	**absolute difference**	**relative difference**	**absolute difference**	**relative difference**	**absolute difference**	**relative difference**
Afghanistan	25.874<br>(-84.655,182.386)	1.332<br>(0.400,4.978)	1.381<br>(-4.196,7.764)	1.359<br>(0.433,4.035)	6.862<br>(-21.768,39.684)	1.358<br>(0.421,4.136)
Albania	73.207<br>(-27.093,262.609)	2.358<br>(0.727,7.914)	9.386<br>(-2.333,31.776)	2.438<br>(0.805,7.406)	19.969<br>(-5.201,68.577)	2.442<br>(0.794,7.531)
Algeria	137.5<br>(-329.540,792.567)	1.529<br>(0.427,5.658)	6.576<br>(-11.474,30.192)	1.579<br>(0.494,4.754)	21.623<br>(-39.307,101.635)	1.58<br>(0.482,4.864)
Andorra	30.438<br>(-35.257,117.747)	1.688<br>(0.550,5.423)	20.607<br>(-21.088,81.257)	1.699<br>(0.588,5.242)	32.102<br>(-33.860,128.044)	1.696<br>(0.581,5.286)
Angola	72.711<br>(-432.486,725.915)	1.233<br>(0.344,4.865)	4.061<br>(-13.628,29.102)	1.282<br>(0.466,4.406)	17.446<br>(-60.789,128.458)	1.28<br>(0.456,4.506)
Argentina	77.876<br>(-139.151,385.667)	1.616<br>(0.476,5.338)	8.937<br>(-12.905,40.409)	1.647<br>(0.521,5.233)	21.468<br>(-31.741,98.544)	1.648<br>(0.513,5.326)
Armenia	46.389<br>(90.458,233.079)	1.564<br>(0.452,5.334)	4.237<br>(-7.876,20.993)	1.493<br>(0.473,5.017)	12.399<br>(-23.950,63.049)	1.492<br>(0.463,5.125)

	Best Potential Scenario		Proportional Increase Scenario		Uniform Increase Scenario	
location	absolute difference	relative difference	absolute difference	relative difference	absolute difference	relative difference
Australia	98.379	1.408	13.203	1.43	23.676	1.43
	(-227.827,588.181)	(0.459,4.582)	(-25.750,64.756)	(0.472,4.269)	(-46.972,117.258)	(0.433,4.330)
Austria	50.924	1.911	10.283	1.929	18.505	1.932
	(-43.930,196.056)	(0.594,6.084)	(-7.471,39.102)	(0.665,6.024)	(-13.822,71.094)	(0.658,6.118)
Azerbaijan	49.678	1.843	4.824	1.847	15.073	1.849
	(-56.531,200.366)	(0.562,6.029)	(-4.041,17.419)	(0.626,4.854)	(-13.172,55.511)	(0.614,4.949)
Bahrain	17.876	1.364	3.772	1.449	26.32	1.448
	(-51.827,94.896)	(0.450,3.917)	(-8.043,17.828)	(0.472,3.869)	(-59.307,128.653)	(0.458,3.970)
Bangladesh	42.435	1.648	6.909	1.691	14.795	1.692
	(-67.716,209.639)	(0.486,5.794)	(-9.729,33.012)	(0.513,5.549)	(-21.410,71.955)	(0.504,5.649)
Barbados	8.111	1.729	3.661	1.662	7.731	1.664
	(-7.398,31.688)	(0.633,4.627)	(-4.949,13.867)	(0.531,5.060)	(-10.668,29.614)	(0.524,5.141)
Belarus	27.145	1.442	8.648	1.429	18.065	1.429
	(-67.178,169.460)	(0.437,5.113)	(-21.393,47.421)	(0.497,4.087)	(-46.048,100.586)	(0.490,4.145)
Belgium	37.762	1.649	18.898	1.572	29.684	1.571
	(-53.320,172.851)	(0.512,5.891)	(-29.414,91.090)	(0.554,5.216)	(-47.222,144.935)	(0.548,5.319)

	Best Potential Scenario		Proportional Increase Scenario		Uniform Increase Scenario	
location	absolute difference	relative difference	absolute difference	relative difference	absolute difference	relative difference
Belize	11.533<br>(-20.543,57.419)	1.524<br>(0.475,4.470)	6.544<br>(-10.659,28.824)	1.581<br>(0.517,4.331)	10.762<br>(-17.796, 47.979)	1.583<br>(0.510,4.388)
Benin	43.255<br>(-121.597,236.043)	1.472<br>(0.416,4.581)	5.278<br>(-10.332,26.186)	1.51<br>(0.487,4.492)	13.555<br>(-27.303,68.716)	1.511<br>(0.478,4.581)
Bhutan	4.348<br>(-14.280,28.666)	1.323<br>(0.449,4.075)	3.46<br>(-10.589,21.254)	1.331<br>(0.428,4.227)	7.386<br>(-23.200,46.167)	1.332<br>(0.421,4.300)
Bolivia (Plurinational State of)	141.966<br>(-179.559,591.178)	1.848<br>(0.489,6.290)	9.612<br>(-8.625,34.755)	1.867<br>(0.576,6.020)	29.231<br>(-27.002,107.440)	1.869<br>(0.565,6.158)
Bosnia and Herzegovina	33.067<br>(-16.295,121.023)	2.169<br>(0.699,8.160)	9.713<br>(-5.579,32.416)	2.198<br>(0.639,6.944)	16.321<br>(-9.583,55.509)	2.199<br>(0.631,7.035)
Botswana	26.711<br>(-71.465,148.565)	1.432<br>(0.436,4.362)	5.676<br>(-16.493,32.952)	1.415<br>(0.424,4.663)	16.657<br>(-46.310,92.950)	1.417<br>(0.415,4.769)
Brazil	197.614<br>(-180.842,859.086)	2.033<br>(0.549,7.373)	17.209<br>(-13.852,59.236)	1.979<br>(0.655,5.927)	34.888<br>(-29.590,122.028)	1.978<br>(0.644,6.018)
Brunei Darussalam	26.665<br>(-34.629,103.915)	1.707<br>(0.526,5.081)	6.199<br>(-8.114,26.661)	1.669<br>(0.526,4.881)	9.513<br>(-12.722,41.315)	1.669<br>(0.521,4.925)

	Best Potential Scenario		Proportional Increase Scenario		Uniform Increase Scenario	
location	absolute difference	relative difference	absolute difference	relative difference	absolute difference	relative difference
Bulgaria	66.441<br>(-36.427,230.187)	2.073<br>(0.669,6.928)	11.386<br>(-5.661,39.724)	2.15<br>(0.680,6.621)	22.967<br>(-11.915,81.914)	2.151<br>(0.671,6.728)
Burkina Faso	11.088<br>(-47.835,82.615)	1.252<br>(0.407,4.019)	1.048<br>(-4.717,8.189)	1.259<br>(0.436,3.621)	4.364<br>(-20.466,35.053)	1.259<br>(0.426,3.709)
Burundi	15.665<br>(-38.071,90.747)	1.508<br>(0.405,6.238)	1.686<br>(-4.805,10.308)	1.426<br>(0.406,5.073)	3.65<br>(-10.665,22.690)	1.427<br>(0.399,5.171)
Cabo Verde	6.129<br>(-33.728,51.510)	1.233<br>(0.336,3.645)	5.613<br>(-23.704,36.525)	1.296<br>(0.405,3.755)	13.788<br>(-59.371,91.214)	1.298<br>(0.398,3.825)
Cambodia	7.727<br>(-48.012,69.703)	1.224<br>(0.374,4.126)	0.616<br>(-3.026,5.056)	1.203<br>(0.429,3.345)	1.809<br>(-9.113,15.240)	1.202<br>(0.420,3.413)
Cameroon	60.522<br>(-127.729,339.022)	1.489<br>(0.465,4.671)	5.051<br>(-9.580,22.774)	1.47<br>(0.533,3.802)	15.195<br>(-29.712,69.598)	1.473<br>(0.524,3.827)
Canada	117.027<br>(-151.427,529.069)	1.806<br>(0.471,6.682)	10.783<br>(-9.961,44.596)	1.745<br>(0.611,5.556)	25.474<br>(-24.808,107.280)	1.744<br>(0.601,5.562)
Central African Republic	31.258<br>(-226.415,378.179)	1.208<br>(0.339,4.477)	4.354<br>(-21.418,34.289)	1.232<br>(0.420,3.958)	9.446<br>(-47.236,75.589)	1.233<br>(0.414,4.025)

	Best Potential Scenario		Proportional Increase Scenario		Uniform Increase Scenario	
location	absolute difference	relative difference	absolute difference	relative difference	absolute difference	relative difference
Chad	40.399<br>(-98.012,257.884)	1.543<br>(0.384,5.876)	2.523<br>(-4.682,14.630)	1.565<br>(0.456,5.850)	8.574<br>(-16.378,50.792)	1.566<br>(0.447,5.996)
Chile	160.1<br>(-136.555,690.092)	2.003<br>(0.584,7.132)	14.847<br>(-9.596,55.542)	2.022<br>(0.639,6.390)	44.345<br>(-30.532,169.462)	2.022<br>(0.627,6.505)
China	21.179<br>(-26.982,83.012)	1.75<br>(0.489,5.506)	4.533<br>(-6.253,19.518)	1.682<br>(0.486,5.517)	11.38<br>(-16.388,49.889)	1.678<br>(0.476,5.610)
Colombia	4.637<br>(-317.607,314.795)	1.034<br>(0.251,3.776)	-0.06<br>(-16.703,19.674)	0.995<br>(0.340,3.370)	-0.183<br>(-38.876,45.574)	0.993<br>(0.334,3.435)
Congo	110.895<br>(-468.475,897.587)	1.337<br>(0.348,5.142)	5.749<br>(-20.834,39.922)	1.325<br>(0.432,4.470)	18.199<br>(-68.191,129.021)	1.325<br>(0.423,4.570)
Costa Rica	62.617<br>(-182.616,384.675)	1.457<br>(0.409,5.421)	9.076<br>(-20.754,48.697)	1.49<br>(0.447,4.990)	17.369<br>(-40.780,94.475)	1.491<br>(0.440,5.066)
Croatia	16.674<br>(-13.427,64.134)	1.836<br>(0.627,5.517)	9.597<br>(-9.142,35.308)	1.841<br>(0.599,5.675)	16.278<br>(-15.976,60.630)	1.842<br>(0.591,5.754)
Cuba	55.467<br>(-136.554,341.429)	1.534<br>(0.426,6.270)	10.875<br>(-22.978,-53.874)	1.527<br>(0.459,5.101)	18.331<br>(-39.692,91.789)	1.527<br>(0.453,5.174)

	Best Potential Scenario		Proportional Increase Scenario		Uniform Increase Scenario	
location	absolute difference	relative difference	absolute difference	relative difference	absolute difference	relative difference
Cyprus	23.316	1.997	7.052	1.971	20.872	1.917
	(-16.012,79.275)	(0.663,5.925)	(-5.322,27.829)	(0.621,6.181)	(-15.476,79.217)	(0.609,6.310)
Czechia	21.183	2.008	13.766	2.015	22.551	2.015
	(-12.226,70.428)	(0.700,6.614)	(-8.091,44.588)	(0.678,6.355)	(-13.494,73.789)	(0.671,6.428)
CÔte d'Ivoire	34.397	1.517	4.45	1.513	12.443	1.513
	(-76.715,176.801)	(0.451,5.319)	(-9.342,23.002)	(0.494,4.720)	(-27.404,65.486)	(0.484,4.820)
Democratic People's Republic of Korea	31.876	1.792	4.387	1.82	12.71	1.82
	(-27.999,116.630)	(0.609,5.565)	(-3.722,15.283)	(0.610,5.046)	(-11.298,44.930)	(0.599,5.138)
Democratic Republic of the Congo	31.19	1.295	5.379	1.285	11.003	1.285
	(-161.365,290.565)	(0.335,4.290)	(-23.385,44.805)	(0.382,3.986)	(-48.643,92.988)	(0.376,4.050)
Denmark	63.681	1.906	16.941	1.849	29.018	1.848
	(-42.378,227.126)	(0.645,5.495)	(-15.454,61.410)	(0.586,5.354)	(-27.517,106.026)	(0.577,5.417)
Dominica	41.444	1.707	11.925	1.723	17.979	1.721
	(-42.799,158.040)	(0.556,4.783)	(-12.123,46.773)	(0.613,5.023)	(-18.849,71.162)	(0.607,5.067)
	159.715	1.784	14.609	1.754	28.882	1.755

	Best Potential Scenario		Proportional Increase Scenario		Uniform Increase Scenario	
location	absolute difference	relative difference	absolute difference	relative difference	absolute difference	relative difference
Dominican Republic	(-207.108,662.354)	(0.543,5.896)	(-13.515,58.041)	(0.640,4.791)	(-28.002,116.521)	(0.632,5.048)
Ecuador	107.032	1.56	6.041	1.522	16.449	1.522
	(-295.605,598.741)	(0.410,5.554)	(-12.378,33.412)	(0.455,5.222)	(-34.970,92.647)	(0.447,5.323)
Egypt	61.386	1.714	4.147	1.67	14.92	1.673
	(-76.118,235.899)	(0.580,5.029)	(-3.962,15.560)	(0.601,4.543)	(-14.670,56.815)	(0.590,4.648)
El Salvador	87.538	1.667	14.404	1.698	22.734	1.697
	(-139.816,401.66)	(0.492,5.624)	(-20.168,58.275)	(0.477,5.238)	(-32.774,93.043)	(0.471,5.294)
Equatorial Guinea	56.307	1.31	8.866	1.322	21.85	1.323
	(-198.201,349.021)	(0.414,4.372)	(-28.643,54.089)	(0.426,4.150)	(-72.120,135.431)	(0.418,4.226)
Eritrea	22.799	1.414	2.252	1.354	8.773	1.348
	(-67.156,133.258)	(0.432,4.483)	(-6.236,12.954)	(0.446,4.361)	(-25.491,51.890)	(0.436,4.440)
Estonia	44.359	1.589	8.199	1.525	16.476	1.526
	(-79.555,215.535)	(0.492,5.370)	(-15.778,39.962)	(0.483,4.649)	(-32.484,81.476)	(0.476,4.719)
Eswatini	8.856	1.21	2.463	1.246	4.091	1.244
	(-64.940,85.298)	(0.331,4.263)	(-12.287,21.159)	(0.398,4.426)	(-20.816,35.810)	(0.393,4.480)
Ethiopia	9.456	1.144	0.896	1.133	2.069	1.133

	Best Potential Scenario		Proportional Increase Scenario		Uniform Increase Scenario	
location	absolute difference	relative difference	absolute difference	relative difference	absolute difference	relative difference
	(-80.361,125.678)	(0.353,3.618)	(-8.274,11.469)	(0.362,3.384)	(-19.828,27.166)	(0.355,3.440)
Fiji	12.602	1.26	3.63	1.275	5.571	1.275
	(-63.959,109.893)	(0.420,4.263)	(-15.365,24.754)	(0.419,3.973)	(-24.094,38.499)	(0.415,4.013)
Finland	11.86	1.58	13.371	1.618	29.141	1.618
	(-25.669,62.690)	(0.418,5.293)	(-27.014,68.048)	(0.426,5.429)	(-60.000,150.664)	(0.418,5.519)
France	62.02	1.799	20.46	1.817	34.927	1.817
	(-61.587,252.215)	(0.548,5.835)	(-16.825,70.465)	(0.628,5.318)	(-29.763,121.692)	(0.619,5.389)
Gabon	55.862	1.166	4.282	1.136	11.138	1.137
	(-503.826,684.721)	(0.361,4.025)	(-37.969,50.095)	(0.384,3.361)	(-99.630,131.809)	(0.378,3.426)
Gambia	115.395	2.812	18.631	2.654	51.781	2.651
	(-14.766,350.187)	(0.853,9.108)	(-4.439,58.836)	(0.762,10.109)	(13.612,166.416)	(0.745,10.328)
Georgia	37.403	1.356	4.482	1.374	10.093	1.372
	(-124.692,268.239)	(0.419,4.482)	(-10.544,24.756)	(0.498,3.892)	(-24.520,56.964)	(0.490,3.943)
Germany	10.443	1.545	12.054	1.55	19.021	1.552
	(-14.924,41.917)	(0.564,4.114)	(-15.116,52.712)	(0.594,4.392)	(-24.222,83.921)	(0.590,4.441)
Ghana	47.917	1.384	5.2	1.432	14.272	1.434

	Best Potential Scenario		Proportional Increase Scenario		Uniform Increase Scenario	
location	absolute difference	relative difference	absolute difference	relative difference	absolute difference	relative difference
	(-123.720,256.018)	(0.467,3.985)	(-10.493,25.653)	(0.537,3.810)	(-29.508,71.608)	(0.528,3.879)
Greece	130.248	1.781	16.034	1.698	36.635	1.698
	(-133.032,478.905)	(0.584,5.032)	(-16.547,63.419)	(0.611,4.977)	(-39.693,147.418)	(0.601,5.065)
Grenada	15.676	1.527	5.458	1.537	7.389	1.536
	(-37.469,93.845)	(0.423,5.544)	(-11.544,28.168)	(0.459,5.203)	(-15.794,38.527)	(0.456,5.244)
Guatemala	104.007	1.814	11.306	1.92	21.304	1.92
	(-125.822,427.171)	(0.493,5.855)	(-6.713,32.709)	(0.681,5.029)	(-13.044,62.368)	(0.672,5.095)
Guinea	36.892	1.683	4.23	1.637	11.753	1.639
	(-68.379,177.467)	(0.438,5.489)	(-7.356,20.442)	(0.491,5.031)	(-20.811,57.966)	(0.482,5.131)
Guinea-Bissau	55.329	1.604	6.248	1.68	16.581	1.681
	(-88.804,302.070)	(0.494,5.418)	(-8.545,25.381)	(0.523,5.233)	(-23.248,68.472)	(0.513,5.339)
Guyana	29.773	1.523	4.977	1.572	9.939	1.573
	(-51.523,154.051)	(0.498,5.339)	(-8.209,23.505)	(0.495,4.659)	(-16.770,47.612)	(0.488,4.726)
Haiti	84.065	1.72	8.574	1.788	18.054	1.791
	(-126.813,406.461)	(0.502,5.753)	(-7.001,32.355)	(0.613,4.910)	(-15.146,69.112)	(0.604,4.991)
Honduras	81.168	1.784	8.911	1.739	16.048	1.74

	Best Potential Scenario		Proportional Increase Scenario		Uniform Increase Scenario	
**location**	**absolute difference**	**relative difference**	**absolute difference**	**relative difference**	**absolute difference**	**relative difference**
	(-96.855,324.094)	(0.494,5.693)	(-9.373,39.854)	(0.578,5.723)	(-17.417,72.515)	(0.572,5.804)
Hungary	17.612	1.952	11.257	1.997	19.772	1.997
	(-14.122,62.989)	(0.592,6.525)	(-10.046,41.260)	(0.577,5.885)	(-18.149,73.311)	(0.569,5.962)
Iceland	62.299	1.749	9.117	1.813	25.582	1.814
	(-70.048,316.451)	(0.532,6.446)	(-9.262,35.791)	(0.532,5.868)	(-26.747,101.592)	(0.521,5.991)
India	25.534	1.329	2.955	1.358	6.517	1.358
	(-97.249,186.915)	(0.396,4.300)	(-9.322,16.796)	(0.412,4.051)	(-21.019,37.583)	(0.405,4.115)
Indonesia	34.474	1.412	2.832	1.354	5.691	1.353
	(-120.336,243.068)	(0.363,4.562)	(-8.934,17.006)	(0.424,4.165)	(-18.356,34.735)	(0.417,4.229)
Iran (Islamic Republic of)	152.201	1.724	7.042	1.658	31.37	1.655
	(-218.673,672.247)	(0.477,6.185)	(-9.866,32.936)	(0.513,5.527)	(-46.466,151.544)	(0.500,5.660)
Iraq	54.836	1.654	3.957	1.575	22.456	1.575
	(-96.079,257.688)	(0.487,5.688)	(-6.585,18.145)	(0.526,5.251)	(-38.635,105.886)	(0.512,5.407)
Ireland	37.847	1.598	16.635	1.601	23.509	1.603
	(-61.020,189.205)	(0.489,5.372)	(-26.116,77.233)	(0.505,5.443)	(-38.100,111.789)	(0.502,50500)
Israel	17.73	1.708	11.849	1.588	32.406	1.583

	Best Potential Scenario		Proportional Increase Scenario		Uniform Increase Scenario	
**location**	**absolute difference**	**relative difference**	**absolute difference**	**relative difference**	**absolute difference**	**relative difference**
	(-21.541,79.221)	(0.527,5.317)	(-18.501,52.785)	(0.513,5.233)	(-52.324,145.574)	(0.503,5.341)
Italy	50.691	1.96	16.787	1.926	31.058	1.924
	(-32.140,177.344)	(0.642,6.164)	(-12.358,61.106)	(0.604,5.931)	(-23.507,114.740)	(0.595,6.001)
Jamaica	34.726	1.769	9.273	1.848	15.34	1.847
	(-32.553,151.408)	(0.574,6.434)	(-9.705,37.520)	(0.555,6.069)	(-16.470,62.883)	(0.546,6.145)
Japan	77.004	1.406	4.421	1.41	10.903	1.41
	(-242.923,476.655)	(0.398,4.094)	(-11.151,23.803)	(0.503,4.213)	(-28.206,59.872)	(0.495,4.289)
Jordan	91.978	1.849	7.788	1.86	35.665	1.859
	(-90.522,416.173)	(0.548,6.423)	(-5.745,30.364)	(0.624,5.927)	(-27.627,142.062)	(0.609,6.078)
Kazakhstan	16.221	1.491	4.033	1.579	14.02	1.58
	(-28.139,80.418)	(0.564,4.434)	(-7.357,16.885)	(0.519,4.891)	(-26.498,60.107)	(0.507,5.013)
Kenya	25.331	1.274	3.049	1.327	6.187	1.327
	(-131.280,205.656)	(0.387,4.132)	(-10.224,20.386)	(0.403,4.506)	(-21.134,41.952)	(0.397,4.573)
Kuwait	3.096	1.49	3.378	1.512	27.223	1.512
	(-6.938,16.609)	(0.424,4.752)	(-7.639,17.509)	(0.438,5.203)	(-64.842,144.944)	(0.425,5.351)
Kyrgyzstan	16.376	1.497	2.66	1.554	8.622	1.556

	Best Potential Scenario		Proportional Increase Scenario		Uniform Increase Scenario	
location	absolute difference	relative difference	absolute difference	relative difference	absolute difference	relative difference
	(-42.583,107.818)	(0.415,5.712)	(-5.377,14.829)	(0.481,5.311)	(-17.770,49.032)	(0.472,5.424)
Lao People's Democratic Republic	-4.06	0.945	-0.633	0.9	-1.399	0.9
	(-93.401,81.814)	(0.305,3.014)	(-7.532,6.449)	(0.354,2.605)	(-16.867,14.480)	(0.349,2.640)
Latvia	17.612	1.385	6.776	1.357	12.482	1.357
	(-57.689,114.773)	(0.405,4.604)	(-21.671,42.363)	(0.416,4.486)	(-40.946,79.436)	(0.410,4.544)
Lebanon	48.831	1.505	11.257	1.549	30.858	1.548
	(-102.728,284.485)	(0.497,5.359)	(-21.500,52.694)	(0.500,5.365)	(-61.194,147.048)	(0.490,5.472)
Lesotho	11.558	1.46	4.322	1.474	9.664	1.474
	(-20.270,54.241)	(0.536,4.488)	(-9.763,20.799)	(0.488,4.190)	(-22.551,47.455)	(0.480,4.253)
Liberia	126.969	2.371	13.684	2.44	31.9	2.439
	(-53.961,420.897)	(0.685,8.807)	(-4.064,40.635)	(0.754,6.998)	(-10.244,96.203)	(0.740,7.106)
Libya	12.665	1.396	5.88	1.461	24.362	1.463
	(-29.338,67.990)	(0.483,4.079)	(-12.524,30.122)	(0.468,4.665)	(-54.313,127.564)	(0.458,4.786)
Lithuania	32.945	1.487	8.602	1.431	16.184	1.43
	(-90.574,174.228)	(0.412,5.324)	(-25.245,46.779)	(0.419,4.309)	(-48.590,89.313)	(0.412,4.365)
Luxembourg	40.036	1.821	18.443	1.738	28.503	1.737

	Best Potential Scenario		Proportional Increase Scenario		Uniform Increase Scenario	
location	absolute difference	relative difference	absolute difference	relative difference	absolute difference	relative difference
	(-41.616,162.241)	(0.565,6.087)	(-20.430,73.286)	(0.567,5.419)	(-32.188,114.734)	(0.561,5.477)
Madagascar	41.134	1.362	3.848	1.381	8.994	1.38
	(-174.624,325.027)	(0.336,5.800)	(-12.373,28.580)	(0.411,5.109)	(-29.676,68.378)	(0.404,5.197)
Malawi	10.674	1.437	1.735	1.437	3.824	1.438
	(-32.656,71.779)	(0.375,4.854)	(-4.436,9.044)	(0.446,4.435)	(-10.006,20.313)	(0.437,4.426)
Malaysia	10.132	1.062	3.515	1.197	6.737	1.197
	(-242.619,270.250)	(0.315,3.780)	(-27.574,30.536)	(0.309,3.578)	(-53.605,59.419)	(0.304,3.630)
Mali	32.308	1.59	3.423	1.615	11.457	1.614
	(-49.703,144.288)	(0.512,4.731)	(-4.065,13.666)	(0.577,4.487)	(-14.207,46.756)	(0.566,4.576)
Malta	61.37	1.809	10.264	1.907	31.086	1.907
	(-49.856,215.029)	(0.633,5.573)	(-7.337,35.638)	(0.659,5.999)	(-23.435,109.749)	(0.646,6.123)
Mauritania	15.209	1.238	0.768	1.241	6.341	1.24
	(-62.816,112.230)	(0.451,3.494)	(-3.077,5.545)	(0.475,3.453)	(-26.666,47.533)	(0.463,3.541)
Mauritius	12.633	1.598	6.581	1.568	10.506	1.567
	(-19.600,59.262)	(0.504,4.570)	(-9.805,29.504)	(0.534,4.432)	(-16.057,47.682)	(0.529,4.471)
Mexico	279.746	1.966	12.56	1.925	34.025	1.924

	Best Potential Scenario		Proportional Increase Scenario		Uniform Increase Scenario	
location	absolute difference	relative difference	absolute difference	relative difference	absolute difference	relative difference
	(-349.140,1286.237)	(0.480,7.699)	(-10.487,47.867)	(0.599,5.975)	(-29.710,132.959)	(0.586,6.092)
Mongolia	19.026	1.446	2.833	1.439	14.269	1.439
	(-50.591,106.443)	(0.439,4.719)	(-7.193,16.639)	(0.455,4.581)	(-38.031,86.242)	(0.443,4.991)
Montenegro	38.189	2.089	11.424	2.138	21.153	2.14
	(-21.805,141.081)	(0.692,6.863)	(-5.608,39.207)	(0.699,6.042)	(-10.767,73.611)	(0.690,6.128)
Morocco	38.022	1.472	5.898	1.405	21.148	1.405
	(-99.082,208.151)	(0.419,5.085)	(-15.216,35.268)	(0.459,4.682)	(-56.078,129.082)	(0.450,4.795)
Mozambique	23.577	1.303	3.034	1.274	6.898	1.273
	(-87.286,157.477)	(0.400,4.103)	(-11.931,20.939)	(0.433,3.802)	(-27.789,48.484)	(0.425,3.867)
Myanmar	0.827	1.06	0.327	1.074	0.623	1.072
	(-23.155,23.968)	(0.319,3.412)	(-5.616,60693)	(0.347,3.194)	(-11.147,13.201)	(0.341,3.229)
Namibia	22.181	1.28	1.908	1.278	7.569	1.278
	(-94.603,157.718)	(0.414,3.734)	(-6.841,11.367)	(0.446,3.722)	(-27.972,46.580)	(0.436,3.811)
Nepal	12.089	1.415	2.644	1.427	6.624	1.427
	(-25.102,63.037)	(0.500,4.234)	(-5.742,12.358)	(0.472,4.074)	(-14.860,31.421)	(0.463,4.146)
Netherlands	23.598	1.659	18.216	1.681	29.449	1.681

location	Best Potential Scenario		Proportional Increase Scenario		Uniform Increase Scenario	
	absolute difference	relative difference	absolute difference	relative difference	absolute difference	relative difference
	(-26.219,94.925)	(0.604,4.470)	(-20.338,66.975)	(0.615,4.587)	(-33.852,109.354)	(0.608,4.633)
New Zealand	30.389	1.496	12.659	1.509	18.531	1.509
	(-56.869,160.645)	(0.524,5.072)	(-26.730,63.057)	(0.456,4.540)	(-39.692,93.345)	(0.452,4.578)
Nicaragua	59.262	1.632	8.208	1.615	15.224	1.616
	(-92.677,285.246)	(0.521,5.702)	(-9.765,33.617)	(0.574,4.750)	(-18.686,63.067)	(0.567,4.817)
Niger	5.014	1.633	1.374	1.693	5.683	1.693
	(-5.442,18.889)	(0.587,4.482)	(-1.307,5.124)	(0.631,4.663)	(-5.636,21.727)	(0.617,4.773)
Nigeria	35.644	1.394	3.267	1.401	9.916	1.4
	(-119.177,255.238)	(0.417,4.694)	(-9.103,18.997)	(0.419,4.989)	(-28.646,59.063)	(0.410,5.099)
North Macedonia	68.232	2.245	8.538	2.173	18.094	2.174
	(-33.133,245.044)	(0.705,7.540)	(-4.038,32.340)	(0.687,7.255)	(-8.876,69.597)	(0.676,7.389)
Norway	66.934	1.514	11.414	1.521	20.503	1.521
	(-135.526,358.732)	(0.480,4.756)	(-21.794,55.162)	(0.526,4.578)	(-40.044,100.800)	(0.520,4.639)
Oman	33.409	1.47	4.514	1.455	23.367	1.454
	(-71.103,169.240)	(0.469,4.761)	(-10.231,22.305)	(0.478,4.661)	(-54.725,118.406)	(0.467,4.770)
Pakistan	31.426	1.412	2.866	1.459	9.334	1.46

location	Best Potential Scenario		Proportional Increase Scenario		Uniform Increase Scenario	
	absolute difference	relative difference	absolute difference	relative difference	absolute difference	relative difference
	(-80.397,175.452)	(0.421,4.515)	(-6.136,13.368)	(0.465,4.445)	(-20.647,44.381)	(0.456,4.544)
Palestine	4.083	1.063	1.379	1.051	4.078	1.049
	(-83.539,92.548)	(0.362,2.935)	(-32.765,34.364)	(0.372,2.753)	(-103.686,108.630)	(0.364,2.800)
Panama	69.959	1.619	7.812	1.577	16.39	1.579
	(-121.643,321.683)	(0.453,5.361)	(-12.519,38.022)	(0.507,5.351)	(-26.807,80.949)	(0.499,5.454)
Papua New Guinea	5.296	1.289	0.589	1.207	1	1.207
	(-25.740,39.708)	(0.336,4.212)	(-3.601,5.045)	(0.364,3.723)	(-6.232,8.687)	(0.359,3.773)
Paraguay	83.77	2.46	15.602	2.402	26.999	2.402
	(-29.342,270.676)	(0.737,7.843)	(-4.138,46.613)	(0.788,6.629)	(-7.668,81.468)	(0.778,6.710)
Peru	131.626	1.772	3.726	1.735	15.495	1.733
	(-221.917,666.097)	(0.437,6.718)	(-4.502,14.763)	(0.542,5.200)	(-19.425,62.753)	(0.529,5.318)
Philippines	16.412	1.175	1.291	1.134	2.693	1.134
	(-146.739,210.510)	(0.310,4.464)	(-12.562,18.499)	(0.342,3.817)	(-26.650,39.237)	(0.337,3.884)
Poland	6.117	1.206	2.337	1.245	4.078	1.245
	(-38.539,55.769)	(0.374,3.957)	(-12.464,18.353)	(0.359,3.771)	(-22.101,32.516)	(0.354,3.814)
Portugal	141.099	2.065	20.43	2.038	38.921	2.038

	Best Potential Scenario		Proportional Increase Scenario		Uniform Increase Scenario	
**location**	**absolute difference**	**relative difference**	**absolute difference**	**relative difference**	**absolute difference**	**relative difference**
	(-98.948,560.375)	(0.627,7.325)	(-11.213,71.235)	(0.696,6.758)	(-22.292,137.399)	(0.687,6.867)
Puerto Rico	37.935	1.438	9.413	1.41	13.232	1.409
	(-91.739,208.052)	(0.429,4.699)	(-23.366,61.432)	(0.475,4.941)	(-33.406,87.286)	(0.471,4.983)
Qatar	4.045	1.368	3.113	1.473	28.067	1.476
	(-10.821,25.873)	(0.473,4.170)	(-6.937,14.291)	(0.444,4.325)	(-65.231,134.386)	(0.430,4.455)
Republic of Korea	9.625	1.525	4.378	1.468	10.808	1.47
	(-17.781,47.633)	(0.486,5.022)	(-8.836,23.231)	(0.506,4.452)	(-22.539,58.591)	(0.497,4.533)
Republic of Moldova	7.249	1.547	4.058	1.46	8.333	1.457
	(-11.021,32.019)	(0.533,4.324)	(-8.203,20.814)	(0.541,4.297)	(-17.484,43.554)	(0.532,4.350)
Romania	19.575	1.899	6.963	1.889	14.477	1.888
	(-13.576,71.958)	(0.664,5.792)	(-6.041,25.224)	(0.617,5.853)	(-13.101,53.185)	(0.608,5.937)
Russian Federation	12.523	1.257	3.345	1.253	8.33	1.253
	(-68.382,108.612)	(0.368,3.993)	(-17.753,27.498)	(0.370,4.062)	(-45.657,70.351)	(0.362,4.140)
Rwanda	20.586	1.822	4.338	1.868	8.374	1.869
	(-18.855,78.574)	(0.588,5.581)	(-3.272,17.631)	(0.656,5.857)	(-6.556,34.485)	(0.647,5.950)
	18.991	1.732	6.312	1.725	10.407	1.725

	Best Potential Scenario		Proportional Increase Scenario		Uniform Increase Scenario	
**location**	**absolute difference**	**relative difference**	**absolute difference**	**relative difference**	**absolute difference**	**relative difference**
Saint Kitts and Nevis	(-23.478,78.223)	(0.593,5.162)	(-6.713,25.421)	(0.556,5.317)	(-11.432,42.459)	(0.550,5.387)
Saint Lucia	3.169	1.448	2.435	1.499	4.051	1.501
	(-6.082,16.608)	(0.521,4.366)	(-4.224,11.251)	(0.528,4.570)	(-7.118,18.878)	(0.523,4.631)
Saint Vincent and the Grenadines	12.036	1.413	6.164	1.366	8.937	1.367
	(-28.418,59.242)	(0.498,4.321)	(-19.192,32.461)	(0.440,3.994)	(-28.151,47.516)	(0.437,4.034)
Saudi Arabia	43.069	1.517	3.035	1.534	26.368	1.536
	(-90.925,195.036)	(0.437,4.687)	(-5.570,16.116)	(0.495,4.791)	(-50.146,144.248)	(0.483,4.927)
Senegal	22.145	1.257	2.031	1.292	8.36	1.288
	(-101.049,186.911)	(0.410,4.414)	(-6.569,11.930)	(0.458,3.745)	(-28.496,50.493)	(0.448,3.817)
Serbia	13.306	2.001	9.141	2.051	17.17	2.051
	(-11.010,51.175)	(0.563,6.802)	(-6.860,31.960)	(0.613,6.260)	(-13.258,60.811)	(0.604,6.350)
Sierra Leone	44.896	1.732	6.118	1.727	16.009	1.729
	(-54.192,183.992)	(0.487,5.673)	(-7.979,26.197)	(0.470,5.273)	(-21.730,69.702)	(0.461,5.374)
Singapore	3.85	1.443	5.183	1.433	11.643	1.434
	(-7.509,18.462)	(0.476,4.061)	(-11.578,26.925)	(0.497,4.354)	(-26.635,61.372)	(0.489,4.428)
Slovakia	22.828	2.125	9.874	2.214	16.978	2.211

	Best Potential Scenario		Proportional Increase Scenario		Uniform Increase Scenario	
location	absolute difference	relative difference	absolute difference	relative difference	absolute difference	relative difference
	(-13.089,86.708)	(0.662,6.887)	(-3.699,31.373)	(0.729,6.815)	(-6.700,54.565)	(0.719,6.889)
Slovenia	55.791	2.313	14.204	2.36	22.609	2.362
	(-17.995,196.620)	(0.772,7.590)	(-3.348,45.038)	(0.785,7.122)	(-5.677,72.328)	(0.777,7.209)
Somalia	93.144	1.584	3.595	1.519	13.957	1.519
	(-244.891,551.445)	(0.369,6.464)	(-7.479,19.611)	(0.453,4.779)	(-30.030,78.274)	(0.440,4.901)
South Africa	54.745	1.484	8.406	1.456	18.393	1.454
	(-130.320,292.039)	(0.464,4.386)	(-15.726,42.641)	(0.535,4.328)	(-35.637,95.034)	(0.526,4.395)
South Sudan	14.237	1.448	2.881	1.398	5.173	1.396
	(-39.139,84.528)	(0.446,4.718)	(-7.178,15.150)	(0.480,4.071)	(-13.171,27.597)	(0.474,4.123)
Spain	142.567	2.08	21.923	2.116	49.947	2.118
	(-102.520,507.404)	(0.654,7.102)	(-8.294,72.887)	(0.778,6.170)	(-19.643,168.263)	(0.767,6.282)
Sri Lanka	1.102	1.08	0.472	1.11	0.756	1.111
	(-17.585,19.522)	(0.339,3.533)	(-5.554,7.376)	(0.365,3.489)	(-8.937,11.889)	(0.361,3.532)
Sudan	14.27	1.207	0.978	1.209	5.095	1.207
	(-87.773,141.820)	(0.373,4.257)	(-5.675,8.681)	(0.416,3.796)	(-30.771,46.249)	(0.406,3.881)
Suriname	94.497	1.542	13.38	1.627	25.238	1.628

location	Best Potential Scenario		Proportional Increase Scenario		Uniform Increase Scenario	
	absolute difference	relative difference	absolute difference	relative difference	absolute difference	relative difference
	(-183.094,462.194)	(0.479,4.932)	(-18.883,55.776)	(0.516,4.618)	(-36.286,106.707)	(0.509,4.680)
Sweden	118.971	2.302	25.537	2.259	49.233	2.259
	(-61.816,418.587)	(0.651,7.805)	(-12.464,92.826)	(0.707,6.854)	(-25.020,181.548)	(0.697,6.939)
Switzerland	32.7	1.603	14.8	1.635	22.294	1.635
	(-47.190,140.690)	(0.527,5.396)	(-20.789,64.728)	(0.524,5.269)	(-32.116,98.689)	(0.518,5.324)
Syrian Arab Republic	57.513	1.455	3.98	1.468	14.65	1.468
	(-176.483,363.511)	(0.407,5.015)	(-8.768,22.919)	(0.428,5.484)	(-33.306,86.144)	(0.417,5.629)
Tajikistan	8.594	1.819	2.221	1.788	6.779	1.788
	(-7.659,35.575)	(0.561,5.785)	(-3.047,9.996)	(0.536,5.992)	(-9.600,31.147)	(0.524,6.122)
Thailand	19.688	1.23	1.903	1.241	4.196	1.24
	(-145.509,190.319)	(0.353,4.495)	(-9.243,14.999)	(0.367,3.730)	(-20.792,33.621)	(0.360,3.791)
Timor-Leste	4.992	1.202	1.165	1.229	2.661	1.229
	(-31.776,50.836)	(0.381,4.121)	(-5.940,8.982)	(0.394,3.905)	(-13.800,-20.874)	(0.387,3.974)
Togo	46.119	1.479	4.089	1.45	11.219	1.45
	(-102.208,239.884)	(0.456,4.605)	(-8.000,19.556)	(0.525,4.383)	(-22.696,54.733)	(0.515,4.468)
	28.068	1.452	5.971	1.395	10.557	1.394

	Best Potential Scenario		Proportional Increase Scenario		Uniform Increase Scenario	
location	absolute difference	relative difference	absolute difference	relative difference	absolute difference	relative difference
Trinidad and Tobago	(-77.804,144.855)	(0.447,4.675)	(-18.714,33.781)	(0.437,4.465)	(-33.692,60.777)	(0.430,4.526)
Tunisia	87.063	1.36	5.989	1.389	21.368	1.389
	(-315.179,668.044)	(0.419,5.125)	(-18.369,34.947)	(0.403,4.301)	(-67.920,127.859)	(0.393,4.399)
Turkey	181.915	1.792	9.254	1.72	27.232	1.724
	(-260.147,827.100)	(0.477,6.412)	(-12.630,40.825)	(0.538,5.912)	(-38.215,122.539)	(0.528,6.050)
Turkmenistan	2.593	1.576	3.418	1.692	13.358	1.694
	(-4.051,11.089)	(0.526,4.644)	(-3.958,13.250)	(0.573,4.596)	(-16.232,52.929)	(0.562,4.693)
Uganda	21.888	1.295	3.528	1.259	6.16	1.26
	(-110.513,200.274)	(0.351,5.220)	(-20.065,34.041)	(0.337,4.867)	(-35.620,60.708)	(0.333,4.937)
Ukraine	25.687	1.444	8.467	1.454	16.319	1.456
	(-63.230,138.547)	(0.460,4.287)	(-23.087,44.652)	(0.452,4.143)	(-45.158,87.078)	(0.446,4.201)
United Arab Emirates	7.64	1.382	2.844	1.358	18.851	1.356
	(-22.488,45.143)	(0.414,4.641)	(-7.625,16.713)	(0.446,4.419)	(-52.421,113.588)	(0.435,4.531)
United Kingdom	35.754	1.386	13.329	1.404	20.741	1.405
	(-133.114,244.020)	(0.376,5.103)	(-41.700,93.267)	(0.421,5.213)	(-65.693,146.600)	(0.417,5.279)
	34.725	1.500	4.672	1.454	9.609	1.451

	Best Potential Scenario		Proportional Increase Scenario		Uniform Increase Scenario	
location	absolute difference	relative difference	absolute difference	relative difference	absolute difference	relative difference
United Republic of Tanzania	(-74.999,180.534)	(0.444,4.967)	(-10.296,27.581)	(0.450,4.757)	(-21.758,57.813)	(0.442,4.829)
United States of America	174.959	1.822	20.265	1.831	38.748	1.829
	(-194.159,637.681)	(0.543,6.322)	(-21.347,77.411)	(0.568,5.608)	(-42.718,150.868)	(0.558,5.694)
United States Virgin Islands	16.243	1.556	15.594	1.582	20.679	1.583
	(-33.491,90.470)	(0.452,5.305)	(-31.292,86.086)	(0.458,5.730)	(-42.348,114.894)	(0.455,5.778)
Uruguay	32.857	1.882	14.406	1.808	27.559	1.803
	(-33.298,135.225)	(0.570,6.236)	(-16.129,61.939)	(0.539,6.367)	(-31.869,120.398)	(0.530,6.449)
Uzbekistan	11.067	1.514	3.622	1.539	11.581	1.540
	(-19.464,55.101)	(0.511,4.267)	(-6.597,16.103)	(0.500,4.586)	(-21.948,52.483)	(0.488,4.693)
Venezuela (Bolivarian Republic of)	92.223	1.448	6.635	1.403	15.29	1.403
	(-262.499,572.772)	(0.431,5.076)	(-18.513,36.480)	(0.465,4.395)	(-43.767,85.579)	(0.457,4.473)
Viet Nam	25.685	1.661	2.064	1.591	5.593	1.592
	(-42.590,132.592)	(0.461,6.179)	(-3.399,9.665)	(0.493,5.356)	(-9.532,26.652)	(0.483,5.466)
Yemen	79.707	1.493	2.125	1.494	11.146	1.494
	(-200.404,467.076)	(0.389,5.800)	(-5.629,12.206)	(0.396,4.974)	(-30.458,66.045)	(0.385,5.115)
Zambia	21.155	1.253	2.82	1.277	7.010	1.275

	Best Potential Scenario		Proportional Increase Scenario		Uniform Increase Scenario	
**location**	**absolute difference**	**relative difference**	**absolute difference**	**relative difference**	**absolute difference**	**relative difference**
	(-117.132,175.598)	(0.362,3.896)	(-11.723,19.004)	(0.420,3.951)	(-30.359,48.471)	(0.411,4.020)
Zimbabwe	6.899	1.252	1.594	1.244	3.493	1.243
	(-27.556,53.301)	(0.450,4.225)	(-6.759,13.055)	(0.421,4.435)	(-15.421,29.146)	(0.413,4.520)

Figure S1. NDVI sampling and analysis of 30,887 cities around the world in 2020.

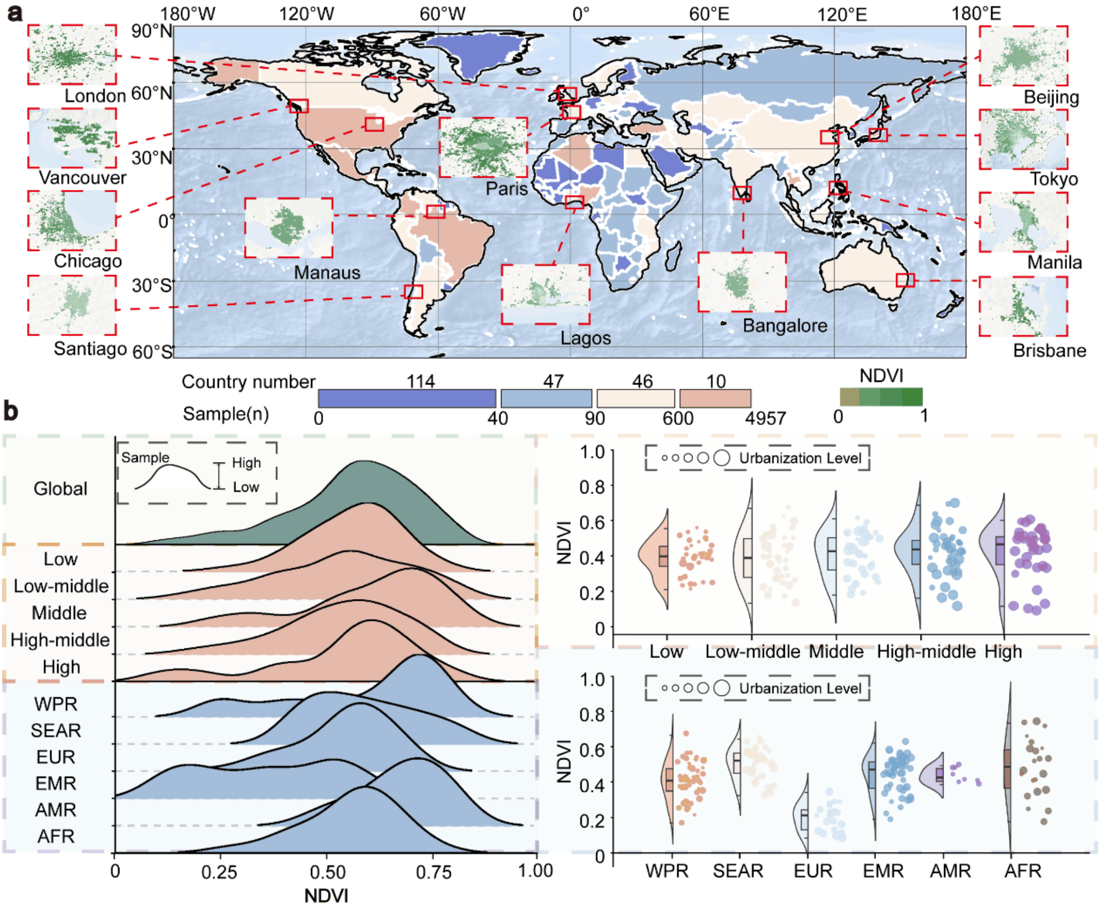

Notes: a, the number of cities sampled in each country and the NDVI of some important cities. **B,** the urban NDVI is distributed in the WHO area and the SDI area. NDVI, normalized difference vegetation index. AFR, African region. AMR, American region. EMR, Eastern Mediterranean region. EUR, European region. SEAR, Southeast Asian region. WPR, Western Pacific region. SDI, socio-demographic index.

Figure S2. Changes in NDVI Values for the Five Most Populous Cities from 2001 to 2020.

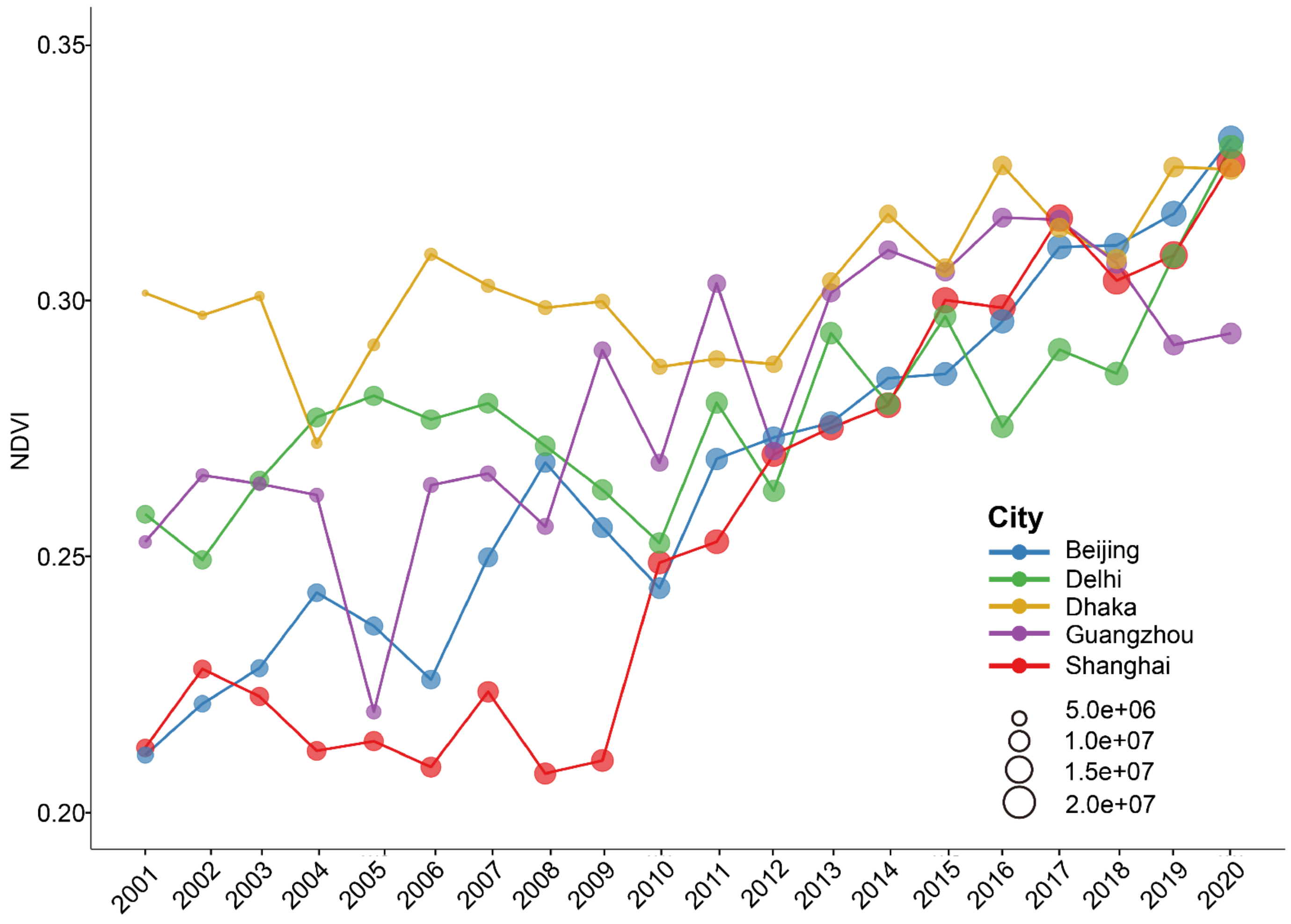

Figure S3. PRISMA flow chart of the study selection and exclusion.

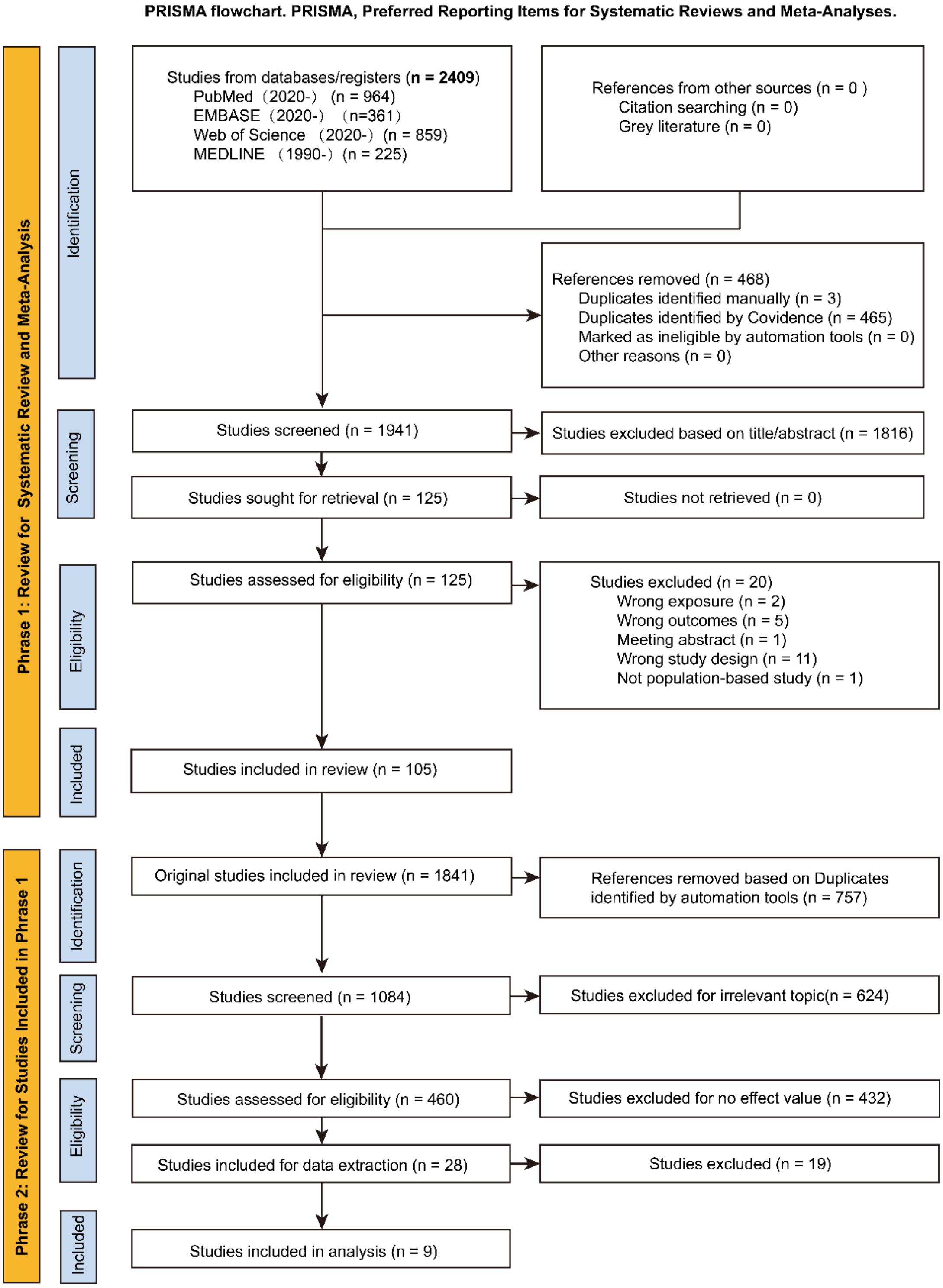

Figure S4. Odd ratio (OR) included in the study and the relationship between city-level NDVI lift and PAF.

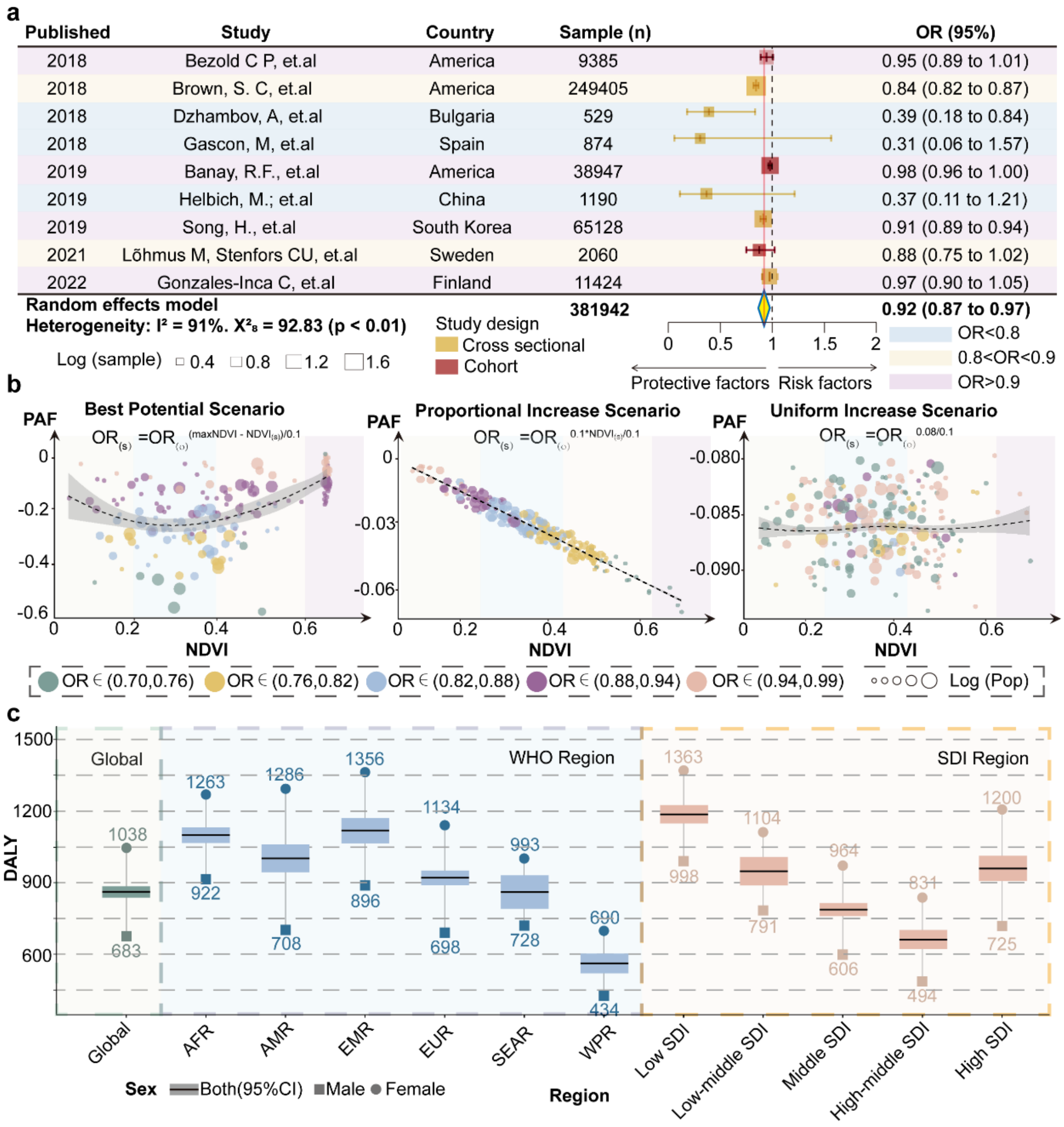

Notes: a, basic information and effect estimates included in the study. **b,** changes in PAF response from city-level NDVI enhancement under three scenarios. **c,** DALY values and gender differences globally and under WHO and SDI partitions. DALY, disability-adjusted life years.

Figure S5. Funnel plot in the pooled effect of greenness exposure on depression.

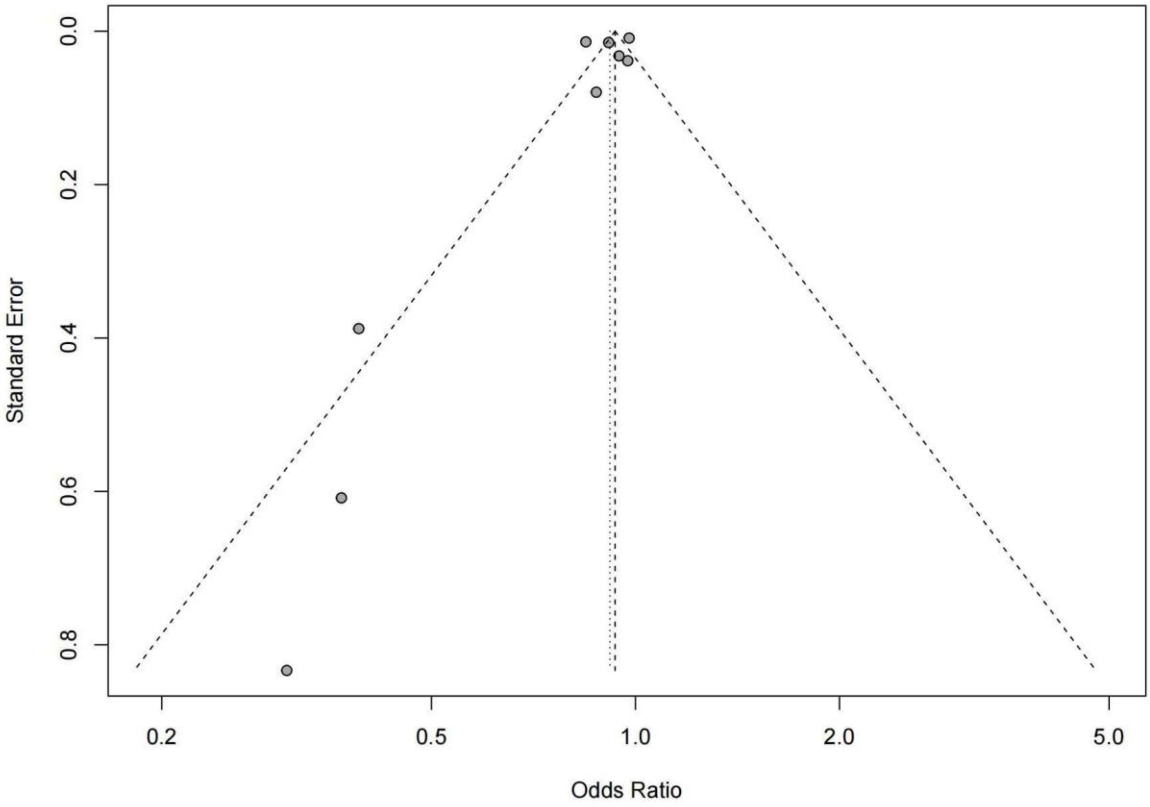

Figure S6. Leave-one-out sensitivity analysis of the influence of a single study on the pooled effect of greenness exposure on depression.

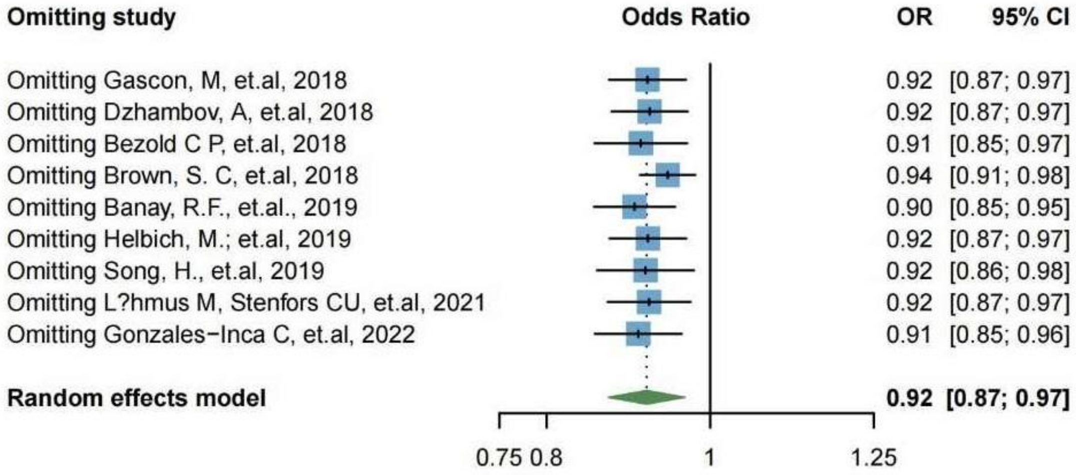

Figure S7. Global Distribution of ASDR for preventable ASDR of depressive disorders attributable to greenness exposure in 2020 and AAPC from 2001 to 2020 in proportional increase scenario.

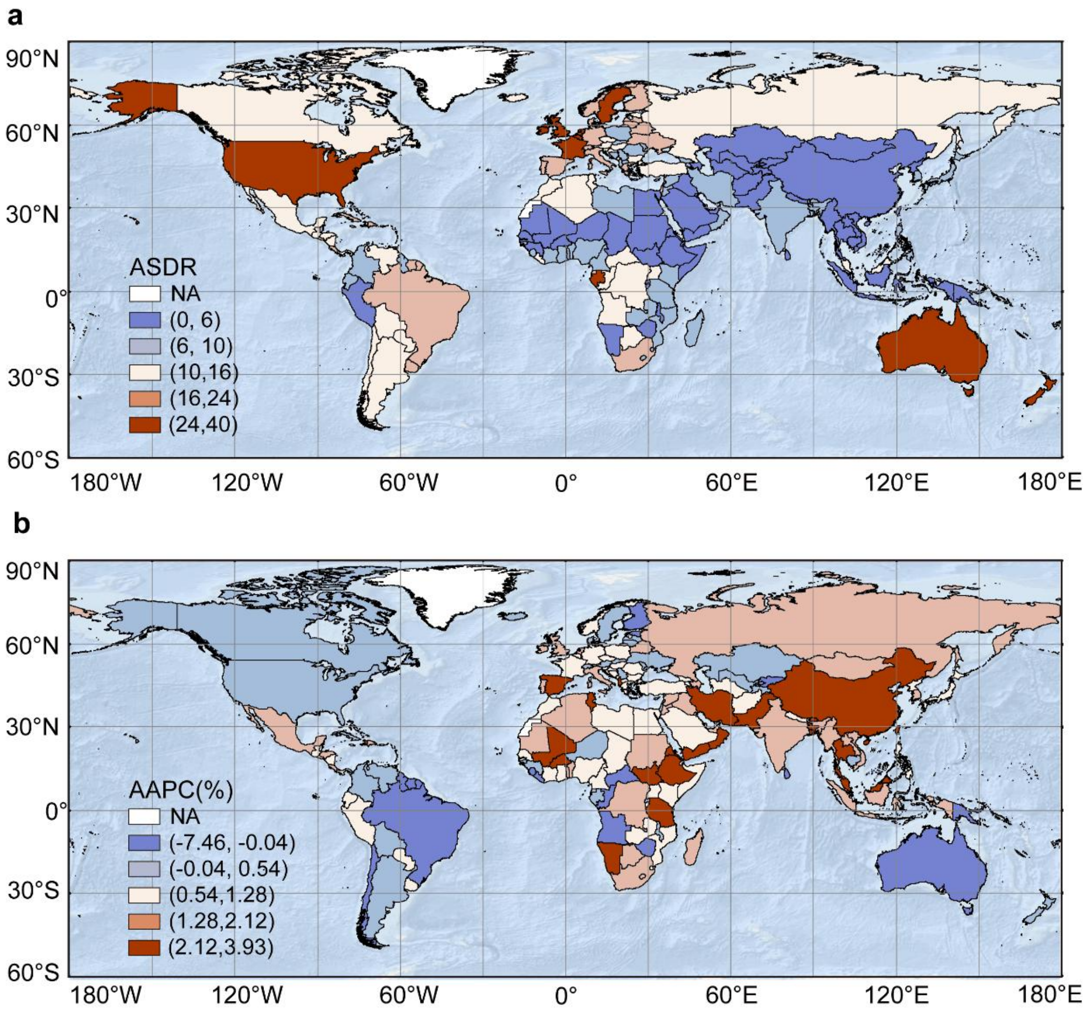

Notes: ASDR, age-standardised DALY rates. AAPC, average annual percent change.

Figure S8. Global Distribution of sex difference of ASDR for preventable ASDR of depressive disorders attributable to greenness exposure in 2020 and AAPC from 2001 to 2020 in proportional increase scenario.

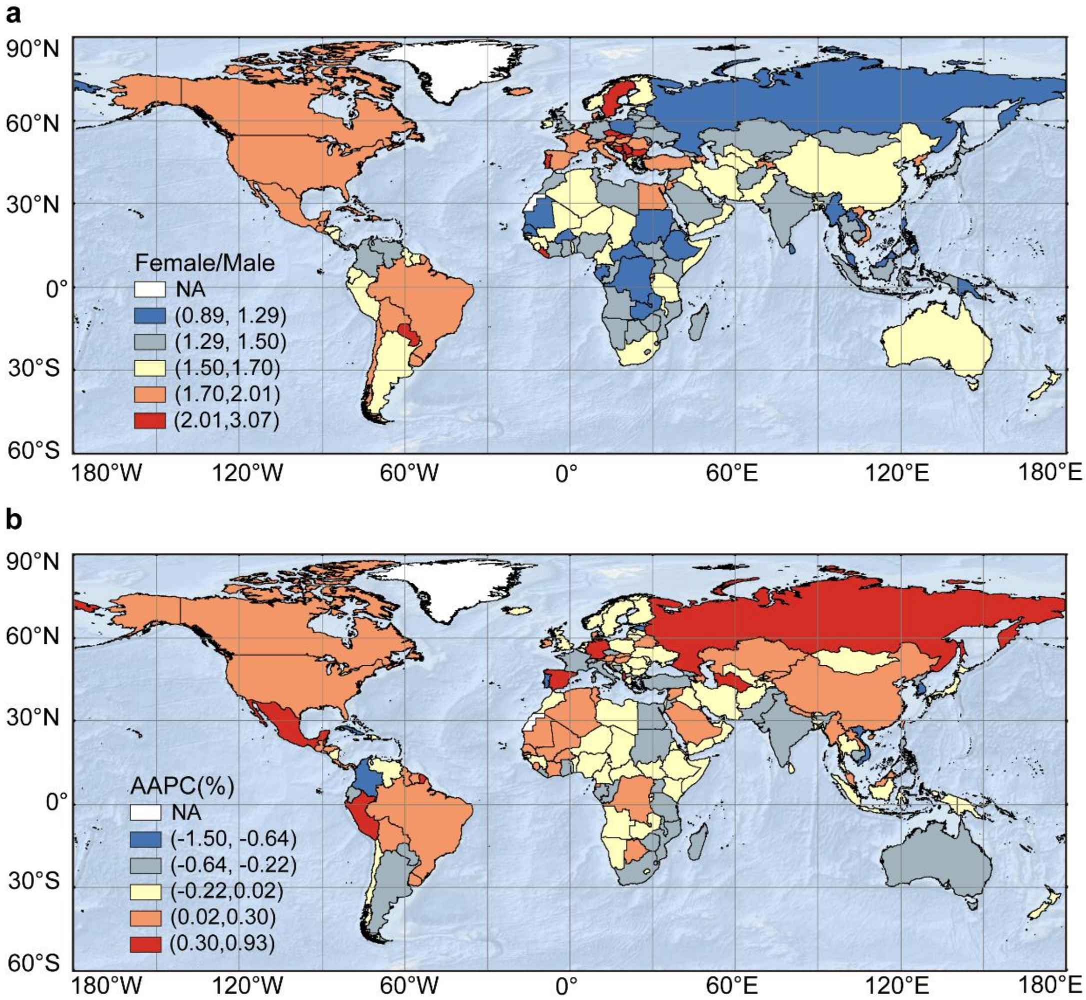

Notes: ASDR, age-standardised DALY rates. AAPC, average annual percent change.

Figure S9. Global Distribution of ASDR for preventable ASDR of depressive disorders attributable to greenness exposure in 2020 and AAPC from 2001 to 2020 in uniform increase scenario.

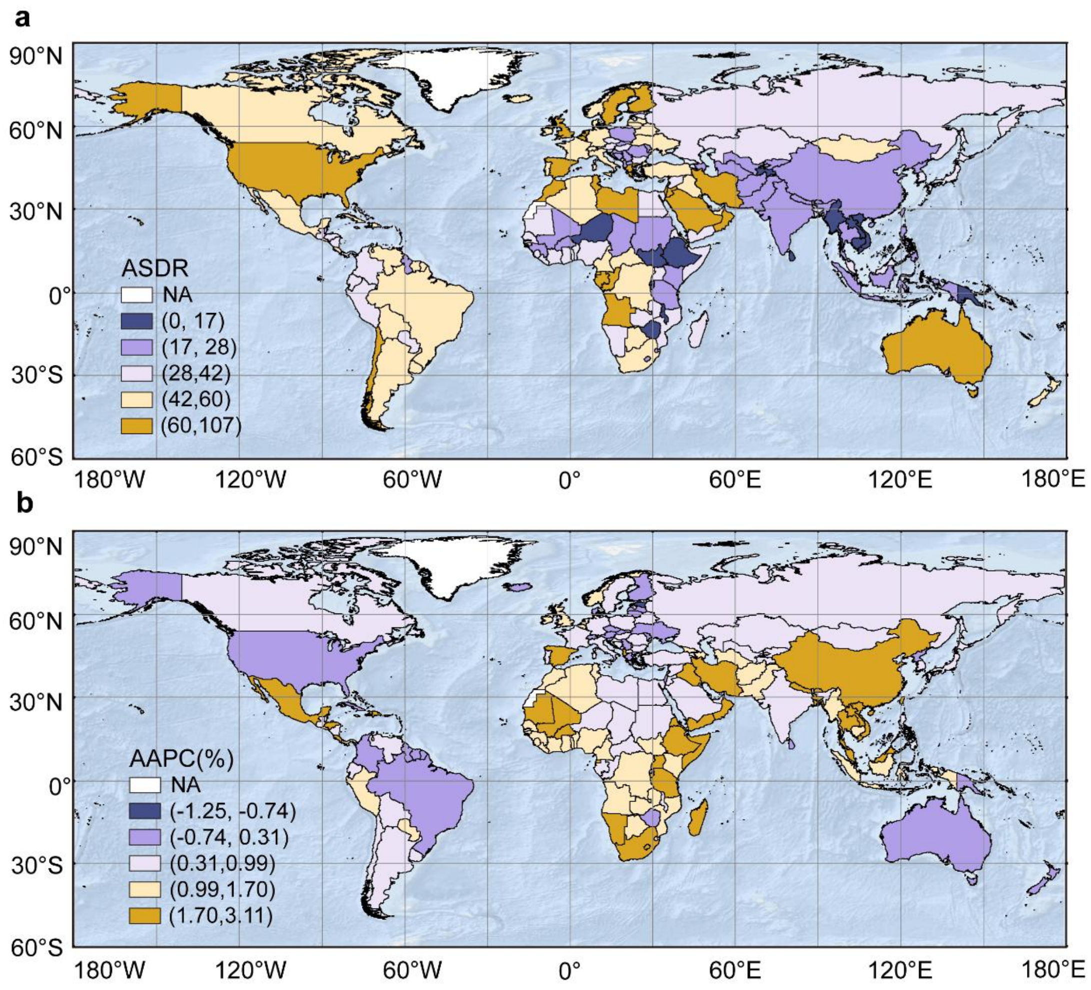

Notes: ASDR, age-standardised DALY rates. AAPC, average annual percent change.

Figure S10. Global Distribution of sex difference of ASDR for preventable ASDR of depressive disorders attributable to greenness exposure in 2020 and AAPC from 2001 to 2020 in uniform increase scenario.

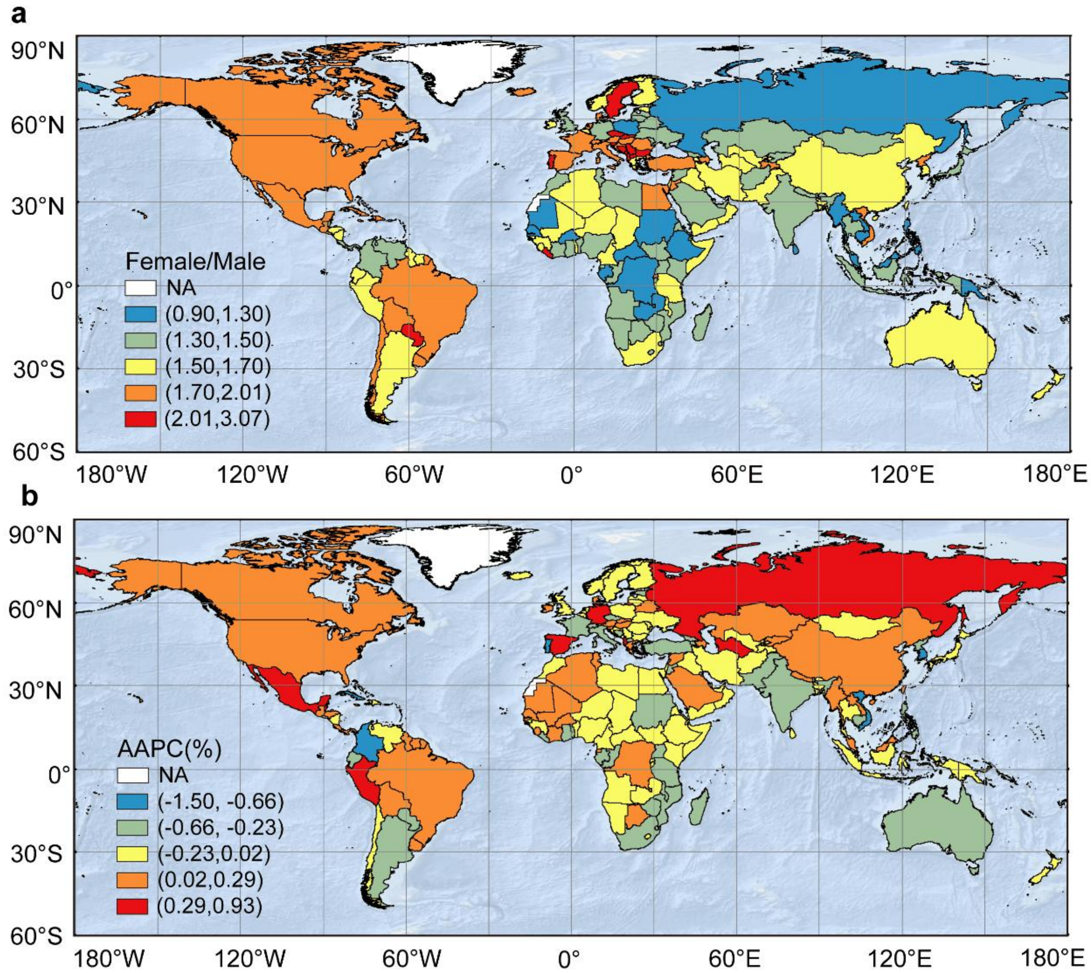

Notes: ASDR, age-standardised DALY rates. AAPC, average annual percent change.

Figure S11. Absolute and relative cross-country inequality in preventable ASDR of depressive disorders attributable to greenness expansion in 2001 and 2020, with the temporal trend of inequality in preventable ASDR in Guinea from 2001 to 2020, under the best potential scenario.

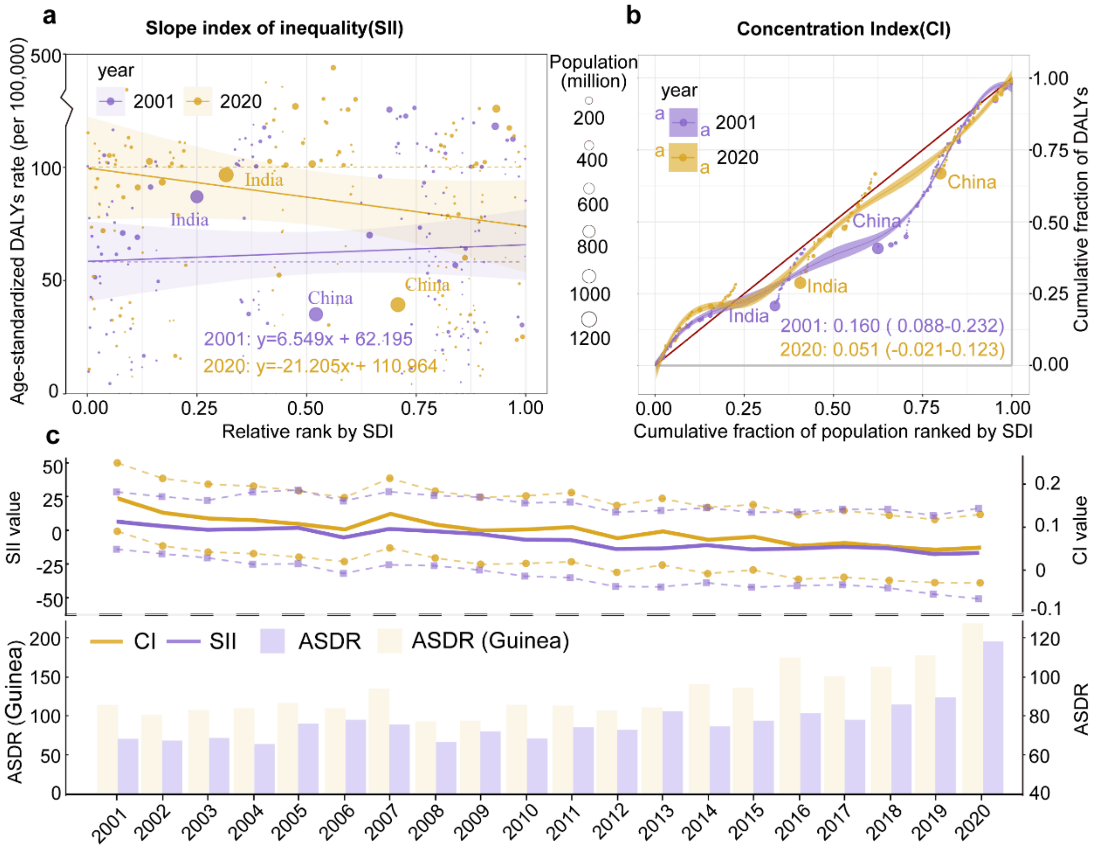

Notes: a, health inequality regression curves for preventable ASDR of depressive disorders attributable to greenness expansion in 2001 and 2020. **b,** concentration curves for preventable ASDR of depressive disorders attributable to greenness expansion in 2001 and 2020. **c,** the temporal trend of absolute and relative inequality in preventable burden of depressive disorders attributable to greenness expansion in Guinea from 2001 to 2020.
ASDR, age-standardized DALY rate. DALY, disability-adjusted life years. SII, slope index of inequality. CI, concentration index. SDI, socio-demographic index.

Figure S12. Absolute and relative cross-country inequality in preventable ASDR of depressive disorders attributable to greenness expansion in 2001 and 2020 under the proportional increase scenario.

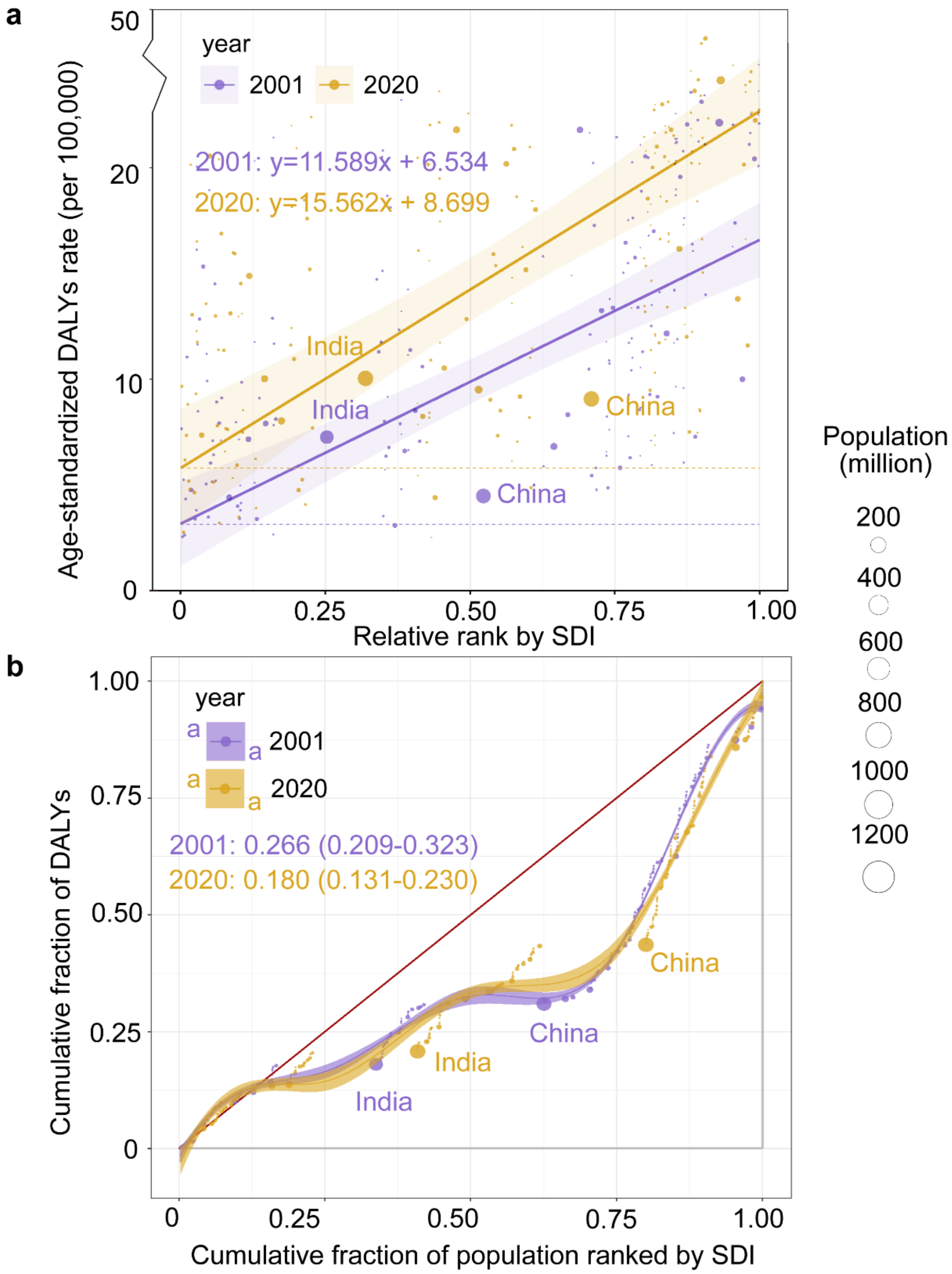

Notes: a, health inequality regression curves for preventable ASDR of depressive disorders attributable to greenness expansion in 2001 and 2020. **b,** concentration curves for preventable ASDR of depressive disorders attributable to greenness expansion in 2001 and 2020. ASDR, age-standardized DALY rate. DALY, disability-adjusted life years. SII, slope index of inequality. CI, concentration index. SDI, socio-demographic index.

Figure S13. Absolute and relative cross-country inequality in preventable ASDR of depressive disorders attributable to greenness expansion in 2001 and 2020 under the uniform increase scenario.

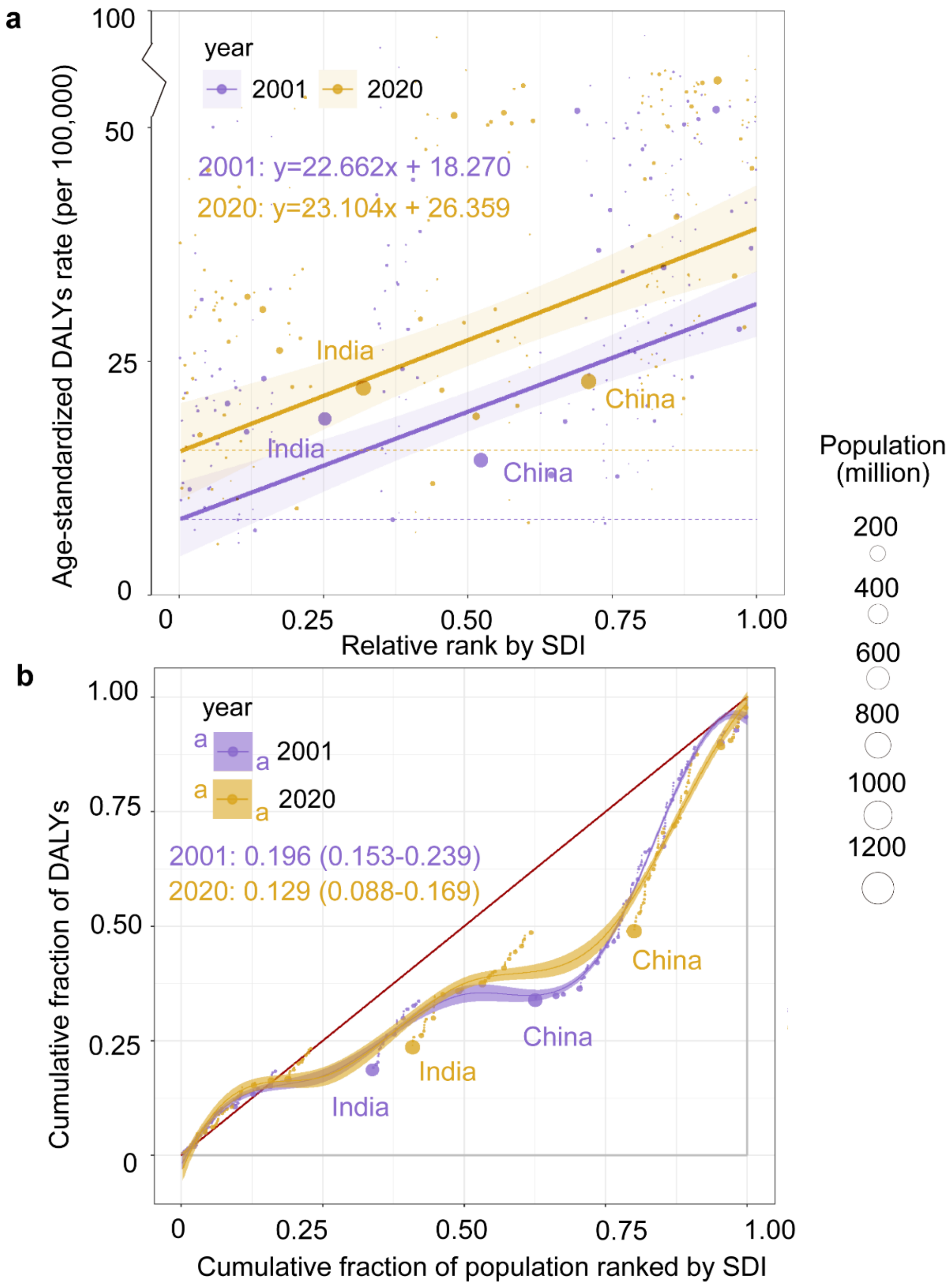

Notes: a, health inequality regression curves for preventable ASDR of depressive disorders attributable to greenness expansion in 2001 and 2020. **b,** concentration curves for preventable ASDR of depressive disorders attributable to greenness expansion in 2001 and 2020. ASDR, age-standardized DALY rate. DALY, disability-adjusted life years. SDI, socio-demographic index.

Figure S14. World average ASDR and ASDR for countries in different SDI phases from 2001 to 2020 under the bset potential scenario.

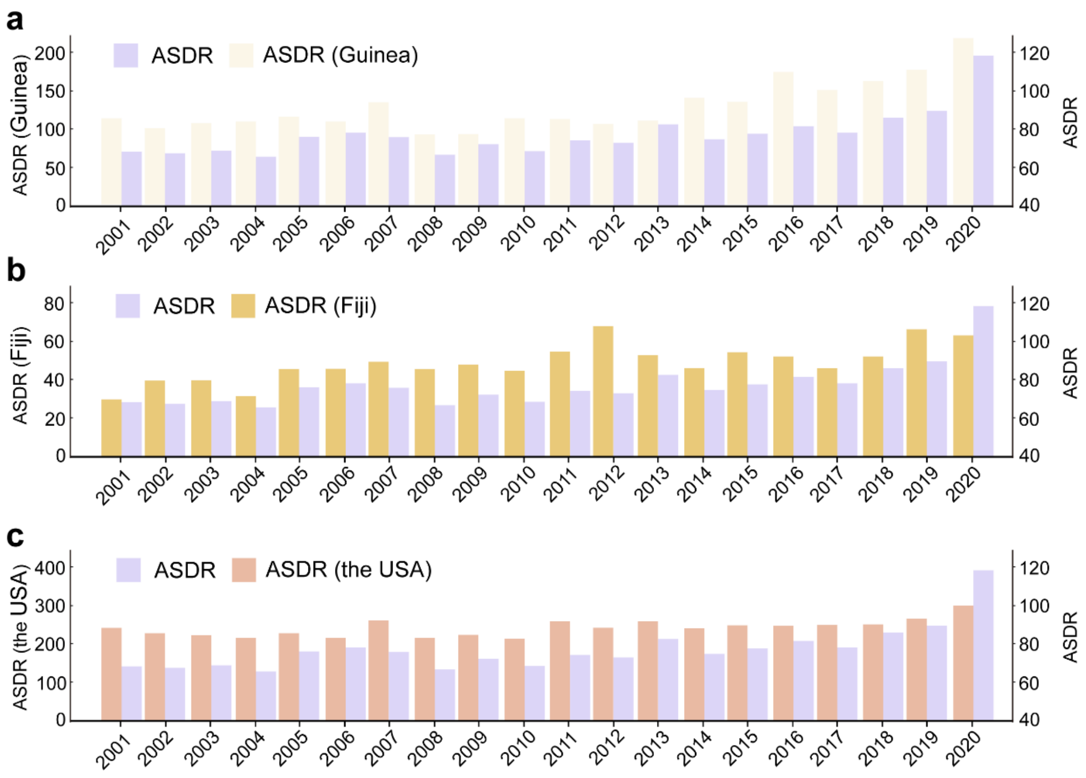

Notes: **a**, Guinea among low SDI countries. **b**, Fiji among middle SDI countries. **c**, the USA among high SDI countries. ASDR, age-standardized DALY rate. DALY, disability-adjusted life years. SDI, socio-demographic index.
